# Tracking and mitigating imprint erasure during induction of naive human pluripotency at single-cell resolution

**DOI:** 10.1016/j.stemcr.2025.102419

**Published:** 2025-02-13

**Authors:** Laura A. Fischer, Brittany Meyer, Monica Reyes, Joseph E. Zemke, Jessica K. Harrison, Kyoung-mi Park, Ting Wang, Harald Jüppner, Sabine Dietmann, Thorold W. Theunissen

**Affiliations:** 1 Department of Developmental Biology and Center of Regenerative Medicine, Washington University School of Medicine, St. Louis, MO, USA; 2 Endocrine Unit, Department of Medicine and Pediatric Nephrology Unit, Department of Pediatrics, Massachusetts General Hospital and Harvard Medical School, Boston, MA, USA; 3 Department of Genetics, The Edison Family Center for Genome Sciences & Systems Biology, Washington University School of Medicine, St. Louis, MO, USA; 4 Institute for Informatics (I^2^), Washington University School of Medicine, St. Louis, MO, USA

**Keywords:** naive pluripotency, stem cells, imprinting, DNA methylation, SNRPN, FGF signaling, ZFP57

## Abstract

Naive human pluripotent stem cells (hPSCs) model the pre-implantation epiblast. However, parent-specific epigenetic marks (imprints) are eroded in naive hPSCs, which represents an important deviation from the epiblast *in vivo*. To track the dynamics of imprint erasure during naive resetting in real time, we established a dual-colored fluorescent reporter at both alleles of the imprinted *SNRPN* locus. During primed-to-naive resetting, SNRPN expression becomes biallelic in most naive cells, and biallelic SNRPN expression is irreversible upon re-priming. We utilized this live-cell reporter to evaluate chemical and genetic strategies to minimize imprint erasure. Decreasing the level of MEK/ERK inhibition or overexpressing the KRAB zinc-finger protein ZFP57 protected a subset of imprints during naive resetting. Combining these two strategies protected imprint levels to a further extent than either strategy alone. This study offers an experimental tool to track and enhance imprint stability during transitions between human pluripotent states *in vitro*.

## Highlights

• A biallelic, dual-colored fluorescent reporter at the imprinted SNRPN locus in hPSCs
• Biallelic SNRPN expression is rapidly induced during primed-to-naive resetting
• Acquisition of biallelic SNRPN expression is irreversible upon re-priming
• ZFP57 overexpression mitigates imprint erasure during primed-to-naive resetting

## Graphical abstract

**Figure:**
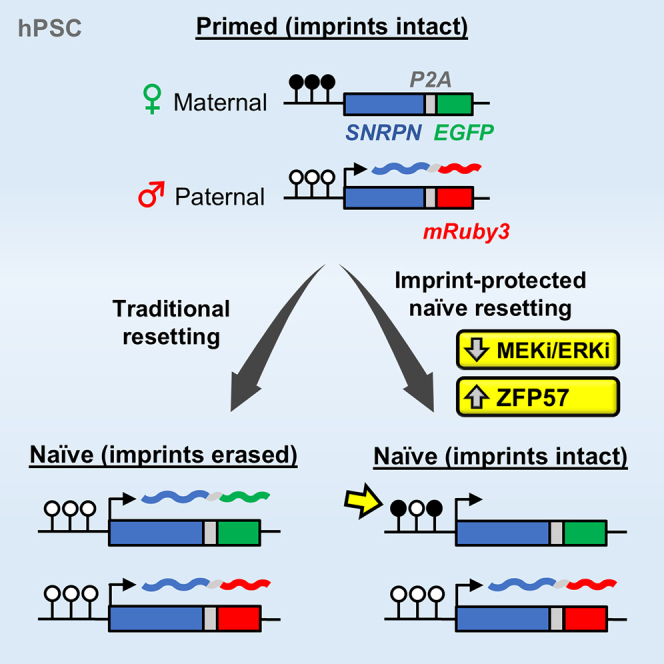

## Teaser

Fischer et al. describe a biallelic, dual-colored fluorescent reporter at an imprinted locus in hPSCs, which monitors imprint stability at single-cell resolution. They report that overexpression of ZFP57 or titration of FGF pathway inhibitors mitigates imprint erasure during primed-to-naive resetting.

## Introduction

Parent-specific epigenetic marks (imprints) are crucial for normal growth and development, yet their mechanisms of establishment and maintenance are not fully understood. Landmark studies in mice demonstrated that imprinted genes are expressed from either the maternal or paternal allele and are essential for the development of embryonic and extraembryonic tissues (Barton et al., 1984; McGrath and Solter, 1984; Surani et al., 1984). In humans, approximately 200 imprinted genes have been discovered, and improper imprinting can manifest as growth restriction, obesity, intellectual disabilities, behavioral abnormalities, and an increased risk of certain cancers (Kalish et al., 2014; Monk et al., 2019; Peters, 2014; Tucci et al., 2019). In addition, aberrant expression of imprinted genes has been implicated in developmental defects in non-human primate embryos generated by somatic cell nuclear transfer (Liao et al., 2024). The evolution of genomic imprinting in placental mammals is thought to reflect the competition between maternal and paternal genomes over resources during gestation (Wilkins and Haig, 2003). Moreover, imprinting promotes the exchange of genetic information by raising barriers to uniparental reproduction (Li et al., 2018).

Advances in modeling early human development have surged from the delineation of pluripotent cell states, namely that of naive and primed pluripotency (Nichols and Smith, 2009). Naive human pluripotent stem cells (hPSCs) align closely with the *in vivo* pre-implantation epiblast in that they share a similar transcriptional profile (including that of transposable elements) (Takashima et al., 2014; Theunissen et al., 2014, 2016), demonstrate X chromosome dampening (Dror et al., 2024; Sahakyan et al., 2017), and possess the developmental plasticity to generate embryonic and extraembryonic tissues (Castel et al., 2020; Cinkornpumin et al., 2020; Dattani et al., 2024; Dong et al., 2020; Guo et al., 2021; Io et al., 2021; Okubo et al., 2024). However, a persistent issue hampering *bona fide* naive hPSCs is the erosion of imprints (Pastor et al., 2016; Theunissen et al., 2016). Current naive culture media require fibroblast growth factor (FGF) pathway inhibition to maintain naive identity. Interestingly, FGF pathway inhibition has also been suggested to cause greater loss of imprinting (LOI) (Keshet and Benvenisty, 2021). This complicates the accurate study of naive hPSCs. Furthermore, imprints and proper monoallelic gene expression do not return upon transition back to the primed state of pluripotency, a process known as “re-priming,” or subsequent differentiation (Keshet and Benvenisty, 2021; Theunissen et al., 2016). Thus, aberrant imprinting in naive hPSCs hinders developmental studies of lineage specification and the potential applications of naive hPSCs in regenerative medicine.

Several studies have surveyed the LOI found in cultured pluripotent cells by analyzing the expression of imprinted genes bearing distinguishing parental single-nucleotide polymorphisms (Bar et al., 2017; Keshet and Benvenisty, 2021; Rugg-Gunn et al., 2007). However, most studies pertaining to LOI have lacked the ability to monitor imprint integrity in live cultures. Stelzer et al. created a live-cell reporter for DNA methylation in mouse embryonic stem cells (ESCs) by utilizing a minimal promoter that is sensitive to methylation changes of adjacent sequences (Stelzer et al., 2015). However, there remains an unmet need for a reporter of imprinted gene expression in hPSCs that enables real-time visualization of LOI at single-cell resolution.

Here, we created a dual-colored fluorescent reporter at the endogenous *SNRPN* locus in primed hPSCs. We show that SNRPN acquires biallelic expression during primed-to-naive resetting, which is irreversible upon re-priming. Our reporter accurately reflects methylation at the *SNRPN* locus and is a proxy for global methylation levels. Titrating FGF pathway inhibition during naive resetting enabled us to capture a naive, imprint-protected cell population. We also demonstrate the imprint-protective effects of a KRAB zinc-finger protein, ZFP57, when ectopically expressed during the generation of naive hPSCs. When combined, these two imprint protection strategies produced an even greater imprint-protective effect. These findings provide an important step toward improving the imprint fidelity of naive hPSCs and their applications for studies of human development and regeneration.

## Results

### A live-cell reporter hPSC line displays allele-specific SNRPN expression

**Figure 1:**
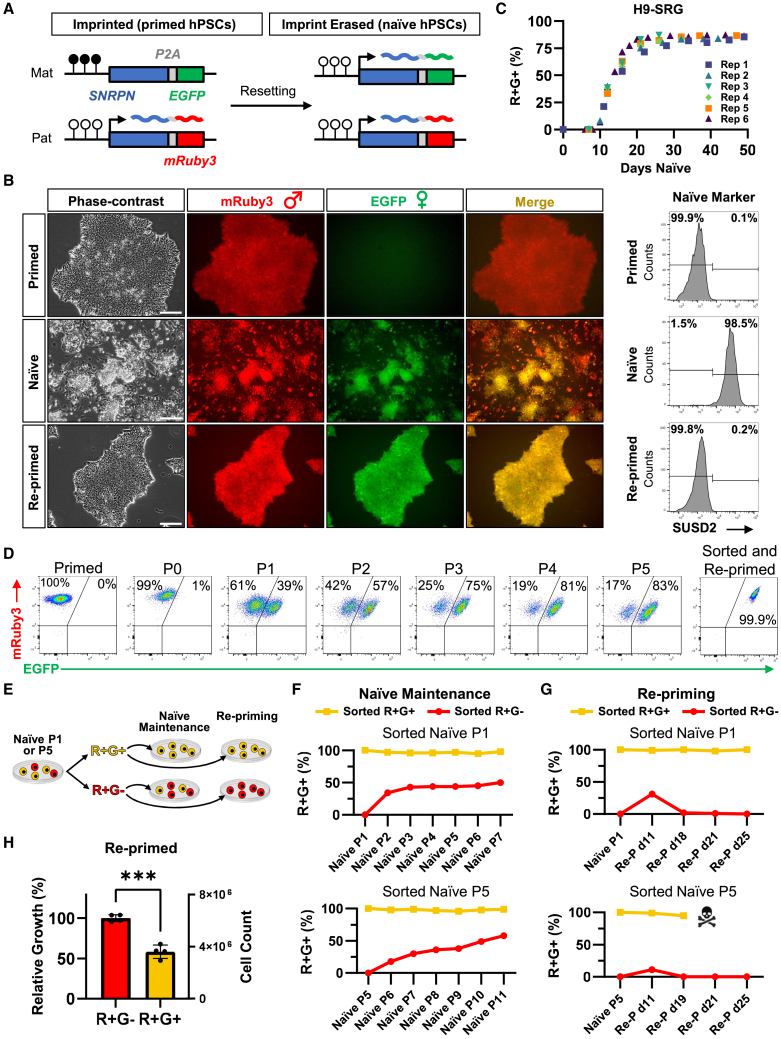
The H9-SRG dual-colored fluorescent reporter displays allele-specific *SNRPN* expression (A) Schematic of the H9-SRG reporter at the *SNRPN* locus. Closed circles indicate DNA methylation. Open circles indicate lack of DNA methylation. (B) Images and flow cytometry data for H9-SRG cells (clone 1) in the primed, naive, and re-primed states. Scale bar, 200 μm. Images are representative of four time points. See Figure S1C for clone 2. (C) Analysis of H9-SRG reporter activity during primed-to-naive resetting across six independent experiments. (D) Flow cytometry data of H9-SRG cells during primed-to-naive resetting. Plots are gated for SUSD2+ to show reporter activity of naive-converting cells (excluding primed and re-primed samples). (E) Schematic of H9-SRG naive cells sorted by reporter activity and then maintained in naive conditions or re-primed. (F) Time course of the H9-SRG reporter activity of cells sorted at naive P1 (top) or naive P5 (bottom) and maintained in naive conditions. Reporter expression was measured by flow cytometry. (G) Time course of the H9-SRG reporter activity of cells sorted at naive P1 (top) or naive P5 (bottom) and immediately re-primed. Reporter expression was measured by flow cytometry. Naive P5 sorted R+G+ cells did not survive past 19 days of re-priming. (H) Proliferation assay of re-primed R+G− and R+G+ populations that had been sorted at naive P1. Error bars represent standard deviation. ^∗∗∗^unpaired t test *p* value = 0.0001; *n* = 4 independent experiments.

To track the stability of parent-specific imprints in real time, we set out to build a dual-colored fluorescent reporter at a representative imprinted locus. We selected the *SNRPN* locus because it shows stable monoallelic expression in primed hPSCs, and the associated imprint control center (ICR) undergoes demethylation during primed-to-naive resetting, resulting in biallelic expression of the downstream *SNRPN* transcript (Pastor et al., 2016; Rugg-Gunn et al., 2007; Theunissen et al., 2016). In addition, *SNRPN* is robustly expressed in both primed and naive hPSC conditions, an important prerequisite for an endogenous live-cell reporter. We inserted a *P2A-mRuby3* sequence on one allele of *SNRPN* and a *P2A-EGFP* sequence on the other allele in H9 human embryonic stem cells (hESCs) using CRISPR-Cas9-mediated genome editing (Figures 1A and S1A). Several clones were generated, and sequence integration was validated by junction PCR (Figure S1B). We designate this genotype as H9-SNRPN-mRuby3-EGFP (H9-SRG). In the primed state, mRuby3 was highly expressed and EGFP was not expressed, suggesting that mRuby3 was integrated into the active, paternal *SNRPN* allele, while EGFP was integrated into the inactive, maternal *SNRPN* allele (Figures 1B and S1C). Consistent with LOI and a switch from mono- to biallelic *SNRPN* expression, EGFP became active in the majority of naive cells within 2–3 passages in PXGGY/A naive induction medium (Khan et al., 2021), resulting in a population of mostly double-positive mRuby3+/EGFP+ (R+G+) cells (Figures 1B and S1C). We confirmed the acquisition of naive identity in these H9-SRG cells by flow cytometry for the naive-specific cell surface marker SUSD2. Additional markers for primed and naive pluripotency were confirmed by qPCR (Figure S1D). G-banding of primed H9-SRG clones confirmed normal karyotypes (Figure S1E).

We then carefully traced the kinetics of EGFP activation across primed-to-naive resetting, which displayed high reproducibility across independent replicates (Figures 1C and 1D). The earliest we could detect EGFP expression was on day 9 of primed-to-naive resetting, with 50% EGFP activation reached around day 14. Interestingly, a small fraction of the naive population remained mRuby3+/EGFP− (R+G−), even when cultured for >5 passages. We fluorescence-activated cell sorting (FACS)-purified the H9-SRG cells undergoing resetting at passage 1 (P1) and passage 5 (P5) into R+G− and R+G+ populations and maintained them separately in naive culture (Figures 1E and 1F). The R+G− sorted population gradually repopulated the EGFP+ population, while the R+G+ sorted population remained fully EGFP+. These observations suggest that naive cells are continuously subject to imprint erasure under self-renewing naive conditions and that biallelic *SNRPN* expression is irreversible once imprinting at the maternal allele has been erased.

We next asked whether biallelic *SNRPN* expression was reversible upon re-exposure to primed culture conditions, which causes a return to an early post-implantation-like pluripotent state (An et al., 2020; Theunissen et al., 2016). Cells were FACS-purified at P1 and P5 of primed-to-naive resetting into R+G−/R+G+ populations and immediately re-primed (Figure 1G). Over time, we observed that the sorted R+G+ cells maintained high EGFP percentages, while R+G− cells maintained their EGFP− status throughout re-priming. However, we also observed a pronounced growth disadvantage of the R+G+ cells during re-priming compared to their R+G− counterparts. In fact, the EGFP+ cells that were sorted at P5 of primed-to-naive resetting could not be maintained for more than 19 days in a primed medium. To explore this growth difference further, we performed a proliferation assay on the two re-primed populations sorted from naive P1. The biallelic R+G+ population grew at 58% of the rate of the monoallelic R+G− population (Figure 1H). We then asked whether this growth difference could be due to cell-cycle differences. Based on cell-cycle analysis by flow cytometry, we observed no significant difference in the proportions of cells in G0/G1 phase, S phase, or G2 phase between R+G− and R+G+ populations (Figure S1F). Overall, these data suggest that the transition to a stable post-implantation identity is impeded in naive cells displaying biallelic SNRPN expression and that imprinted SNRPN expression can be preserved by returning EGFP− naive cells to primed culture conditions.

### SNRPN reporter activity correlates with methylation at the *SNURF* locus and is a proxy for global methylation levels

**Figure 2:**
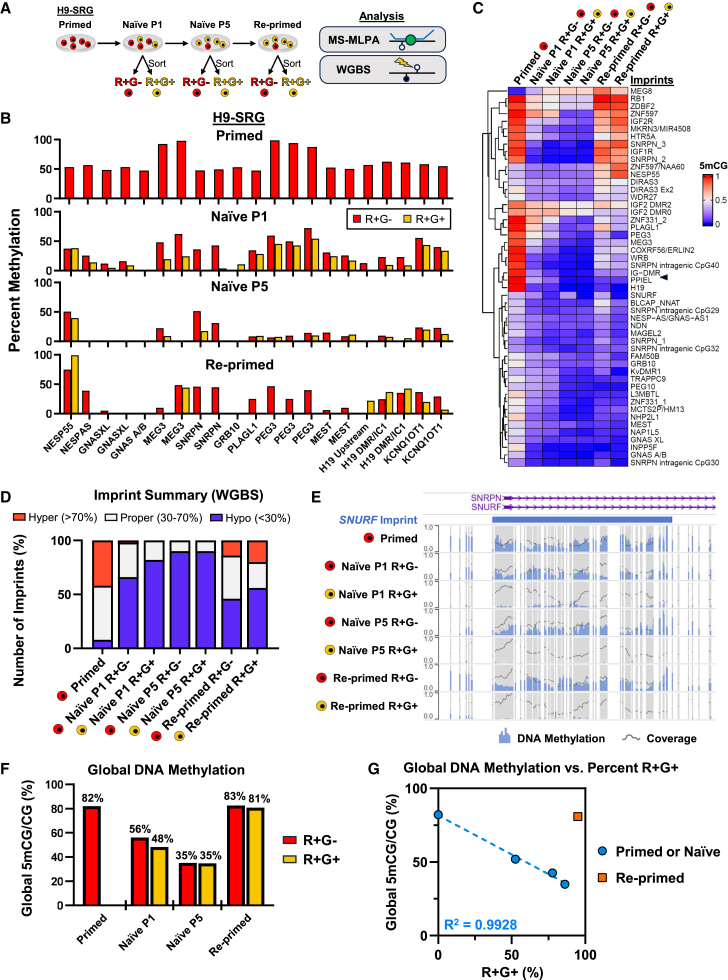
H9-SRG reporter activity corresponds to methylation at the *SNURF* locus and is a proxy for global methylation levels (A) Schematic of H9-SRG primed-to-naive resetting followed by re-priming. Cells were sorted by reporter activity at naive P1, naive P5, and re-primed time points and analyzed by MS-MLPA and WGBS. (B) MS-MLPA analysis of DNA methylation at imprints. Loci are limited to those captured in the assay. (C) WGBS analysis of DNA methylation at imprinted DMRs. (D) Summary of hyper-, hypo-, and properly methylated imprints based on WGBS analysis. (E) Browser tracks of DNA methylation at the *SNURF* locus. Vertical gray bars represent locations of CpG dinucleotides. (F) Global DNA methylation levels as measured by WGBS. (G) Analysis of global DNA methylation compared to H9-SRG reporter activity. The R^2^ value was calculated using a linear regression model on primed and naive data points.

To assess the correlation between mRuby3/EGFP expression and DNA methylation at imprinted loci, we FACS-purified R+G−/R+G+ cells at naive P1 and naive P5 and after re-priming and then performed imprint analysis by methylation-specific multiplex ligation-dependent probe amplification (MS-MLPA) (Nygren et al., 2005) and whole-genome bisulfite sequencing (WGBS) (Figure 2A). MS-MLPA analysis indicated that methylation at imprinted loci was slightly higher in R+G− cells than R+G+ cells at P1, but this difference was diminished by P5 (Figure 2B). Re-primed R+G− cells also displayed increased methylation at a subset of imprints compared to their R+G+ counterparts. WGBS analysis provided a more thorough examination of methylation. A comparison of imprinting regions, as previously defined (Court et al., 2014), revealed several key findings (Figures 2C and 2D). First, while primed hESCs have several “properly” imprinted regions (30%–70% methylation), they also possess a substantial number of hypermethylated ICRs (>70% methylation). At naive P1 and P5, imprints were largely hypomethylated (<30% methylation) in both R+G− and R+G+ samples. However, methylation was slightly elevated at imprinted loci in R+G− compared to R+G+ cells at P1 of primed-to-naive resetting. Consistent with the MS-MLPA data, this difference largely disappeared by P5. Upon re-priming, a subset of hypomethylated imprints regained significant methylation, but most remained hypomethylated, in accordance with prior observations that imprint erasure is irreversible (Pastor et al., 2016; Theunissen et al., 2016). While re-primed R+G− cells displayed elevated methylation at several imprints compared to re-primed R+G+ cells, the only ICR that consistently showed higher methylation in R+G− compared to R+G+ samples at all examined time points was *SNURF*, which is the ICR located most closely to the *SNRPN* transcriptional start site (Figure 2E). We conclude that SNRPN reporter activity correlates most strongly with methylation of the nearest differentially methylated region (DMR). Like the majority of imprinted loci, the *SNURF* DMR undergoes DNA demethylation within 5 passages of primed-to-naive resetting. However, a subset of naive cells (ca. 15%) retain monoallelic methylation of *SNURF*, but not other imprinted loci, which suggests that this ICR may be subject to unique regulatory mechanisms.

Additionally, we examined to what extent SNRPN reporter activity correlates with global 5-methylcytosine followed by guanine (5mCG) levels. Global 5mCG levels were about 80% in the primed and re-primed states and about 48%–56% and 35% at P1 and P5 of primed-to-naive resetting, respectively (Figure 2F). The elevated 5mCG level in the primed state and reduced 5mCG level in the naive state are consistent with DNA methylation patterns in human post-implantation and pre-implantation embryos, respectively (Guo et al., 2014; Zhu et al., 2018). Global 5mCG levels were elevated by 8% in R+G− compared to R+G+ cells at P1, but this difference decreased in naive P5 and re-primed samples. When we plotted global 5mCG levels relative to the proportion of R+G+ cells, we detected a strong correlation in primed and naive samples (R^2^ = 0.9928) (Figure 2G). As expected, the correlation was lost in re-primed cells since the reporter did not revert to silencing EGFP once activated. One could therefore use the R+G+ percentage to infer relative global methylation levels in primed and naive states. However, additional time points will need to be examined to discern the exact global methylation level during the early resetting process. In addition, these data are consistent with the notion that imprint erasure under naive conditions is a consequence of global DNA demethylation.

Finally, we examined global gene expression in R+G− vs. R+G+ naive cells through RNA sequencing (RNA-seq) on FACS-purified populations. There were no significant differentially expressed genes (DEGs) between the two groups at P1 of naive resetting and only two DEGs at P5: *FOS* and *FOSB* (Figure S2A). The FOS family of proteins are components of the activating protein 1 (AP-1) complex and have numerous functions in cell proliferation, survival, differentiation, and cancerous transformation (Hess et al., 2004). We closely examined the expression of markers of primed and naive pluripotency as well as epigenetic regulators, their complexes, targets, and products (collectively known as “epifactors”) (Medvedeva et al., 2015) (Figure S2B). Primed and naive samples showed robust expression of pluripotent-state-specific marker genes. However, there were no significant differences in the expression levels of epifactors between R+G− and R+G+ naive samples.

### Modulating naive culture conditions to enhance imprint stability

**Figure 3:**
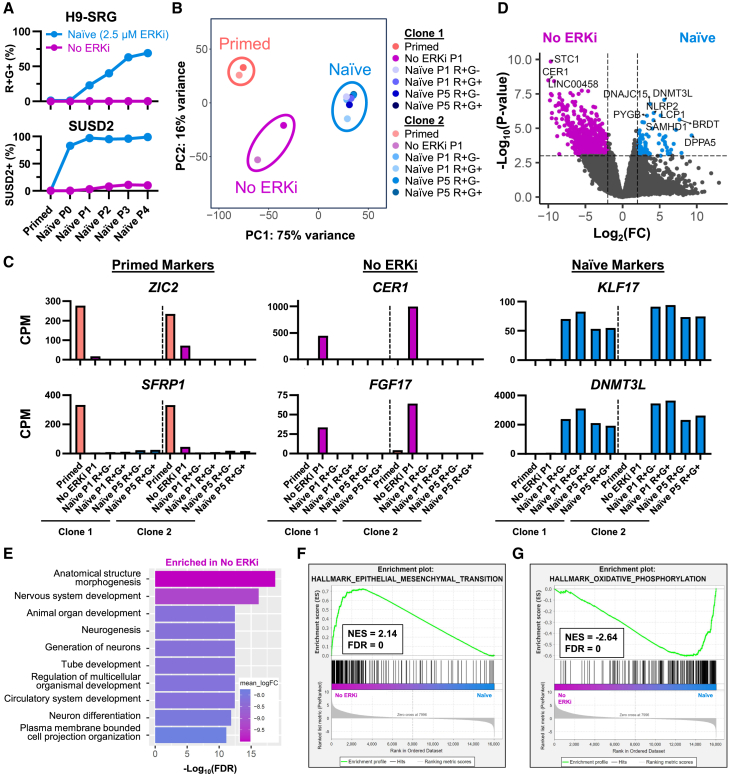
Absence of ERKi during primed-to-naive resetting precludes acquisition of *bona fide* naive identity (A) Analysis of H9-SRG reporter activity and SUSD2 expression by flow cytometry during primed-to-naive resetting using PXGGY/A or upon omission of the ERK inhibitor GDC-0994 (No ERKi). (B) Principal-component analysis of RNA-seq data from primed, no ERKi, and naive (PXGGY/A) samples using two independent clones. (C) Marker gene expression of primed, no ERKi, and naive (PXGGY/A) samples by RNA-seq using two independent clones. (D) Volcano plot of DEGs between no ERKi and naive (PXGGY/A) samples at P1 of naive resetting; *n* = 2 independent clones. (E) Gene ontology analysis of the top ten GO Biological Process terms differentially expressed between no ERKi and naive (PXGGY/A) P1 samples using two independent clones. (F) Gene set enrichment analysis of the top Hallmark signature (epithelial-mesenchymal transition) enriched in no ERKi compared to naive (PXGGY/A) using two independent clones. NES, normalized enrichment score. (G) Gene set enrichment analysis of the top Hallmark signature (oxidative phosphorylation) enriched in naive (PXGGY/A) compared to no ERKi using two independent clones. NES, normalized enrichment score.

Due to the current requirement for FGF pathway inhibition during primed-to-naive resetting (Khan et al., 2021), we sought to use our biallelic SNRPN reporter to assess the effect of reduced FGF pathway inhibition on naive identity and imprint stability. We first observed that removal of the ERK1/2 inhibitor (ERKi) GDC-0994 (hereafter called GDC) from the PXGGY/A cocktail prevented activation of the maternally methylated *SNRPN-EGFP* allele during resetting but also impaired activation of the naive marker SUSD2 (Figure 3A). We characterized these “no ERKi” cells by RNA-seq and found that they clustered between primed and naive cells by principal-component analysis (Figure 3B). Neither primed-specific markers (*ZIC2* and *SFRP1*) nor naive-specific markers (*KLF17* and *DNMT3L*) were upregulated in the no ERKi cells, although genes associated with gastrulation, such as *CER1* and *FGF17*, were upregulated only in no ERKi cells (Figure 3C). DEG analysis between no ERKi and naive PXGGY/A cells revealed a striking upregulation of genes related to neural development in no ERKi cells (Figures 3D and 3E). Gene set enrichment analysis highlighted relative enrichments for “epithelial-mesenchymal transition” in no ERKi cells and for “oxidative phosphorylation” in naive cells (Figures 3F and 3G). Since naive cells show morphological changes and increased oxygen consumption as they reset from the primed state (Dong et al., 2019; Gu et al., 2016), these results support the notion that no ERKi cells represent a cellular state between primed and naive pluripotency. These data indicate that removal of ERK inhibition prevents LOI but also fails to induce a *bona fide* naive pluripotent identity.

**Figure 4:**
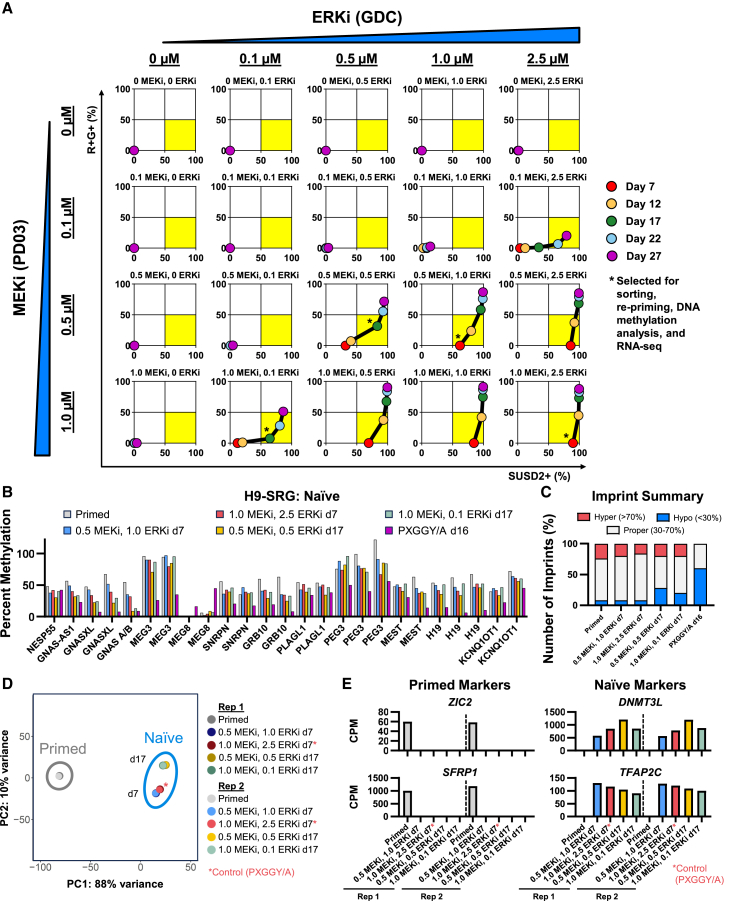
Modulating FGF signaling to enhance imprint fidelity in naive hPSCs (A) Analysis of H9-SRG reporter activity and SUSD2 expression by flow cytometry during primed-to-naive resetting using titrated amounts of MEKi (PD03) and ERKi (GDC). The lower right quadrant of each plot (yellow highlight) represents cells expressing the naive marker SUSD2 and predominantly maintaining monoallelic expression of SNRPN. (B) MS-MLPA analysis of DNA methylation at imprinted DMRs in MEKi/ERKi titrated naive samples. Naive samples are compared to the primed sample from Figure 2. MEKi/ERKi titrated naive samples were sorted for R+G-/SUSD2+ expression. (C) Summary of hyper-, hypo-, and properly methylated imprints based on MS-MLPA analysis. (D) Principal-component analysis of RNA-seq data from primed and naive-titrated samples using two replicates. MEKi/ERKi titrated naive samples were sorted for R+G-/SUSD2+ expression. (E) Marker gene expression of H9-SRG primed and naive samples by RNA-seq using two replicates.

We then asked whether the onset of biallelic SNRPN reporter activity could be delayed by reducing the concentrations of FGF pathway inhibitors. To this end, we performed a dual titration of the MEK1/2 inhibitor (MEKi) PD0325901 (hereafter called PD03) and GDC during primed-to-naive resetting using our H9-SRG imprint reporter line. We tested concentrations of 0–1 μm MEKi (PD03) and 0–2.5 μm ERKi (GDC) and tracked reporter activity as well as SUSD2 expression for up to 27 days of naive resetting (Figures 4A and S3A). Overall, reduced MEK/ERK inhibition resulted in reduced activation of the maternally methylated *SNRPN-EGFP* reporter allele but also slower SUSD2 induction. We were particularly interested in identifying conditions that support the induction of the naive marker SUSD2 without concomitant activation of the *SNRPN*-EGFP reporter allele. Based on these data, we identified several samples that were simultaneously R+G− and SUSD2+, the phenotype of interest (quadrant highlighted in Figure 4A). Greater MEK/ERK inhibition caused cells to enter this imprinted-protected naive state earlier (day 7–12), but these conditions invariably transitioned to the R+G+ state at later time points. In contrast, lower MEK/ERK inhibition led to a slower, but more sustained, R+G−/SUSD2+ state.

We then explored to what extent R+G−/SUSD2+ cells under these modified conditions in fact maintained imprint methylation and possessed naive identity. We FACS-purified the samples “0.5 MEKi, 1.0 ERKi” and “1.0 MEKi, 2.5 ERKi” at day 7 and samples “0.5 MEKi, 0.5 ERKi” and “1.0 MEKi, 0.1 ERKi” at day 17 for R+G−/SUSD2+ markers. We then performed MS-MLPA and RNA-seq analysis to assess imprint methylation and naive identity. MS-MLPA analysis revealed that titrated naive samples maintained imprinted methylation better than the naive controls at similar time points (Figures 4B and 4C). Analysis of RNA-seq results indicated that all titrated samples reached the naive state as shown by principal-component analysis and marker gene expression (Figures 4D and 4E). Thus, these findings indicate that it is possible to acquire a *bona fide* naive identity in which imprints are retained under modified culture conditions, but this phenotype persists only transiently and in a subset of naive cells.

To test the effect of a short naive pulse followed by continued primed culture on imprint stability, we re-primed the sorted R+G−/SUSD2+ cells. WGBS analysis of the re-primed naive-titrated samples showed retention of methylation at imprints (Figure S3B). Additionally, for a subset of imprints, methylation shifted closer to 50% in cells that were re-primed after a brief naive pulse. Although subtle, there were improvements in the numbers of 30%–70% methylated loci and decreases in the numbers of hyper- and hypomethylated loci after re-priming. As an important caveat, however, these samples were not tested for allele-specific methylation. Therefore, further work will be required to determine whether transient naive treatment followed by re-priming may in fact modestly improve the fraction of imprints with the correct monoallelic methylation pattern.

### Enhancing imprint stability by overexpressing candidate imprint-protecting factors

**Figure 5:**
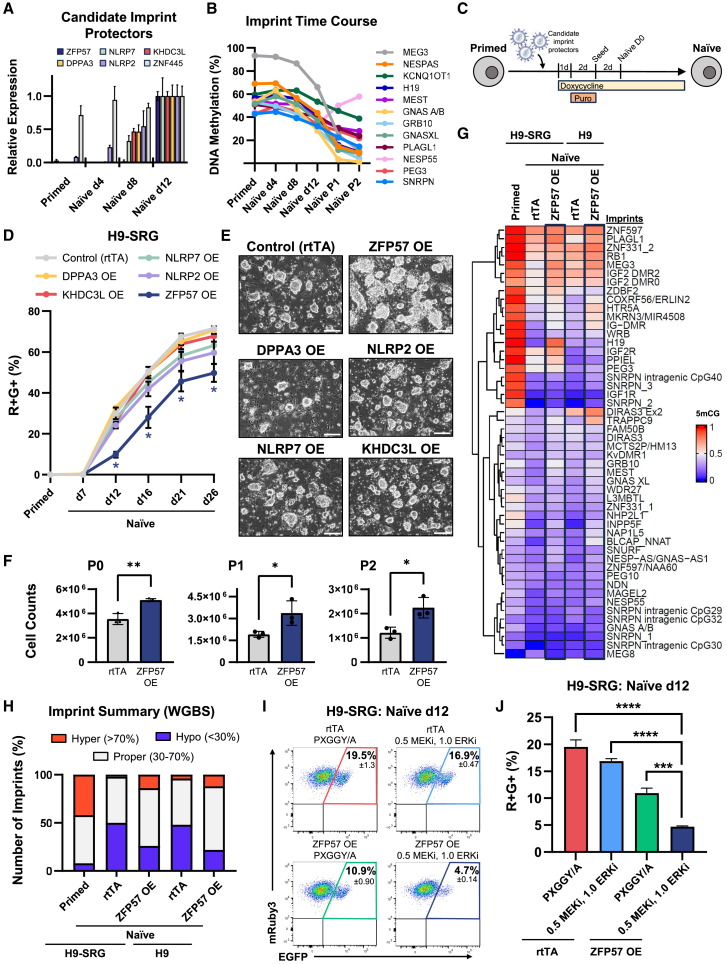
Overexpression of candidate imprint-protecting factors during primed-to-naive resetting (A) Gene expression analysis of candidate factors in H9 hESCs by qPCR; *n* = 2 or 3 independent experiments. (B) MS-MLPA analysis of DNA methylation at imprints during H9 primed-to-naive resetting. Imprints with multiple data points from the same locus were averaged for simplicity. (C) Schematic for lentiviral transduction of candidate imprint-protecting factors in hPSCs. Doxycycline was added to activate transgene overexpression and puromycin resistance, and then cells were puromycin-selected for two days. Puromycin was removed, and cells were reset to the naive state under continued doxycycline treatment. (D) Analysis of H9-SRG reporter activity by flow cytometry during primed-to-naive resetting of genetic overexpression samples. Error bars represent the standard error of the mean; *n* = 3 independent experiments. Significance comparisons reflect ZFP57 OE vs. control (rtTA). ^∗^Multiple unpaired t test q value < 0.05. (E) Images of H9 naive cells overexpressing the indicated transgene. Scale bar, 200 μm. (F) Cell counts of H9 rtTA and ZFP57 OE samples at the end of each passage during primed-to-naive resetting and maintenance. Samples were seeded at 250k cells/well and split at identical ratios after each passage. Error bars represent standard deviation; *n* = 3 independent experiments. ^∗^Unpaired t test *p* value < 0.05. ^∗∗^unpaired t test *p* value < 0.005. (G) WGBS analysis of imprints in control and ZFP57 overexpression samples during naive resetting in PXGGY/A (H9-SRG, day 12) and 5i/L/A (H9, day 16). Naive samples were compared to the primed sample from Figure 2. (H) Summary of hyper-, hypo-, and properly methylated imprints based on WGBS analysis. (I) Flow cytometry data of H9-SRG cells with rtTA and/or ZFP57 transgenes at day 12 of primed-to-naive resetting in PXGGY/A or “0.5 MEKi, 1.0 ERKi” conditions. Plots are gated for SUSD2+ to show reporter activity of naive-converting cells. (J) Analysis of the H9-SRG R+G+ population in Figure 5I. Error bars represent the standard deviation; *n* = 3 independent experiments. ^∗∗∗^Unpaired t test *p* value < 0.0005. ^∗∗∗∗^Unpaired t test *p* value < 0.0001.

As an alternative approach, we investigated whether imprint stability could be enhanced by ectopic expression of candidate imprint-protecting factors. Studies in mice and humans have identified Dppa3/DPPA3, Zfp57/ZFP57, and Zfp445/ZNF445 as imprint-protecting factors during post-fertilization epigenetic reprogramming (Li et al., 2008; Mackay et al., 2008; Nakamura et al., 2007; Takahashi et al., 2019). Additionally, mutations in various components of the maternally deposited subcortical maternal complex, including *NLRP2*, *NLRP7*, and *KHDC3L*, are associated with molar pregnancy and widespread multi-locus imprinting disturbances (Monk et al., 2017). We asked whether these factors were present in our primed and naive hPSCs. qPCR analysis showed that all of these factors, except *ZNF445*, were lowly expressed in the primed state but were significantly upregulated in the naive state (Figure 5A). In contrast, *ZNF445* was robustly expressed throughout the primed-to-naive transition. Interestingly, however, we noticed that the expression of the other candidate imprint-protecting factors did not increase until day 8–12 of naive resetting. MS-MLPA analysis indicated that imprint methylation had already started to decrease during day 8–12, while some imprints exhibited demethylation at even earlier time points (Figure 5B). Based on these data, we hypothesized that candidate imprint-protecting factors (e.g., ZFP57, DPPA3, NLRP2, NLRP7, or KHDC3L) must be expressed from the beginning of primed-to-naive resetting in order to protect imprint methylation.

To examine this hypothesis, we used a doxycycline-inducible system to overexpress each factor individually in our H9-SRG imprint reporter line at the start of primed-to-naive resetting (Figure 5C). Successful overexpression of all transgenes was confirmed by qPCR (Figure S4A). While the level of transgenic ZFP57 appeared to be higher than that of the other overexpression factors, the endogenous levels of the other factors in naive hESCs were notably higher than that of ZFP57 (Figure S4B), thereby inflating the relative expression level of ZFP57 transgene in the primed state. During resetting, ZFP57 overexpression led to delayed onset of biallelic SNRPN expression (Figure 5D), while overexpression of DPPA3, NLRP2, NLRP7, or KHDC3L did not have a significant effect. The vast majority of cells showed high SUSD2 surface marker expression in the naive state, suggesting that overexpression of candidate imprint-protecting factors did not compromise the acquisition of naive identity (Figure S4C). We then analyzed methylation levels at imprinted genes by MS-MLPA at days 12 and 16 of naive resetting. Consistent with delayed activation of the maternally imprinted SNRPN-EGFP allele, ZFP57 overexpression protected methylation levels at a subset of imprints (Figure S4D). However, methylation protection was not seen upon overexpression of the other candidate factors. We repeated this experiment using wild-type H9 hESCs (that do not contain the imprint reporter) using an alternative naive induction medium, 5i/L/A (Theunissen et al., 2014, 2016). Again, we observed a moderate protective effect of ZFP57 overexpression on imprint methylation (Figure S4D). These results demonstrate that the protective effect of ZFP57 is not limited to a specific culture system for inducing naive human pluripotency. Interestingly, the overexpression of ZFP57 also conferred a notable proliferation and/or survival advantage to naive cells derived in 5i/L/A (Figure 5E). Cell counts at three time points during naive resetting and maintenance showed a significant difference between rtTA and ZFP57 overexpression (OE) samples (Figure 5F). This effect may have been masked by the higher conversion efficiency in PXGGY/A (Khan et al., 2021).

To explore the imprint-protective effect of ZFP57 in more detail, we performed WGBS on H9-SRG control and ZFP57 overexpression samples at naive day 12 and day 16, as well as H9 control and ZFP57 overexpression at naive day 16. Nearly all imprints showed reduced methylation levels in naive control samples compared to the primed state (Figure 5G). Importantly, methylation levels were notably higher in ZFP57 overexpression samples at many imprints compared to the naive controls. Analysis of hyper-, hypo-, and properly methylated imprints indicated a substantial reduction in the number of hypomethylated imprints in naive ZFP57 overexpression compared to naive control samples (Figure 5H). Likewise, the number of properly methylated imprints increased with ZFP57 overexpression during primed-to-naive resetting. Substantial global DNA demethylation occurred in all naive samples compared to the primed state (Figure S4E). Global methylation levels were similar, although slightly elevated, in naive ZFP57 overexpression samples compared to naive controls. Importantly, however, ZFP57 upregulation did not affect the acquisition of naive pluripotency, as assessed by RNA-seq (Figure S4F). Principal-component analysis primarily separated between primed and naive states. While there was a small separation between the PXGGY/A and 5i/L/A samples, in accordance with our previous observations (Khan et al., 2021), rtTA and ZFP57 samples in the same conditions clustered together closely. We conclude that ZFP57 overexpression during primed-to-naive resetting provided imprint protection, potentially as a result of slower DNA demethylation, but did not interfere with naive identity.

We then asked whether combining the two imprint protection strategies (ZFP57 overexpression and MEKi/ERKi titration) would yield a greater effect than either strategy alone. Using our H9-SRG reporter line with and without ZFP57 overexpression, we tracked biallelic SNRPN activation during naive resetting in normal PXGGY/A medium and the titrated “0.5 MEKi, 1.0 ERKi” condition. Each strategy alone reduced biallelic SNRPN expression at day 12 of resetting, with an even greater decrease when both strategies were used together (Figures 5I and 5J). The naive marker SUSD2 was highly expressed in all samples, indicating that these imprint-protective strategies did not interfere with the acquisition of naive identity (Figure S4G).

Finally, based on the results obtained with ZFP57 overexpression, we considered whether other KZFP family genes might exhibit similar expression kinetics during primed-to-naive resetting and may therefore have a similar imprint-protective effect. To this end, we examined 25 KZFPs identified by the Monk group as binding factors to more than one imprinted region (Monteagudo-Sánchez et al., 2020). Using RNA-seq analysis of primed-to-naive intermediate cell populations reported by the Rugg-Gunn group (Collier et al., 2017), we identified three additional KZFPs (ZNF257, ZNF506, and ZNF534) that were significantly upregulated in naive compared to primed and intermediate cell populations (Figure S4H). Consistent with our data, ZFP57 also showed significant upregulation in the naive state, while ZNF445 showed consistent expression across primed, intermediate, and naive cell populations. The KZFPs ZNF257, ZNF506, and ZNF534 could be promising candidates for further investigation as candidate imprint-protecting factors to be expressed continuously during primed-to-naive resetting.

## Discussion

In the 10 years since naive hPSCs were first derived, they have become widely adopted in stem cell research, facilitating studies into basic mechanisms of early human development, the derivation of extraembryonic cell types, and the generation of stem-cell-based embryo models (reviewed in Dong et al., 2019; Zhou et al., 2023). However, a persistent issue hampering the application of naive hPSCs is the erosion of parent-specific imprints under currently available culture regimes. In this study, we established a dual-colored fluorescent reporter cell line at the *SNRPN* locus (H9-SRG) to track the dynamics of imprint erasure during naive resetting at single-cell resolution. We showed that this reporter accurately reflects methylation at the *SNURF* ICR and correlates with 5mCG methylation globally. Using our H9-SRG imprint reporter as a cellular sensor for methylation levels, we evaluated two independent strategies to mitigate the LOI in naive hPSCs: titrating the concentrations of key kinase inhibitors or overexpressing candidate imprint-protecting factors. Our findings demonstrate that reducing the levels of MEK/ERK inhibition or overexpressing the KRAB zinc-finger protein ZFP57 can protect a subset of imprints during primed-to-naive resetting. Furthermore, combined MEK/ERK inhibitor titration and ZFP57 overexpression confer greater imprint protection than either strategy alone.

An important question when evaluating a live-cell reporter is to what extent the activity recorded from a single locus is representative of the genome-wide process of interest (in this case, imprinting). We opted to build an imprint reporter in the *SNRPN* locus because its ICR is known to undergo demethylation during primed-to-naive resetting, while the *SNRPN* transcript is robustly expressed across human pluripotent states (Pastor et al., 2016; Theunissen et al., 2016). Consistent with imprint erasure and a switch from mono- to biallelic SNRPN expression, the maternal *SNRPN-EGFP* allele became active in the majority of cells within 2–3 passages of naive resetting. As expected, these double-positive H9-SRG R+G+ cells displayed extensive DNA demethylation at the *SNRPN* locus and other imprinted loci. However, a subpopulation of naive cells retained monoallelic SNRPN expression (H9-SRG R+G−) and exhibited elevated DNA methylation at several ICRs at an early stage of primed-to-naive resetting (P1), but not at later passages (P5). The only ICR that consistently displayed elevated DNA methylation in H9-SRG R+G− naive cells was *SNURF*, which suggests that SNRPN reporter activity correlates most strongly with methylation of its nearest ICR. The H9-SRG reporter followed the genome-wide trend of ICR demethylation in the vast majority of cells (∼85% by P5 of naive resetting) and strongly correlated with global DNA methylation levels. Nevertheless, the H9-SRG reporter only shows partial correlation with other imprints, and the creation of an additional reporter, for example, one that targets a paternally imprinted locus, may allow for more sensitive detection of global LOI in the future.

We utilized the H9-SRG reporter to evaluate two independent strategies to preserve imprint integrity during primed-to-naive resetting. Based on our observation that omission of the ERK inhibitor GDC from the PXGGY/A cocktail causes primed hPSCs to enter an imprint-protected intermediate state, we assessed the impact of titrated levels of MEK and ERK inhibitors. Our results demonstrated that reducing MEK/ERK inhibition during naive resetting allows a subset of cells to enter a *bona fide* naive state with intact imprints. This population can be enriched using our H9-SRG reporter and sorting for R+G−/SUSD2+ cells. A small reduction in MEK/ERK inhibition supports an imprint-protected naive state by day 7 of naive treatment, but these cells have reduced imprints by day 12–17. However, a greater reduction of MEK/ERK inhibition supports a slower-to-attain but more sustained imprint-protected naive state. Both methods may be useful to the field depending on the experimental scenario. This approach is conceptually similar to that of Di Stefano and colleagues, who applied a reduced concentration of MEK inhibition to enhance genome stability during primed-to-naive resetting (Di Stefano et al., 2018).

As an alternative approach, we examined whether imprint stability could be enhanced by overexpressing candidate imprint-protecting factors during primed-to-naive resetting. Many biochemical factors appear to be important for imprint stability in humans (Monk et al., 2017; Monteagudo-Sánchez et al., 2020; Takahashi et al., 2019). Here, we demonstrated that, while several candidate imprint-protecting proteins may be important during early embryogenesis, of those tested, only overexpression of ZFP57 could protect a subset of imprints during primed-to-naive resetting. These results were reproduced using two independent naive induction cocktails, PXGGY/A (Khan et al., 2021) and 5i/L/A (Theunissen et al., 2014). In case of the latter, ZFP57 also conferred a growth/survival advantage during naive resetting. This method of using a transgene to help protect imprinting is a significant step toward more complete imprint protection. We postulate that a transgenic approach may allow for further elucidation of imprint-protective mechanisms and facilitate the development of non-transgenic methods to enhance the epigenetic stability of naive hPSCs.

Landmark studies in mouse ESCs demonstrated that germline transmission is required for establishment of monoallelic methylation and expression patterns of imprinted genes (Tucker et al., 1996). Consistent with these observations, we and others previously reported that imprints do not recover upon differentiation of naive hPSCs (Pastor et al., 2016; Theunissen et al., 2016). We FACS-purified H9-SRG cells at P1 and P5 of primed-to-naive resetting into R+G−/R+G+ populations and immediately returned them to primed culture conditions. While the sorted R+G+ cells maintained high EGFP percentages, R+G− cells maintained their EGFP− status throughout re-priming. These data confirm that imprint erasure is irreversible during primed-to-naive resetting and that imprinted *SNRPN* expression can be preserved by returning EGFP− naive cells to primed culture conditions before LOI has occurred. Intriguingly, R+G− cells that were maintained for 5 passages in naive media and then returned to primed conditions also exhibited enhanced DNA methylation at several ICRs outside the *SNRPN* locus, which raises the possibility that some parent-specific memory may be retained independently of DNA methylation.

While most imprints are properly methylated in the primed state, our results indicate that there are numerous exceptions where imprints are hyper- or hypomethylated in primed hPSCs. Similar to the recent work by Buckberry et al., we asked whether a short pulse of naive treatment could improve the epigenetic state of primed hPSCs (Buckberry et al., 2023). Our re-priming of H9-SRG reporter cells following a short naive treatment with reduced MEK/ERK inhibition showed that methylation levels at imprints can be retained when not erased in the naive state. However, allele-specific methylation data will be needed to determine the full extent of imprint preservation and whether transient naive treatment followed by re-priming may in fact lead to a modest improvement in the fraction of properly imprinted imprints at the expense of hyper- and hypomethylated imprints.

Since ZFP57 is expressed in human blastocysts (Takahashi et al., 2019), one might ask whether blastocyst-derived naive hESCs retain proper imprinted methylation. Established HNES1 cells in t2iLGӧ(Y) medium were still found to have largely hypomethylated imprints (Guo et al., 2016, 2017). This suggests, in agreement with our data (Figure 5D), that naive culture conditions still erode imprints over time even in the presence of ZFP57. It will be of interest to investigate whether imprints are better preserved in blastocyst-derived naive hESCs at early passages and delineate the imprint-protective effects of ZFP57 and other factors in this context. Additional factors of interest include ZNF257, ZNF506, and ZNF534, which were identified as having imprint-binding capacity (Monteagudo-Sánchez et al., 2020) and showed similar expression dynamics as ZFP57 during primed-to-naive resetting (Collier et al., 2017). Overall, the findings in this study provide an important step toward improving the imprint fidelity of naive hPSCs and their applications for studies of human development and regeneration.

## Methods

### Ethics statement

All experiments involving hESCs were approved by the Institutional Biological and Chemical Safety Committee and Embryonic Stem Cell Research Oversight Committee at Washington University School of Medicine.

### hPSC culture

Primed hPSCs were cultured on Matrigel in mTeSR Plus media at 37°C, 20% O_2_, and 5% CO_2_. Naive hPSCs were cultured on mouse embryonic fibroblasts in their respective media at 37°C, 5% O_2_, and 5% CO_2_. Mycoplasma testing was performed routinely, and all samples were negative. Cultures were consistently monitored and were negative for bacteria and fungus. See supplemental methods for more details.

### Gene editing to establish the SNRPN dual-colored reporter

H9 primed hESCs were nucleofected with sgRNA, Cas9 protein, and donor plasmids using a Lonza 4-D nucleofector system. Single-cell clones were obtained by FACS and expanded in culture. Inserts were validated by junction PCR. See supplemental methods for more details.

### Flow cytometry/FACS

Flow cytometry was performed on a Sony SY3200 Synergy cytometer (FACS purification) or a Beckman Coulter CytoFLEX S cytometer (analysis). See supplemental methods for more details.

### MS-MLPA assay

MS-MLPA assays were performed using reagents from MRC Holland multi-locus imprinting kits ME034-B1 or ME034-C1 according to manufacturer’s instructions (Nygren et al., 2005). See supplemental methods for more details.

### RNA isolation, cDNA synthesis, and qPCR

Total RNA was isolated using the RNeasy mini kit (QIAGEN, 74104) with DNase I treatment (Omega, E1091) per manufacturer’s instructions. Total RNA was reverse-transcribed using high-capacity cDNA reverse transcription reagents (Applied Biosystems, 4368814). cDNA was diluted 1:20 in molecular-grade water. qPCR reactions were performed with PowerUp SYBR Green master mix (Applied Biosystems, A25743) on an Applied Biosystems StepOnePlus or QuantStudio 3 real-time PCR system. Oligos are listed in Table S1. Gene expression values were normalized to the housekeeping gene *RPLP0*. Error bars represent the standard deviation. Plots were visualized with GraphPad Prism 10.1.2.

### Bulk RNA-seq and analysis

Samples were sequenced on an Illumina NovaSeq 6000 or NovaSeq X Plus using paired end reads of 150 bases. Basecalls and demultiplexing were performed with Illumina’s bcl2fastq software and a custom python program with a maximum of one mismatch in the indexing read. See supplemental methods for more details.

### WGBS analysis

Bisulfite conversion was performed on genomic DNA using the EZ DNA Methylation-Gold kit (Zymo Research, D5005). Library preparation was performed manually using reagents from the Accel-NGS Methyl-Seq DNA library kit (Swift BioSciences, 30024). Libraries were sequenced on an Illumina NovaSeq 6000 (Figure 2) or NovaSeq X Plus (Figures 5 and S3) flow cell using 300 cycles. See supplemental methods for bioinformatic processing.

## Resource availability

### Lead contact

Requests for further information and resources should be directed to and will be fulfilled by the lead contact, Thorold W. Theunissen (t.theunissen@wustl.edu).

### Materials availability

Materials are available upon reasonable request to the lead contact.

### Data and code availability

Raw and processed data for WGBS and RNA-seq experiments generated in this study have been deposited in the NCBI’s Gene Expression Omnibus and are available under GSE268536 and GSE268535, respectively.

## Acknowledgments

We thank Malkiel Cohen and Rudolf Jaenisch for sharing the inducible lentiviral overexpression vector. We thank the Genome Engineering & Stem Cell Center at The McDonnell Genome Institute of Washington University for assistance with constructing CRISPR-Cas9 knockins, the Cytogenetics and Molecular Pathology Laboratory in the Department of Pathology and Immunology for G-banded karyotyping, and the Genome Technology Access Center at The McDonnell Genome Institute for assistance with bulk RNA-seq and WGBS. For scientific input and manuscript feedback, we thank John R. Edwards, Kristen L. Kroll, Tim Schedl, Eric L. Greer, and members of the Theunissen Lab.

This work was supported by the 10.13039/100000002NIH Director’s New Innovator Award (DP2GM137418); the 10.13039/100000057NIGMS Maximizing Investigator’s Research Award (R35GM153439-01); and grants from the Shipley Foundation Program for Innovation in Stem Cell Science, the Edward Mallinckrodt, Jr. Foundation Grant, and Washington University Children's Discovery Institute to T.W.T. Additional support was provided by the 10.13039/100000062NIDDK (R01DK046718) to H.J. as well as an NIH CMB Training Grant (T32GM007067) and a Douglas Covey Graduate Student Fellowship to L.A.F.

## Author contributions

L.A.F., J.E.Z., B.M., and K.-m.P. performed and assisted with experiments under the supervision of T.W.T. M.R. performed and analyzed MS-MLPA experiments under the supervision of H.J. on samples provided by L.A.F. Bulk RNA-seq and WGBS were analyzed by L.A.F. and J.K.H. under the supervision of S.D., T.W., and T.W.T. L.A.F. and T.W.T. wrote the manuscript with input from others.

## Declaration of interests

T.W.T. is a member of the Early Career Editorial Board at Stem Cell Reports and a consultant for Stately Bio, Inc. L.A.F. and T.W.T. are co-inventors on a patent application related to tracking and mitigating imprint stability in naive hPSCs.

## Supplemental information

Document S1. Figures S1–S4 and Table S1 and supplemental methods

Document S2. Article plus supplemental information
