# Supplementary material for: Tracking and mitigating imprint erasure during induction of naive human pluripotency at single-cell resolution
Source: Stem Cell Reports. 2025 Feb 13;20(3):102419. doi: 10.1016/j.stemcr.2025.102419 (PMC11960550; doi:10.1016/j.stemcr.2025.102419)
Supplement: Document S1. Figures S1–S4 and Table S1 and supplemental methods [file mmc1.pdf]

Stem Cell Reports, Volume *20*

Supplemental Information

Tracking and mitigating imprint erasure during induction of naive human pluripotency at single-cell resolution

Laura A. Fischer, Brittany Meyer, Monica Reyes, Joseph E. Zemke, Jessica K. Harrison, Kyoung-mi Park, Ting Wang, Harald Jüppner, Sabine Dietmann, and Thorold W. Theunissen

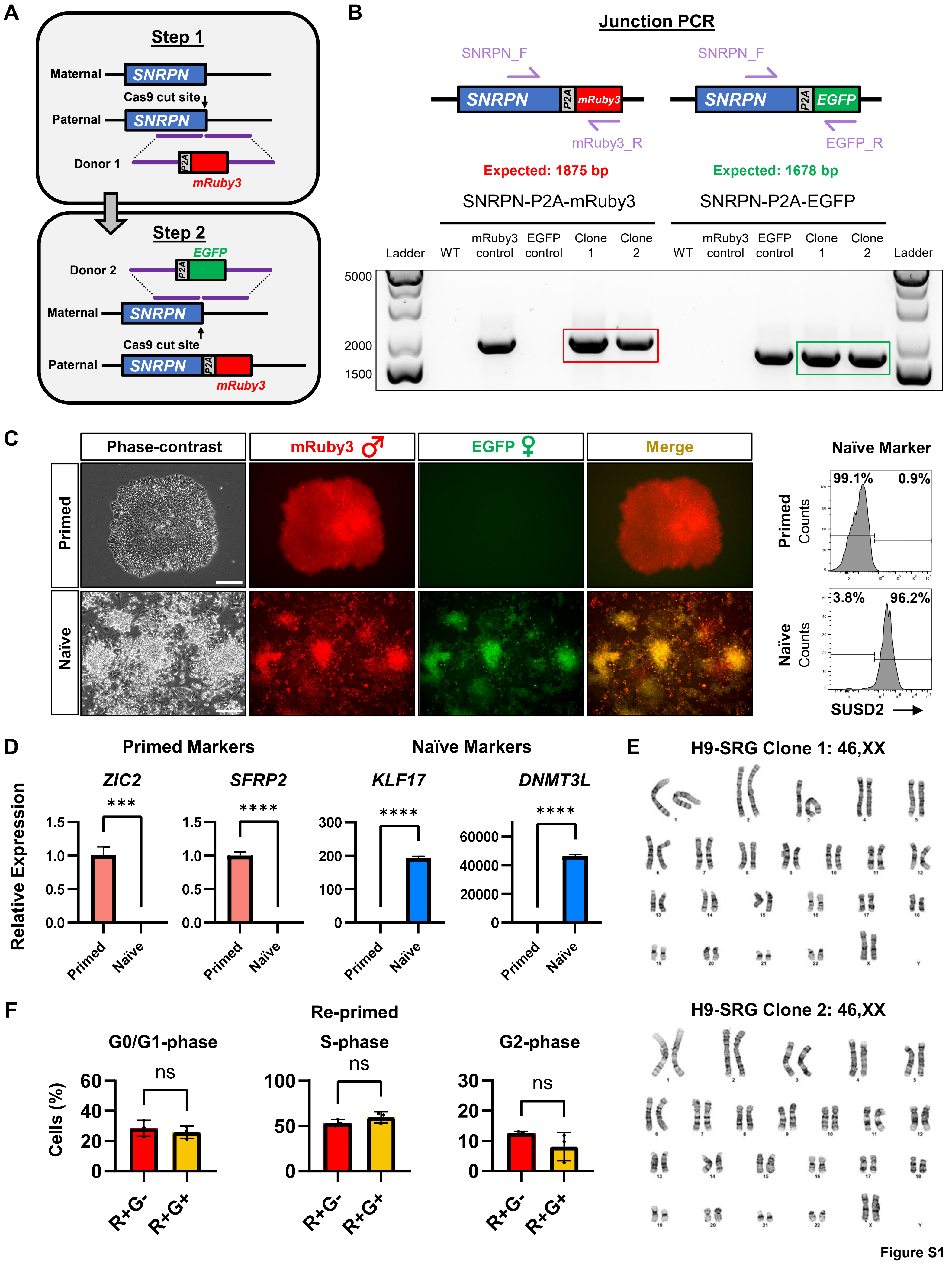
A
Step 1
Maternal
SNRPN
Cas9 cut site
Paternal
SNRPN
Donor 1
P2A
mRuby3
Step 2
Donor 2
P2A
EGFP
Maternal
SNRPN
Cas9 cut site
Paternal
SNRPN
P2A
mRuby3
B
Junction PCR
SNRPN_F
SNRPN
P2A
mRuby3
mRuby3_R
SNRPN_F
SNRPN
P2A
EGFP
EGFP_R
Expected: 1875 bp
Expected: 1678 bp
SNRPN-P2A-mRuby3
SNRPN-P2A-EGFP
Ladder
WT
mRuby3 control
EGFP control
Clone 1
Clone 2
WT
mRuby3 control
EGFP control
Clone 1
Clone 2
Ladder
5000
2000
1500
C
Phase-contrast
mRuby3 ♂
EGFP ♀
Merge
Primed
Naïve
Naïve Marker
Primed Counts
99.1%
0.9%
Naïve Counts
3.8%
96.2%
SUSD2
D
Primed Markers
Naïve Markers
ZIC2
SFRP2
KLF17
DNMT3L
Relative Expression
***
****
****
****
1.5
1.0
0.5
0.0
300
200
100
0
60000
40000
20000
0
Primed
Naïve
E
H9-SRG Clone 1: 46,XX
H9-SRG Clone 2: 46,XX
F
Re-primed
G0/G1-phase
S-phase
G2-phase
Cells (%)
ns
60
40
20
0
100
50
0
30
20
10
0
R+G-
R+G+
Figure S1

Figure S1: Constructing and characterizing the H9-SRG dual-colored reporter [Related to Figure 1]

- **A.** Creation of the H9-SRG reporter cell line. First, *P2A-mRuby3* was integrated by CRISPR/Cas9 editing into the active, paternal *SNRPN* allele. Single-cell clones were isolated by FACS. Second, *P2A-EGFP* was integrated by CRISPR/Cas9 editing into the silent, maternal *SNRPN* allele. Single-cell clones were isolated and later screened by junction PCR.
- **B.** Validation of *P2A-mRuby3* and *P2A-EGFP* integration at the *SNRPN* locus by junction PCR. "WT" = H9 primed. "mRuby3 control" has only mRuby3 integrated. "EGFP control" has only EGFP integrated. "Clone 1" and "Clone 2" each have an mRuby3 and an EGFP integration and were used throughout this study.
- **C.** Images and flow cytometry data for H9-SRG cells (Clone 2) in the primed and naïve states. Scale bar = 200 µm. Images are representative of four time points. See Figure 1B for Clone 1.
- **D.** Relative expression of primed and naïve marker genes in H9-SRG cells (Clone 1) by qPCR. All genes are displayed with Primed = 1.0. Error bars represent standard deviation; n = 3 independent experiments. ***unpaired t-test p-value = 0.0001. ****unpaired t-test p-value < 0.0001.
- **E.** Karyotype analysis of H9-SRG Clones 1 and 2.
- **F.** Cell cycle analysis of re-primed R+G- and R+G+ populations that had been sorted at naïve P1. Error bars represent standard deviation; n = 3 independent experiments. "ns" represents unpaired t-test p-value > 0.05.

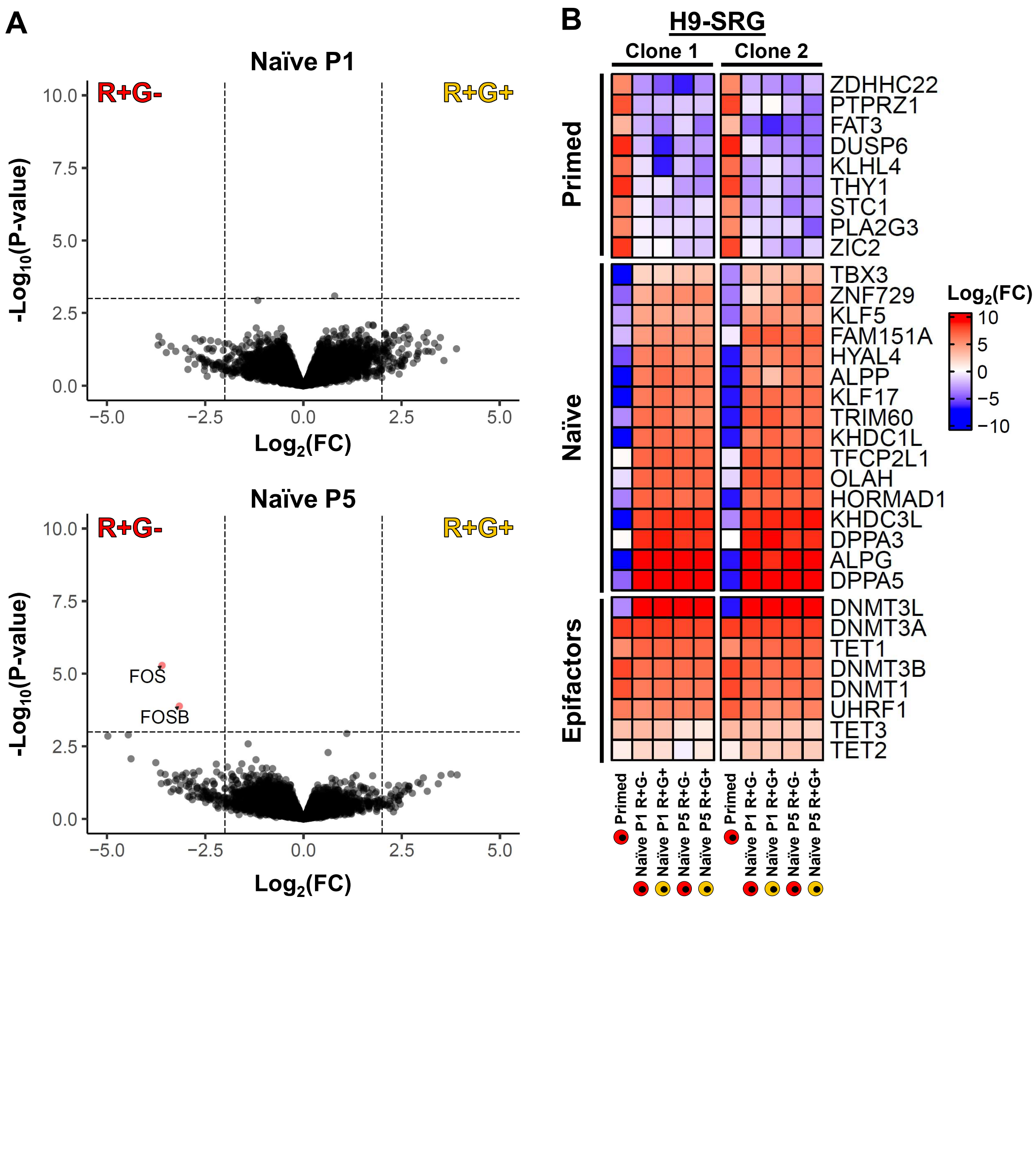
A
Naïve P1
R+G-
R+G+
-Log10(P-value)
Log2(FC)
Naïve P5
FOS
FOSB
B
H9-SRG
Clone 1
Clone 2
Primed
Naïve
Epifactors
ZDHHC22
PTPRZ1
FAT3
DUSP6
KLHL4
THY1
STC1
PLA2G3
ZIC2
TBX3
ZNF729
KLF5
FAM151A
HYAL4
ALPP
KLF17
TRIM60
KHDC1L
TFCP2L1
OLAH
HORMAD1
KHDC3L
DPPA3
ALPG
DPPA5
DNMT3L
DNMT3A
TET1
DNMT3B
DNMT1
UHRF1
TET3
TET2
Log2(FC)
Primed
Naïve P1 R+G-
Naïve P1 R+G+
Naïve P5 R+G-
Naïve P5 R+G+

Figure S2

Figure S2: RNA-seq analysis of H9-SRG cells [Related to Figure 2]

- **A.** Analysis of DEGs between sorted R+G- and R+G+ samples at naïve P1 (top) and P5 (bottom). n = 2 independent clones for each time point. Significance cutoffs were assigned as $Log_2(FC) \geq 2$ and $-Log_{10}(P\text{-value}) \geq 3$.
- **B.** Gene expression analysis of H9-SRG samples sorted by reporter activity using two independent clones.

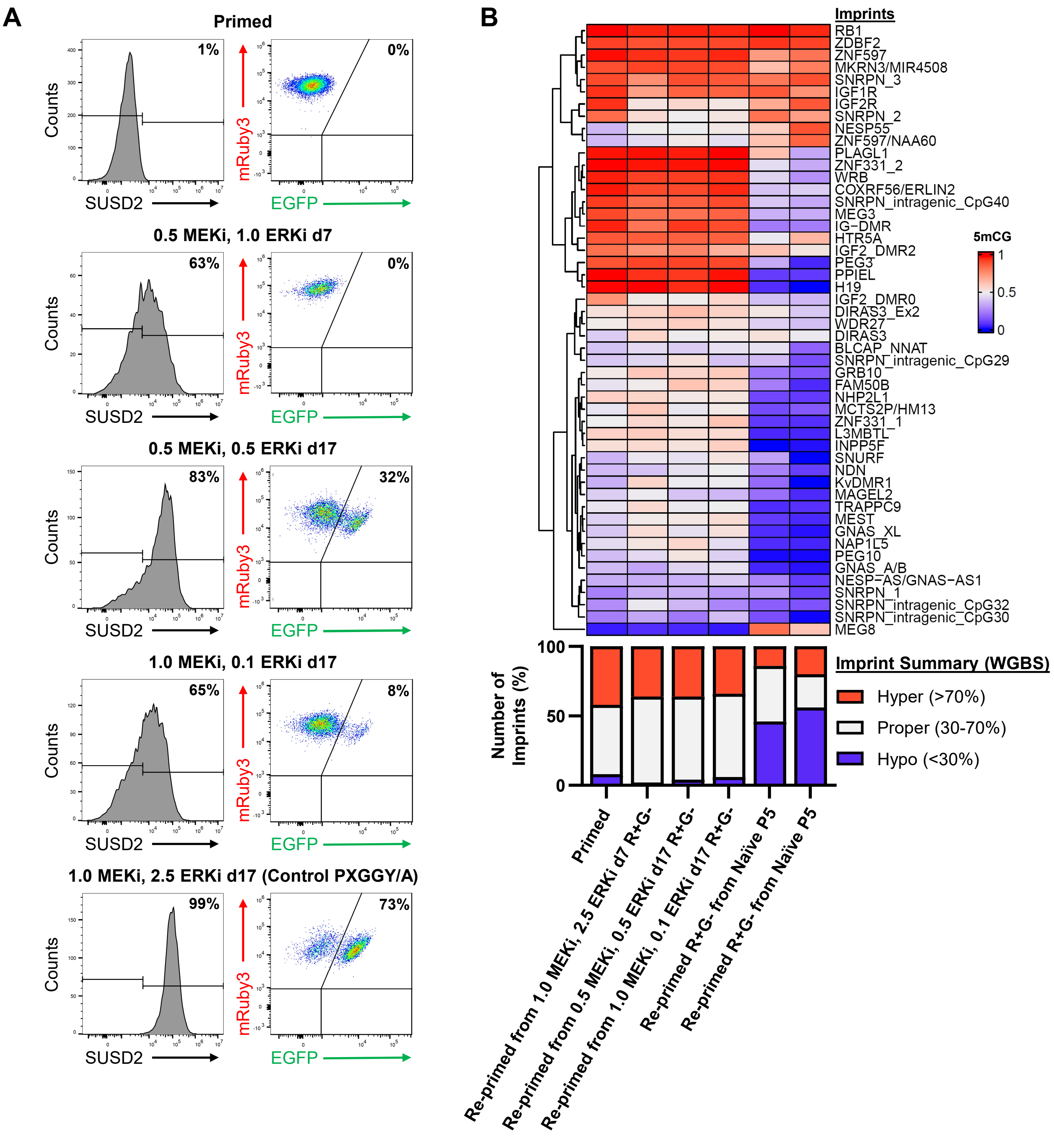
A
Primed
0.5 MEKi, 1.0 ERKi d7
0.5 MEKi, 0.5 ERKi d17
1.0 MEKi, 0.1 ERKi d17
1.0 MEKi, 2.5 ERKi d17 (Control PXGGY/A)
1%
0%
63%
0%
83%
32%
65%
8%
99%
73%
Counts
SUSD2
mRuby3
EGFP
B
Imprints
RB1
ZDBF2
ZNF597
MKRN3/MIR4508
SNRPN_3
IGF1R
IGF2R
SNRPN_2
NESP55
ZNF597/NAA60
PLAGL1
ZNF331_2
WRB
COXRF56/ERLIN2
SNRPN_intragenic_CpG40
MEG3
IG−DMR
HTR5A
IGF2_DMR2
PEG3
PPIEL
H19
IGF2_DMR0
DIRAS3_Ex2
WDR27
DIRAS3
BLCAP_NNAT
SNRPN_intragenic_CpG29
GRB10
FAM50B
NHP2L1
MCTS2P/HM13
ZNF331_1
L3MBTL
INPP5F
SNURF
NDN
KvDMR1
MAGEL2
TRAPPC9
MEST
GNAS_XL
NAP1L5
PEG10
GNAS_A/B
NESP−AS/GNAS−AS1
SNRPN_1
SNRPN_intragenic_CpG32
SNRPN_intragenic_CpG30
MEG8
5mCG
1
0.5
0
Number of Imprints (%)
100
50
0
Imprint Summary (WGBS)
Hyper (>70%)
Proper (30-70%)
Hypo (<30%)
Primed
Re-primed from 1.0 MEKi, 2.5 ERKi d7 R+G-
Re-primed from 0.5 MEKi, 0.5 ERKi d17 R+G-
Re-primed from 1.0 MEKi, 0.1 ERKi d17 R+G-
Re-primed R+G- from Naïve P5
Re-primed R+G- from Naïve P5

Figure S3

Figure S3: Methylation analysis of MEKi/ERKi titrated naïve cells followed by re-priming [Related to Figure 4]

- **A.** Flow cytometry plots of selected inhibitor combinations and timings shown in Figure 4A. mRuby3/EGFP plots (right) were gated for SUSD2+ to show reporter activity of naïve-converting cells (excluding primed sample).
- **B.** WGBS analysis and summary of imprints in H9-SRG re-primed samples following a brief titrated naïve pulse. Re-primed titrated samples (columns 2-4) were sorted for R+G-/SUSD2+ expression at the end of naïve treatment. When re-primed, these samples were >99.5% R^+G^-/SUSD2$^-$ and were therefore not sorted again. Re-primed titrated samples were compared to the primed (column 1) and re-primed R+G-/R+G+ samples (columns 5-6) from Figure 2.

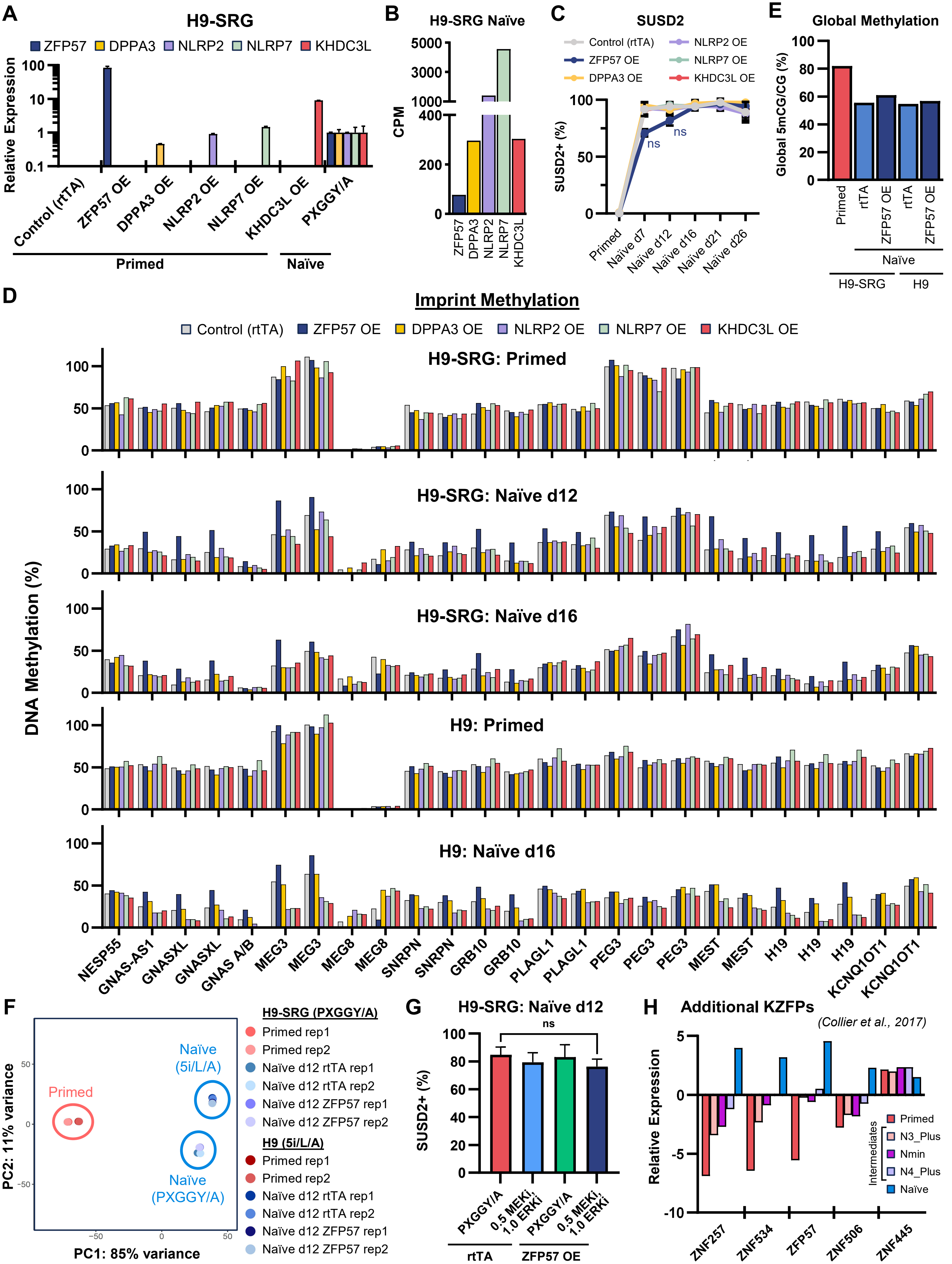
A
H9-SRG
ZFP57 DPPA3 NLRP2 NLRP7 KHDC3L
Relative Expression
Control (rtTA)
ZFP57 OE
DPPA3 OE
NLRP2 OE
NLRP7 OE
KHDC3L OE
PXGGY/A
Primed
Naïve
B
H9-SRG Naïve
CPM
C
SUSD2
Control (rtTA)
ZFP57 OE
DPPA3 OE
NLRP2 OE
NLRP7 OE
KHDC3L OE
SUSD2+ (%)
ns
Primed
Naïve d7
Naïve d12
Naïve d16
Naïve d21
Naïve d26
E
Global Methylation
Global 5mCG/CG (%)
Primed
rtTA
ZFP57 OE
Naïve
H9-SRG
H9
D
Imprint Methylation
H9-SRG: Primed
H9-SRG: Naïve d12
H9-SRG: Naïve d16
H9: Primed
H9: Naïve d16
DNA Methylation (%)
NESP55
GNAS-AS1
GNASXL
GNAS A/B
MEG3
MEG8
SNRPN
GRB10
PLAGL1
PEG3
MEST
H19
KCNQ1OT1
F
H9-SRG (PXGGY/A)
Primed rep1
Primed rep2
Naïve d12 rtTA rep1
Naïve d12 rtTA rep2
Naïve d12 ZFP57 rep1
Naïve d12 ZFP57 rep2
H9 (5i/L/A)
Naïve (5i/L/A)
Naïve (PXGGY/A)
PC1: 85% variance
PC2: 11% variance
G
H9-SRG: Naïve d12
SUSD2+ (%)
PXGGY/A
0.5 MEKi, 1.0 ERKi
rtTA
ZFP57 OE
H
Additional KZFPs
(Collier et al., 2017)
Relative Expression
Primed
N3_Plus
Nmin
N4_Plus
Naïve
Intermediates
ZNF257
ZNF534
ZFP57
ZNF506
ZNF445

Figure S4

Figure S4: Characterization of candidate imprint-protecting factor overexpression during primed-to-naïve resetting [Related to Figure 5]

- **A.** Gene expression analysis of candidate imprint-protecting factors in H9-SRG primed hPSCs. Samples were collected on the day of seeding for naïve resetting. Error bars represent standard deviation; n = 3.
- **B.** Expression of candidate imprint-protecting factors in H9-SRG naïve cells. Values were retrieved from RNA-seq data (Figure S2): Clone 1 Naïve P5 R+G+.
- **C.** Analysis of H9-SRG SUSD2 expression by flow cytometry during primed-to-naïve resetting of genetic overexpression samples. Error bars represent the standard error of the mean; n = 3 independent experiments. "ns" represents multiple unpaired t-test q-value > 0.1.
- **D.** MS-MLPA analysis of DNA methylation at imprints during primed-to-naïve resetting in each condition: H9-SRG cells at day 12 and 16 (PXGGY/A) and H9 cells at day 16 (5i/L/A).
- **E.** Global DNA methylation levels as measured by WGBS in control and ZFP57 overexpression samples during naïve resetting in PXGGY/A (H9-SRG, day 12) and 5i/L/A (H9, day 16).
- **F.** Principal component analysis of RNA-seq data from primed and naïve control and ZFP57 overexpression samples in PXGGY/A (H9-SRG, day 12) and 5i/L/A (H9, day 16).
- **G.** Analysis of the H9-SRG SUSD2+ population at day 12 of naïve resetting. Error bars represent the standard deviation; n = 3 independent experiments. "ns" represents unpaired t-test p-value > 0.1.
- **H.** Relative expression of KZFPs in primed, intermediate, and naïve cell populations (Collier *et al.*, 2017).

Table S1: Oligos used in this study

Name	Sequence (5' – 3')	Purpose
sgRNA-SNRPN	TCAACAGTATGCTAAGGTCTNGG	CRISPR/Cas9 targeting
SNRPN Left HA Forward (SNRPN_F)	AGGGCTTTTTGGATGTCAGTGT	Junction PCR/Sanger
SNRPN Right HA Reverse (SNRPN_R)	CATTCCCTCGCTGCCTACTA	Junction PCR
mRuby3_F	CACCAATTCAAATGCACAGG	Junction PCR/Sanger
mRuby3_R	ATTTGGCAACTGCCACTTCT	Junction PCR
EGFP_F	GGCAAGCTGACCCTGAAGTT	Junction PCR/Sanger
EGFP_R	TTCTGCTTGTCGGCCATGAT	Junction PCR
RPLP0_F	GCTTCCTGGAGGGTGTCC	qPCR
RPLP0_R	GGACTCGTTTGTACCCGTTG	qPCR
ZIC2_F	CCCTTCAAGGCCAAATACAA	qPCR
ZIC2_R	TGCATGTGCTTCTTCCTGTC	qPCR
SFRP2_F	ACGGCATCGAATACCAGAACA	qPCR
SFRP2_R	CTCGTCTAGGTCATCGAGGCA	qPCR
KLF17_F	CTGCCTGAGCGTGGTATGAG	qPCR
KLF17_R	TCATCCGGGAAGGAGTGAGA	qPCR
DNMT3L_F	TTCTGGATGTTCGTGGACAA	qPCR
DNMT3L_R	ACATCTGGGATGGTGACTGG	qPCR
ZFP57_F	ATGAAGAGAGATTGCTGGAGGG	qPCR
ZFP57_R	CCTCTGCATGCAAGGAAGAC	qPCR
DPPA3_F	GTTACTGGGCGGAGTTCGTA	qPCR
DPPA3_R	TGAAGTGGCTTGGTGTCTTG	qPCR
NLRP2_F	TCTGGACCTGGGTCAGAATC	qPCR
NLRP2_R	CCAGCAGCTTATTGAGTTCAT	qPCR
NLRP7_F	GGCCAGAATCATTTGTGGAA	qPCR
NLRP7_R	TTTTCTTTCACTTCCTCCAACA	qPCR
KHDC3L_F	CTAACGGCGAGGCTGAGAT	qPCR
KHDC3L_R	GCCTTTCCTGAGCCTTTCG	qPCR
ZNF445_F	CTACCGTCTGCACCGAGAA	qPCR
ZNF445_R	GCTTCTCCTGGGTGTGAATC	qPCR

Supplemental methods

Primed hPSC culture

H9 (WA09) hPSCs were obtained from the Genome Engineering & Stem Cell Center at the McDonnell Genome Institute of Washington University. Cell line identity was authenticated using STR profiling. GTW banding was performed after genome editing and showed a chromosomally normal karyotype. Primed hPSCs were cryopreserved in 10% DMSO solution and stored under liquid nitrogen in the vapor phase. Upon thawing, cells were warmed quickly, washed with appropriate medium, and plated for culture. Primed hPSCs were grown in mTeSR Plus medium on Matrigel-coated plates at 37°C, 20% O_2, and 5% CO_2. For general maintenance, cells were dissociated into small clumps using Dispase or ReLeSR and split at a ratio of 1:10-1:50. The medium was fully replaced every 1-2 days.

iMEF culture

Mouse embryonic fibroblasts (MEFs) were grown in fibroblast medium (DMEM/F12, 10% FBS, 1X GlutaMAX, and 1X Penicillin/Streptomycin) at 37°C, 20% O_2, and 5% CO_2. MEFs were growth-inactivated with 5 mg/mL mitomycin C for 3 hours. Inactivated MEFs (iMEFs) were single-cell dissociated with 0.25% Trypsin-EDTA and cryopreserved in 10% DMSO solution. For use as a feeder layer, iMEFs were thawed and plated at approximately 40,000 cells/cm^2.

Naïve media preparation

N2B27 basal medium was made using a 1:1 ratio of DMEM/F12:Neurobasal supplemented with 1X N2 supplement, 1X B27 supplement, 1X MEM non-essential amino acids solution, 1X GlutaMAX, 1% penicillin/streptomycin, 50 µg/mL BSA Fraction V, and 0.1 mM β-mercaptoethanol. PXGGY/A medium was made using N2B27 supplemented with 1 µM PD0325901, 2 µM XAV939, 2 µM Gö6983, 2.5 µM GDC-0994, 10 µM Y-27632, and 10 ng/mL Activin A (Khan et al., 2021). 5i/L/A medium was made using N2B27 supplemented with 1 µM PD0325901, 1 µM IM-12, 0.5

μM SB590885, 1 μM WH4-023, 10 μM Y-27632, 20 ng/mL recombinant human LIF, and 10 ng/mL Activin A (Fischer et al., 2022).

Primed-to-naïve hPSC resetting and naïve maintenance

Primed hPSCs were single-cell dissociated using TrypLE Express. hPSCs were seeded at a density of ~24,000 cells/cm^2 on iMEFs that were plated at least one day prior. Seeded cells were grown in mTeSR Plus supplemented with 10 μM Y-27632 for 2 days at 37°C, 5% O_2, and 5% CO_2. After 2 days, the medium was replaced with naïve medium, which designated day 0 of naïve resetting. Cultures were maintained at 37°C, 5% O_2, and 5% CO_2, and media were replaced every 1-2 days. Naïve hPSCs were split using TrypLE Express at a ratio of 1:1-1:4.

Re-priming naïve hPSCs

Naïve hPSCs were dissociated with TrypLE Express and seeded at ≥ 40,000 cells per well of a 6-well plate pre-coated with Matrigel. Cells were cultured in mTeSR Plus (supplemented with 10 μM Y-27632 for the first 24 hours) at 37°C, 20% O_2, and 5% CO_2. For the first passage, TrypLE Express was used to single-cell dissociate cells and mTeSR Plus was supplemented with 10 μM Y-27632 for 24 hours. For subsequent passages, ReLeSR was used to dissociate cells into small clumps and Y-27632 was omitted. Re-primed cells were used for analyses at >20 days of treatment.

Gene editing to establish the SNRPN dual-colored reporter

A single-guide RNA (sgRNA) targeting the *SNRPN* C-terminus and donor plasmids containing *P2A-mRuby3* and *P2A-EGFP* sequences were designed and validated by the Genome Engineering & Stem Cell Center at Washington University. Donor homology arms (left 796 bp; right 803 bp) were targeted to the regions flanking the *SNRPN* stop codon. H9 primed cells in a strong growth phase were single-cell dissociated with TrypLE Express. Cells were nucleofected

with 300 pmol sgRNA, 192 pmol Cas9 protein, and 1.5 µg of each donor plasmid using a Lonza 4-D nucleofector with P3 solution (Lonza, V4XP-3024). mTeSR Plus was supplemented with 10 µM Y-27632 for 24 hours post-nucleofection. Nine days after nucleofection, cells were single-cell sorted by FACS for mRuby3 or EGFP expression. No double-positive cells were detected. 1X RevitaCell (Gibco, A2644501) and 1X CloneR (STEMCELL Technologies, 05888) were used with mTeSR Plus to improve cell viability. Clones were expanded and integration was assessed by junction PCR. A clone containing one *P2A-mRuby3* allele and one wild-type allele (mRuby3/WT) was validated. This clone was nucleofected a second time using the sgRNA, Cas9 protein, and the *P2A-EGFP* donor plasmid. Cells were sorted at 5 cells/well to increase the likelihood of isolating the desired clone. Since properly-integrated EGFP would not be expressed in the primed hPSC state (as the maternal *SNRPN* allele is silenced), integration of EGFP was assessed by junction PCR. Two pools of cells showed the presence of *SNRPN-EGFP*. Both pools were single-cell sorted and screened again by junction PCR. Each pool provided a clone containing *SNRPN-mRuby3/SNRPN-EGFP*, which were deemed Clone 1 and Clone 2. Final sequences were validated by Sanger sequencing.

Live-cell imaging

Cells were imaged in culture media or FluoroBrite DMEM (Gibco, A1896701) using a Leica DMi8 inverted microscope. Scale bars were added using FIJI software (Schindelin et al., 2012).

Flow cytometry/FACS

Cells were single-cell dissociated using TrypLE Express and resuspended in their respective cell culture media. If desired, anti-human SUSD2-APC antibody (BioLegend, 327408) was used at a dilution of 1:100 for 45 minutes at 4°C in the dark. Cells were washed in Flow Buffer (1X HBSS + 5% FBS) to remove antibodies. Flow cytometry was performed in Flow Buffer using a Sony SY3200 Synergy cytometer (FACS-purification) or a Beckman Coulter CytoFLEX S cytometer

(analysis). Cells maintained in culture after FACS-purification were washed in 1X DPBS and culture media were supplemented with 10 µM Y-27632 (if not already in media) and 100 µg/mL Primocin for 24 hours. Data were processed with FlowJo v10.8.1 software.

Cell cycle analysis

Cells were single-cell dissociated using TrypLE Express, washed in 1X DPBS, and fixed in cold 70% ethanol for 30 min. Fixed cells were washed twice in 1X DPBS, then resuspended in 1 µg/mL DAPI (Thermo Scientific, 62248) in DPBS. Cells were incubated for 1 hour at room temperature and protected from light. Cells were not washed after DAPI staining. Flow cytometry was performed on a Beckman Coulter CytoFLEX S cytometer. Cell cycle analysis was performed using the Cell Cycle analysis tool in FlowJo v10.8.1 using a Watson (Pragmatic) fitting model.

Genomic DNA isolation

Genomic DNA (gDNA) was isolated from cells using the DNeasy Blood & Tissue Kit (Qiagen, 69504) per manufacturer's instructions.

MS-MLPA assay

MS-MLPA assays were performed on gDNA using reagents from MRC Holland Multilocus Imprinting Kits ME034-B1 or ME034-C1 according to manufacturer's instructions (Nygren et al., 2005). Briefly, 40 ng of gDNA were denatured and hybridized with the provided probemix for 16 hours. Samples were topped with 30 µL PCR-grade mineral oil to prevent evaporation. Each sample was then split in half. The first half underwent ligation, while the second half underwent ligation + digestion with HhaI enzyme. Products were amplified by PCR, then analyzed by capillary electrophoresis on an Applied Biosystems 3730xl Fragment Analyzer using the 6-FAM label. Peaks were called with GeneMapper v6.0 and inspected manually. The area under each peak was used to calculate DNA methylation levels.

Bulk RNA-sequencing and analysis

Total RNA integrity was determined using an Agilent Bioanalyzer or 4200 Tapestation. Library preparation was performed with 10 ng of total RNA with a Bioanalyzer RIN score greater than 8.0. ds-cDNA was prepared using the SMARTer Ultra Low RNA kit for Illumina Sequencing (Takara) per manufacturer's protocol. cDNA was fragmented using a Covaris E220 sonicator using peak incident power 18, duty factor 20%, cycles per burst 50 for 120 seconds. cDNA was blunt ended, had an A base added to the 3' ends, and then had Illumina sequencing adapters ligated to the ends. Ligated fragments were then amplified for 12-15 cycles using primers incorporating unique dual index tags. Fragments were sequenced on an Illumina NovaSeq-6000 (Figure 2) or an Illumina NovaSeq X Plus (Figures 4-5) using paired end reads extending 150 bases. Basecalls and demultiplexing were performed with Illumina's bcl2fastq software and a custom python demultiplexing program with a maximum of one mismatch in the indexing read. RNA-seq reads were then aligned to the Ensembl release 76 (Figure 2) or 101 (Figures 4-5) primary assembly with STAR version 2.5.1a or 2.7.9a (Dobin et al., 2013). Gene counts were derived from the number of uniquely aligned unambiguous reads by Subread:featureCount version 1.4.6-p5 or 2.0.3 (Liao et al., 2014). Isoform expression of known Ensembl transcripts were estimated with Salmon version 0.8.2 or 1.5.2 (Patro et al., 2017). Sequencing performance was assessed for the total number of aligned reads, total number of uniquely aligned reads, and features detected. The ribosomal fraction, known junction saturation, and read distribution over known gene models were quantified with RSeQC version 2.6.2 or 4.0 (Wang et al., 2012).

All gene counts were then imported into the R/Bioconductor package EdgeR (Robinson et al., 2010) and TMM normalization size factors were calculated to adjust for samples for differences in library size. Ribosomal genes and genes not expressed in the smallest group size minus one samples greater than one count-per-million were excluded from further analysis. The TMM size

factors and the matrix of counts were then imported into the R/Bioconductor package Limma (Ritchie et al., 2015). Weighted likelihoods based on the observed mean-variance relationship of every gene and sample were then calculated for all samples and the count matrix was transformed to moderated log 2 counts-per-million with Limma's voomWithQualityWeights (Liu et al., 2015). The performance of all genes was assessed with plots of the residual standard deviation of every gene to their average log-count with a robustly fitted trend line of the residuals.

Differential expression analysis was performed to analyze for differences between conditions. The R/Bioconductor package EnhancedVolcano version 1.12.0 (Blighe et al., 2021) was used to plot log 2 fold changes versus negative log 10 P-values for each term. Principal component analysis was performed using DESeq2 version 1.34.0 (Love et al., 2014). Using results filtered for only those genes with Benjamini-Hochberg false-discovery rate adjusted p-values less than or equal to 0.05, Gene Ontology (GO) terms, MSigDb, and KEGG pathways were detected using the R/Bioconductor package GAGE (Luo et al., 2009) to test for changes in expression of the reported log 2 fold changes reported by Limma in each term versus the background log 2 fold changes of all genes found outside the respective term. Bar plots to visualize GO terms were made using ggplot2 version 3.4.4 (Wickham, 2016). Heatmaps of log 2 fold changes were plotted using the R/Bioconductor package ComplexHeatmap version 2.10.0 (Gu et al., 2016). Gene set enrichment analysis was performed by ranking all genes using the R/Bioconductor package fgsea (Korotkevich et al., 2019). Enrichment for MSigDb Hallmark gene sets was performed with GSEA version 4.3.3 (Subramanian et al., 2005) using the pre-ranked gene list and 50,000 permutations.

WGBS analysis

Paired-end Fastq files were trimmed with TrimGalore version 0.6.6 (Krueger et al., 2020) and reads were aligned with Bismark version 0.23.0 (Krueger and Andrews, 2011). The Methpipe package version 4.1.1 was then used with the indicated tools to remove duplicate reads

(duplicate-remover), calculate methylation levels (methcounts), extract and merge symmetric CpGs (symmetric-cpgs), and calculate methylation statistics for regions of interest (roimethstat) (Song et al., 2013). Heatmaps of imprinted regions (Court et al., 2014) were plotted using the R/Bioconductor package ComplexHeatmap version 2.10.0 (Gu *et al.*, 2016).

Browser tracks

FastqQC version 0.11.9 was used to assess the quality of the raw reads. Subsequently, the paired-end reads were trimmed to remove adaptor sequences and low-quality reads with Cutadapt version 4.0 and reassessed using FastqQC. The human reference genome hg38 was first bisulfite converted using Bismark version 0.20.0 (Krueger and Andrews, 2011). The paired-end reads were aligned to the hg38 bisulfite-converted genome and deduplicated using 'deduplicate_bismark'. DNA methylation levels were calculated using 'bismark_methylation_extractor' and displayed in a methylC format on the WashU Epigenome Browser (Zhou et al., 2011).

KZFP analysis

The cutoff for additional KZFPs of interest was set at "Naïve" - "N3_Plus" > 3 (Collier et al., 2017).

Supplemental references

Blighe, K., Rana, S., and Lewis, M. (2021). EnhancedVolcano: Publication-ready volcano plots with enhanced colouring and labeling. 10.18129/B9.bioc.EnhancedVolcano.

Collier, A.J., Panula, S.P., Schell, J.P., Chovanec, P., Plaza Reyes, A., Petropoulos, S., Corcoran, A.E., Walker, R., Douagi, I., Lanner, F., and Rugg-Gunn, P.J. (2017). Comprehensive Cell Surface Protein Profiling Identifies Specific Markers of Human Naive and Primed Pluripotent States. Cell stem cell *20*, 874-890 e877. 10.1016/j.stem.2017.02.014.

Court, F., Tayama, C., Romanelli, V., Martin-Trujillo, A., Iglesias-Platas, I., Okamura, K., Sugahara, N., Simón, C., Moore, H., Harness, J.V., et al. (2014). Genome-wide parent-of-origin DNA methylation analysis reveals the intricacies of human imprinting and suggests a germline methylation-independent mechanism of establishment. Genome Research *24*, 554-569. 10.1101/gr.164913.113.

Dobin, A., Davis, C.A., Schlesinger, F., Drenkow, J., Zaleski, C., Jha, S., Batut, P., Chaisson, M., and Gingeras, T.R. (2013). STAR: ultrafast universal RNA-seq aligner. Bioinformatics *29*, 15-21. 10.1093/bioinformatics/bts635.

Fischer, L.A., Khan, S.A., and Theunissen, T.W. (2022). Induction of Human Naïve Pluripotency Using 5i/L/A Medium. In Human Naïve Pluripotent Stem Cells, P. Rugg-Gunn, ed. (Springer US), pp. 13-28. 10.1007/978-1-0716-1908-7_2.

Gu, Z., Eils, R., and Schlesner, M. (2016). Complex heatmaps reveal patterns and correlations in multidimensional genomic data. Bioinformatics *32*, 2847-2849. 10.1093/bioinformatics/btw313.

Khan, S.A., Park, K.-m., Fischer, L.A., Dong, C., Lungjangwa, T., Jimenez, M., Casalena, D., Chew, B., Dietmann, S., Auld, D.S., et al. (2021). Probing the signaling requirements for naive human pluripotency by high-throughput chemical screening. Cell Reports *35*, 109233. 10.1016/j.celrep.2021.109233.

Korotkevich, G., Sukhov, V., and Sergushichev, A. (2019). Fast gene set enrichment analysis. bioRxiv. 10.1101/060012.

Krueger, F., and Andrews, S.R. (2011). Bismark: a flexible aligner and methylation caller for Bisulfite-Seq applications. Bioinformatics *27*, 1571-1572. 10.1093/bioinformatics/btr167.

Krueger, F., James, F., Ewels, P., Afyounian, E., Weinstein, M., Schuster-Boeckler, B., and Hulselmans, G. (2020). TrimGalore v0.6.6.

Liao, Y., Smyth, G.K., and Shi, W. (2014). featureCounts: an efficient general purpose program for assigning sequence reads to genomic features. Bioinformatics *30*, 923-930. 10.1093/bioinformatics/btt656.

Liu, R., Holik, A.Z., Su, S., Jansz, N., Chen, K., Leong, H.S., Blewitt, M.E., Asselin-Labat, M.-L., Smyth, G.K., and Ritchie, M.E. (2015). Why weight? Modelling sample and observational level variability improves power in RNA-seq analyses. Nucleic Acids Research *43*, e97-e97. 10.1093/nar/gkv412.

Love, M.I., Huber, W., and Anders, S. (2014). Moderated estimation of fold change and dispersion for RNA-seq data with DESeq2. Genome Biology *15*, 550. 10.1186/s13059-014-0550-8.

Luo, W., Friedman, M.S., Shedden, K., Hankenson, K.D., and Woolf, P.J. (2009). GAGE: generally applicable gene set enrichment for pathway analysis. BMC Bioinformatics *10*, 161. 10.1186/1471-2105-10-161.

Nygren, A.O.H., Ameziane, N., Duarte, H.M.B., Vijzelaar, R.N.C.P., Waisfisz, Q., Hess, C.J., Schouten, J.P., and Errami, A. (2005). Methylation-Specific MLPA (MS-MLPA): simultaneous detection of CpG methylation and copy number changes of up to 40 sequences. Nucleic Acids Research *33*, e128-e128. 10.1093/nar/gni127.

Patro, R., Duggal, G., Love, M.I., Irizarry, R.A., and Kingsford, C. (2017). Salmon provides fast and bias-aware quantification of transcript expression. Nature Methods *14*, 417-419. 10.1038/nmeth.4197.

Ritchie, M.E., Phipson, B., Wu, D., Hu, Y., Law, C.W., Shi, W., and Smyth, G.K. (2015). limma powers differential expression analyses for RNA-sequencing and microarray studies. Nucleic Acids Research *43*, e47-e47. 10.1093/nar/gkv007.

Robinson, M.D., McCarthy, D.J., and Smyth, G.K. (2010). edgeR: a Bioconductor package for differential expression analysis of digital gene expression data. Bioinformatics *26*, 139-140. 10.1093/bioinformatics/btp616.

Schindelin, J., Arganda-Carreras, I., Frise, E., Kaynig, V., Longair, M., Pietzsch, T., Preibisch, S., Rueden, C., Saalfeld, S., Schmid, B., et al. (2012). Fiji: an open-source platform for biological-image analysis. Nature Methods *9*, 676-682. 10.1038/nmeth.2019.

Song, Q., Decato, B., Hong, E.E., Zhou, M., Fang, F., Qu, J., Garvin, T., Kessler, M., Zhou, J., and Smith, A.D. (2013). A Reference Methylome Database and Analysis Pipeline to Facilitate Integrative and Comparative Epigenomics. PLOS ONE *8*, e81148. 10.1371/journal.pone.0081148.

Subramanian, A., Tamayo, P., Mootha, V.K., Mukherjee, S., Ebert, B.L., Gillette, M.A., Paulovich, A., Pomeroy, S.L., Golub, T.R., Lander, E.S., and Mesirov, J.P. (2005). Gene set enrichment analysis: A knowledge-based approach for interpreting genome-wide expression profiles. Proceedings of the National Academy of Sciences *102*, 15545-15550. 10.1073/pnas.0506580102.

Wang, L., Wang, S., and Li, W. (2012). RSeQC: quality control of RNA-seq experiments. Bioinformatics *28*, 2184-2185. 10.1093/bioinformatics/bts356.

Wickham, H. (2016). ggplot2: Elegant Graphics for Data Analysis (Springer-Verlag New York).

Zhou, X., Maricque, B., Xie, M., Li, D., Sundaram, V., Martin, E.A., Koebbe, B.C., Nielsen, C., Hirst, M., Farnham, P., et al. (2011). The Human Epigenome Browser at Washington University. Nature Methods *8*, 989-990. 10.1038/nmeth.1772.
