# Supplementary material for: Tracking and mitigating imprint erasure during induction of naive human pluripotency at single-cell resolution
Source: Stem Cell Reports. 2025 Feb 13;20(3):102419. doi: 10.1016/j.stemcr.2025.102419 (PMC11960550; doi:10.1016/j.stemcr.2025.102419)
Supplement: Document S2. Article plus supplemental information [file mmc2.pdf]

Stem Cell Reports
Article
OPEN ACCESS

Tracking and mitigating imprint erasure during induction of naive human pluripotency at single-cell resolution

Laura A. Fischer,[1] Brittany Meyer,[1] Monica Reyes,[2] Joseph E. Zemke,[1] Jessica K. Harrison,[3] Kyoung-mi Park,[1] Ting Wang,[3] Harald Jüppner,[2] Sabine Dietmann,[1,4] and Thorold W. Theunissen[1,5,*]

[1]Department of Developmental Biology and Center of Regenerative Medicine, Washington University School of Medicine, St. Louis, MO, USA
[2]Endocrine Unit, Department of Medicine and Pediatric Nephrology Unit, Department of Pediatrics, Massachusetts General Hospital and Harvard Medical School, Boston, MA, USA
[3]Department of Genetics, The Edison Family Center for Genome Sciences & Systems Biology, Washington University School of Medicine, St. Louis, MO, USA
[4]Institute for Informatics (I[2]), Washington University School of Medicine, St. Louis, MO, USA
[5]Lead contact
*Correspondence: t.theunissen@wustl.edu
https://doi.org/10.1016/j.stemcr.2025.102419

SUMMARY

Naive human pluripotent stem cells (hPSCs) model the pre-implantation epiblast. However, parent-specific epigenetic marks (imprints) are eroded in naive hPSCs, which represents an important deviation from the epiblast *in vivo*. To track the dynamics of imprint erasure during naive resetting in real time, we established a dual-colored fluorescent reporter at both alleles of the imprinted *SNRPN* locus. During primed-to-naive resetting, SNRPN expression becomes biallelic in most naive cells, and biallelic SNRPN expression is irreversible upon re-priming. We utilized this live-cell reporter to evaluate chemical and genetic strategies to minimize imprint erasure. Decreasing the level of MEK/ERK inhibition or overexpressing the KRAB zinc-finger protein ZFP57 protected a subset of imprints during naive resetting. Combining these two strategies protected imprint levels to a further extent than either strategy alone. This study offers an experimental tool to track and enhance imprint stability during transitions between human pluripotent states *in vitro*.

INTRODUCTION

Parent-specific epigenetic marks (imprints) are crucial for normal growth and development, yet their mechanisms of establishment and maintenance are not fully understood. Landmark studies in mice demonstrated that imprinted genes are expressed from either the maternal or paternal allele and are essential for the development of embryonic and extraembryonic tissues (Barton et al., 1984; McGrath and Solter, 1984; Surani et al., 1984). In humans, approximately 200 imprinted genes have been discovered, and improper imprinting can manifest as growth restriction, obesity, intellectual disabilities, behavioral abnormalities, and an increased risk of certain cancers (Kalish et al., 2014; Monk et al., 2019; Peters, 2014; Tucci et al., 2019). In addition, aberrant expression of imprinted genes has been implicated in developmental defects in non-human primate embryos generated by somatic cell nuclear transfer (Liao et al., 2024). The evolution of genomic imprinting in placental mammals is thought to reflect the competition between maternal and paternal genomes over resources during gestation (Wilkins and Haig, 2003). Moreover, imprinting promotes the exchange of genetic information by raising barriers to uniparental reproduction (Li et al., 2018).

Advances in modeling early human development have surged from the delineation of pluripotent cell states, namely that of naive and primed pluripotency (Nichols and Smith, 2009). Naive human pluripotent stem cells (hPSCs) align closely with the *in vivo* pre-implantation epiblast in that they share a similar transcriptional profile (including that of transposable elements) (Takashima et al., 2014; Theunissen et al., 2014, 2016), demonstrate X chromosome dampening (Dror et al., 2024; Sahakyan et al., 2017), and possess the developmental plasticity to generate embryonic and extraembryonic tissues (Castel et al., 2020; Cinkornpumin et al., 2020; Dattani et al., 2024; Dong et al., 2020; Guo et al., 2021; Io et al., 2021; Okubo et al., 2024). However, a persistent issue hampering *bona fide* naive hPSCs is the erosion of imprints (Pastor et al., 2016; Theunissen et al., 2016). Current naive culture media require fibroblast growth factor (FGF) pathway inhibition to maintain naive identity. Interestingly, FGF pathway inhibition has also been suggested to cause greater loss of imprinting (LOI) (Keshet and Benvenisty, 2021). This complicates the accurate study of naive hPSCs. Furthermore, imprints and proper monoallelic gene expression do not return upon transition back to the primed state of pluripotency, a process known as "re-priming," or subsequent differentiation (Keshet and Benvenisty, 2021; Theunissen et al., 2016). Thus, aberrant imprinting in naive hPSCs hinders developmental studies of lineage specification and the potential applications of naive hPSCs in regenerative medicine.

Several studies have surveyed the LOI found in cultured pluripotent cells by analyzing the expression of imprinted genes bearing distinguishing parental single-nucleotide polymorphisms (Bar et al., 2017; Keshet and Benvenisty,

© 2025 The Author(s).
Published by Elsevier Inc. on behalf of International Society for Stem Cell Research.
This is an open access article under the CC BY license (http://creativecommons.org/licenses/by/4.0/).

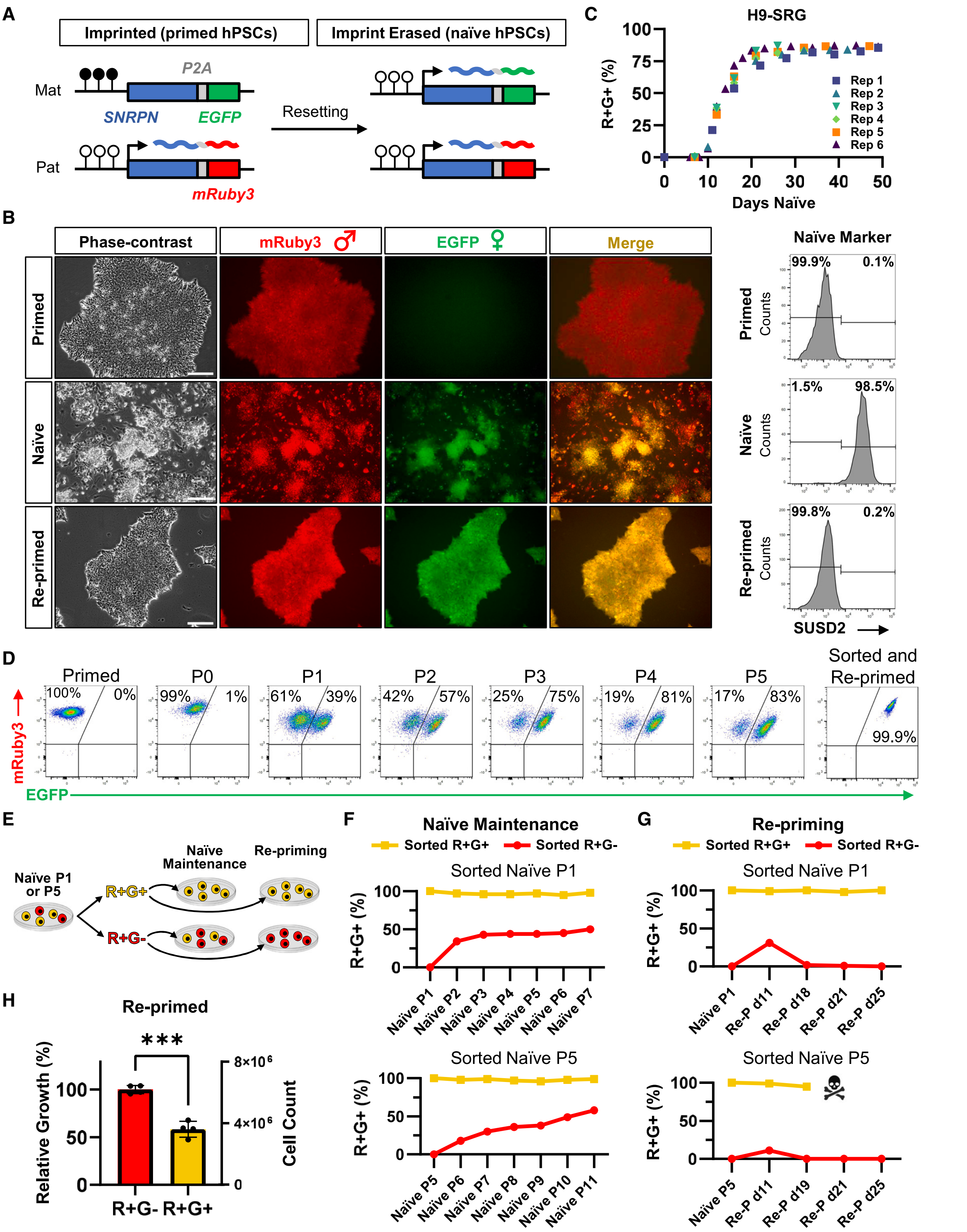

(legend on next page)

2021; Rugg-Gunn et al., 2007). However, most studies pertaining to LOI have lacked the ability to monitor imprint integrity in live cultures. Stelzer et al. created a live-cell reporter for DNA methylation in mouse embryonic stem cells (ESCs) by utilizing a minimal promoter that is sensitive to methylation changes of adjacent sequences (Stelzer et al., 2015). However, there remains an unmet need for a reporter of imprinted gene expression in hPSCs that enables real-time visualization of LOI at single-cell resolution.

Here, we created a dual-colored fluorescent reporter at the endogenous *SNRPN* locus in primed hPSCs. We show that SNRPN acquires biallelic expression during primed-to-naive resetting, which is irreversible upon re-priming. Our reporter accurately reflects methylation at the *SNRPN* locus and is a proxy for global methylation levels. Titrating FGF pathway inhibition during naive resetting enabled us to capture a naive, imprint-protected cell population. We also demonstrate the imprint-protective effects of a KRAB zinc-finger protein, ZFP57, when ectopically expressed during the generation of naive hPSCs. When combined, these two imprint protection strategies produced an even greater imprint-protective effect. These findings provide an important step toward improving the imprint fidelity of naive hPSCs and their applications for studies of human development and regeneration.

RESULTS

A live-cell reporter hPSC line displays allele-specific SNRPN expression

To track the stability of parent-specific imprints in real time, we set out to build a dual-colored fluorescent reporter at a representative imprinted locus. We selected the *SNRPN* locus because it shows stable monoallelic expression in primed hPSCs, and the associated imprint control center (ICR) undergoes demethylation during primed-to-naive resetting, resulting in biallelic expression of the downstream *SNRPN* transcript (Pastor et al., 2016; Rugg-Gunn et al., 2007; Theunissen et al., 2016). In addition, *SNRPN* is robustly expressed in both primed and naive hPSC conditions, an important prerequisite for an endogenous live-cell reporter. We inserted a *P2A-mRuby3* sequence on one allele of *SNRPN* and a *P2A-EGFP* sequence on the other allele in H9 human embryonic stem cells (hESCs) using CRISPR-Cas9-mediated genome editing (Figures 1A and S1A). Several clones were generated, and sequence integration was validated by junction PCR (Figure S1B). We designate this genotype as H9-SNRPN-mRuby3-EGFP (H9-SRG). In the primed state, mRuby3 was highly expressed and EGFP was not expressed, suggesting that mRuby3 was integrated into the active, paternal *SNRPN* allele, while EGFP was integrated into the inactive, maternal *SNRPN* allele (Figures 1B and S1C). Consistent with LOI and a switch from mono- to biallelic *SNRPN* expression, EGFP became active in the majority of naive cells within 2–3 passages in PXGGY/A naive induction medium (Khan et al., 2021), resulting in a population of mostly double-positive mRuby3+/EGFP+ (R+G+) cells (Figures 1B and S1C). We confirmed the acquisition of naive identity in these H9-SRG cells by flow cytometry for the naive-specific cell surface marker SUSD2. Additional markers for primed and naive pluripotency were confirmed by qPCR (Figure S1D). G-banding of primed H9-SRG clones confirmed normal karyotypes (Figure S1E).

We then carefully traced the kinetics of EGFP activation across primed-to-naive resetting, which displayed high reproducibility across independent replicates (Figures 1C and 1D). The earliest we could detect EGFP expression was on day 9 of primed-to-naive resetting, with 50% EGFP activation reached around day 14. Interestingly, a small fraction of the naive population remained mRuby3+/EGFP− (R+G−), even when cultured for >5 passages. We fluorescence-activated cell sorting (FACS)-purified the H9-SRG cells undergoing resetting at passage 1 (P1) and passage 5 (P5) into R+G− and R+G+ populations and maintained them

Figure 1. The H9-SRG dual-colored fluorescent reporter displays allele-specific *SNRPN* expression

(A) Schematic of the H9-SRG reporter at the *SNRPN* locus. Closed circles indicate DNA methylation. Open circles indicate lack of DNA methylation.

(B) Images and flow cytometry data for H9-SRG cells (clone 1) in the primed, naive, and re-primed states. Scale bar, 200 μm. Images are representative of four time points. See Figure S1C for clone 2.

(C) Analysis of H9-SRG reporter activity during primed-to-naive resetting across six independent experiments.

(D) Flow cytometry data of H9-SRG cells during primed-to-naive resetting. Plots are gated for SUSD2+ to show reporter activity of naive-converting cells (excluding primed and re-primed samples).

(E) Schematic of H9-SRG naive cells sorted by reporter activity and then maintained in naive conditions or re-primed.

(F) Time course of the H9-SRG reporter activity of cells sorted at naive P1 (top) or naive P5 (bottom) and maintained in naive conditions. Reporter expression was measured by flow cytometry.

(G) Time course of the H9-SRG reporter activity of cells sorted at naive P1 (top) or naive P5 (bottom) and immediately re-primed. Reporter expression was measured by flow cytometry. Naive P5 sorted R+G+ cells did not survive past 19 days of re-priming.

(H) Proliferation assay of re-primed R+G− and R+G+ populations that had been sorted at naive P1. Error bars represent standard deviation. ***unpaired t test p value = 0.0001; n = 4 independent experiments.

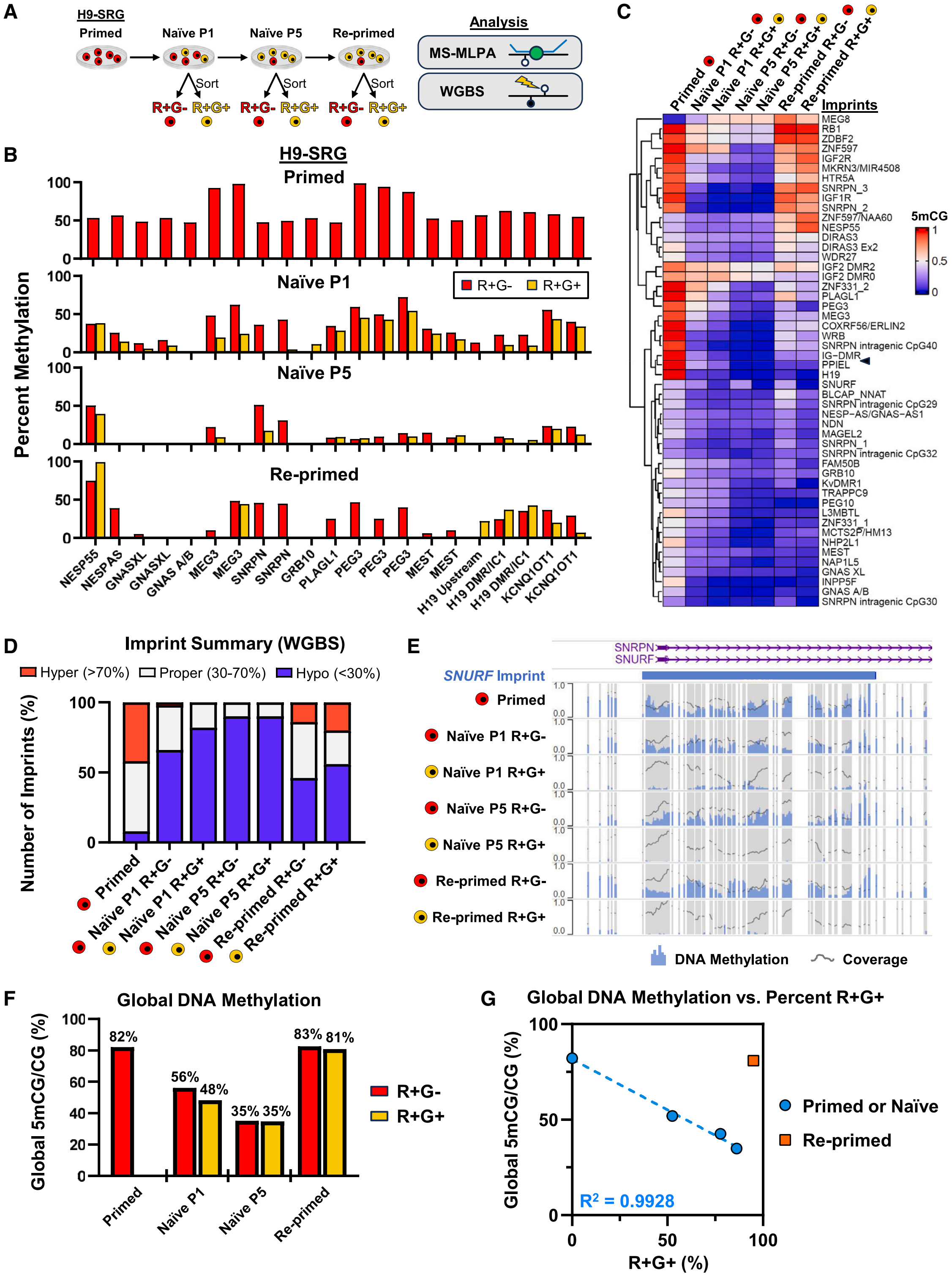

Figure 2. H9-SRG reporter activity corresponds to methylation at the *SNURF* locus and is a proxy for global methylation levels

(A) Schematic of H9-SRG primed-to-naive resetting followed by re-priming. Cells were sorted by reporter activity at naive P1, naive P5, and re-primed time points and analyzed by MS-MLPA and WGBS.

(B) MS-MLPA analysis of DNA methylation at imprints. Loci are limited to those captured in the assay.

(legend continued on next page)

separately in naive culture (Figures 1E and 1F). The R+G− sorted population gradually repopulated the EGFP+ population, while the R+G+ sorted population remained fully EGFP+. These observations suggest that naive cells are continuously subject to imprint erasure under self-renewing naive conditions and that biallelic *SNRPN* expression is irreversible once imprinting at the maternal allele has been erased.

We next asked whether biallelic *SNRPN* expression was reversible upon re-exposure to primed culture conditions, which causes a return to an early post-implantation-like pluripotent state (An et al., 2020; Theunissen et al., 2016). Cells were FACS-purified at P1 and P5 of primed-to-naive resetting into R+G−/R+G+ populations and immediately re-primed (Figure 1G). Over time, we observed that the sorted R+G+ cells maintained high EGFP percentages, while R+G− cells maintained their EGFP− status throughout re-priming. However, we also observed a pronounced growth disadvantage of the R+G+ cells during re-priming compared to their R+G− counterparts. In fact, the EGFP+ cells that were sorted at P5 of primed-to-naive resetting could not be maintained for more than 19 days in a primed medium. To explore this growth difference further, we performed a proliferation assay on the two re-primed populations sorted from naive P1. The biallelic R+G+ population grew at 58% of the rate of the monoallelic R+G− population (Figure 1H). We then asked whether this growth difference could be due to cell-cycle differences. Based on cell-cycle analysis by flow cytometry, we observed no significant difference in the proportions of cells in G0/G1 phase, S phase, or G2 phase between R+G− and R+G+ populations (Figure S1F). Overall, these data suggest that the transition to a stable post-implantation identity is impeded in naive cells displaying biallelic SNRPN expression and that imprinted SNRPN expression can be preserved by returning EGFP− naive cells to primed culture conditions.

SNRPN reporter activity correlates with methylation at the *SNURF* locus and is a proxy for global methylation levels

To assess the correlation between mRuby3/EGFP expression and DNA methylation at imprinted loci, we FACS-purified R+G−/R+G+ cells at naive P1 and naive P5 and after re-priming and then performed imprint analysis by methylation-specific multiplex ligation-dependent probe amplification (MS-MLPA) (Nygren et al., 2005) and whole-genome bisulfite sequencing (WGBS) (Figure 2A). MS-MLPA analysis indicated that methylation at imprinted loci was slightly higher in R+G− cells than R+G+ cells at P1, but this difference was diminished by P5 (Figure 2B). Re-primed R+G− cells also displayed increased methylation at a subset of imprints compared to their R+G+ counterparts. WGBS analysis provided a more thorough examination of methylation. A comparison of imprinting regions, as previously defined (Court et al., 2014), revealed several key findings (Figures 2C and 2D). First, while primed hESCs have several "properly" imprinted regions (30%–70% methylation), they also possess a substantial number of hypermethylated ICRs (>70% methylation). At naive P1 and P5, imprints were largely hypomethylated (<30% methylation) in both R+G− and R+G+ samples. However, methylation was slightly elevated at imprinted loci in R+G− compared to R+G+ cells at P1 of primed-to-naive resetting. Consistent with the MS-MLPA data, this difference largely disappeared by P5. Upon re-priming, a subset of hypomethylated imprints regained significant methylation, but most remained hypomethylated, in accordance with prior observations that imprint erasure is irreversible (Pastor et al., 2016; Theunissen et al., 2016). While re-primed R+G− cells displayed elevated methylation at several imprints compared to re-primed R+G+ cells, the only ICR that consistently showed higher methylation in R+G− compared to R+G+ samples at all examined time points was *SNURF*, which is the ICR located most closely to the *SNRPN* transcriptional start site (Figure 2E). We conclude that SNRPN reporter activity correlates most strongly with methylation of the nearest differentially methylated region (DMR). Like the majority of imprinted loci, the *SNURF* DMR undergoes DNA demethylation within 5 passages of primed-to-naive resetting. However, a subset of naive cells (ca. 15%) retain monoallelic methylation of *SNURF*, but not other imprinted loci, which suggests that this ICR may be subject to unique regulatory mechanisms.

Additionally, we examined to what extent SNRPN reporter activity correlates with global 5-methylcytosine followed by guanine (5mCG) levels. Global 5mCG levels were about 80% in the primed and re-primed states and about 48%–56% and 35% at P1 and P5 of primed-to-naive resetting, respectively (Figure 2F). The elevated 5mCG level in the primed state and reduced 5mCG level in the naive state are consistent with DNA methylation patterns in human

(C) WGBS analysis of DNA methylation at imprinted DMRs.
(D) Summary of hyper-, hypo-, and properly methylated imprints based on WGBS analysis.
(E) Browser tracks of DNA methylation at the *SNURF* locus. Vertical gray bars represent locations of CpG dinucleotides.
(F) Global DNA methylation levels as measured by WGBS.
(G) Analysis of global DNA methylation compared to H9-SRG reporter activity. The R^2 value was calculated using a linear regression model on primed and naive data points.

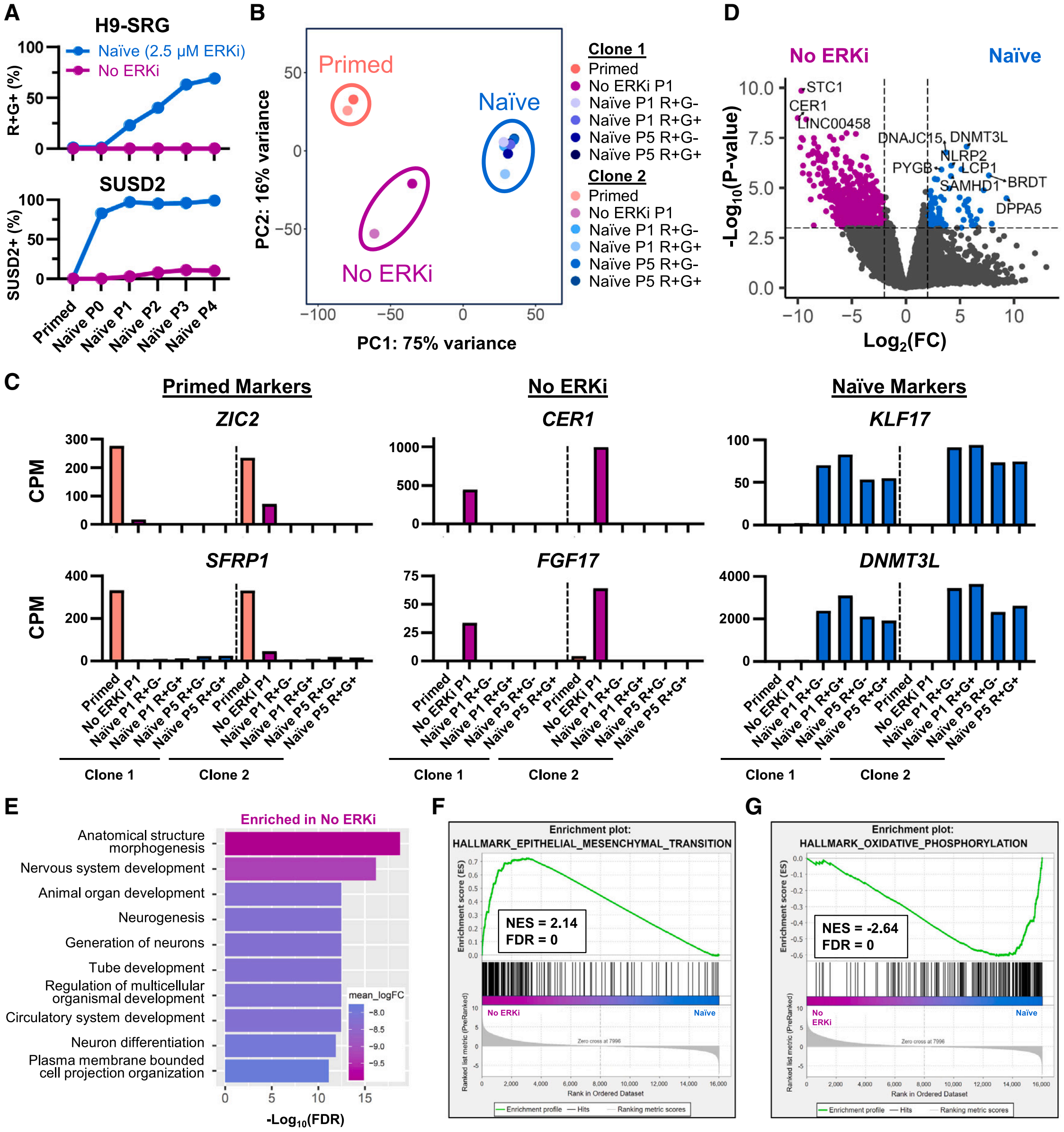

Figure 3. Absence of ERKi during primed-to-naive resetting precludes acquisition of *bona fide* naive identity

(A) Analysis of H9-SRG reporter activity and SUSD2 expression by flow cytometry during primed-to-naive resetting using PXGGY/A or upon omission of the ERK inhibitor GDC-0994 (No ERKi).

(B) Principal-component analysis of RNA-seq data from primed, no ERKi, and naive (PXGGY/A) samples using two independent clones.

(C) Marker gene expression of primed, no ERKi, and naive (PXGGY/A) samples by RNA-seq using two independent clones.

(D) Volcano plot of DEGs between no ERKi and naive (PXGGY/A) samples at P1 of naive resetting; $n = 2$ independent clones.

(E) Gene ontology analysis of the top ten GO Biological Process terms differentially expressed between no ERKi and naive (PXGGY/A) P1 samples using two independent clones.

(F) Gene set enrichment analysis of the top Hallmark signature (epithelial-mesenchymal transition) enriched in no ERKi compared to naive (PXGGY/A) using two independent clones. NES, normalized enrichment score.

(G) Gene set enrichment analysis of the top Hallmark signature (oxidative phosphorylation) enriched in naive (PXGGY/A) compared to no ERKi using two independent clones. NES, normalized enrichment score.

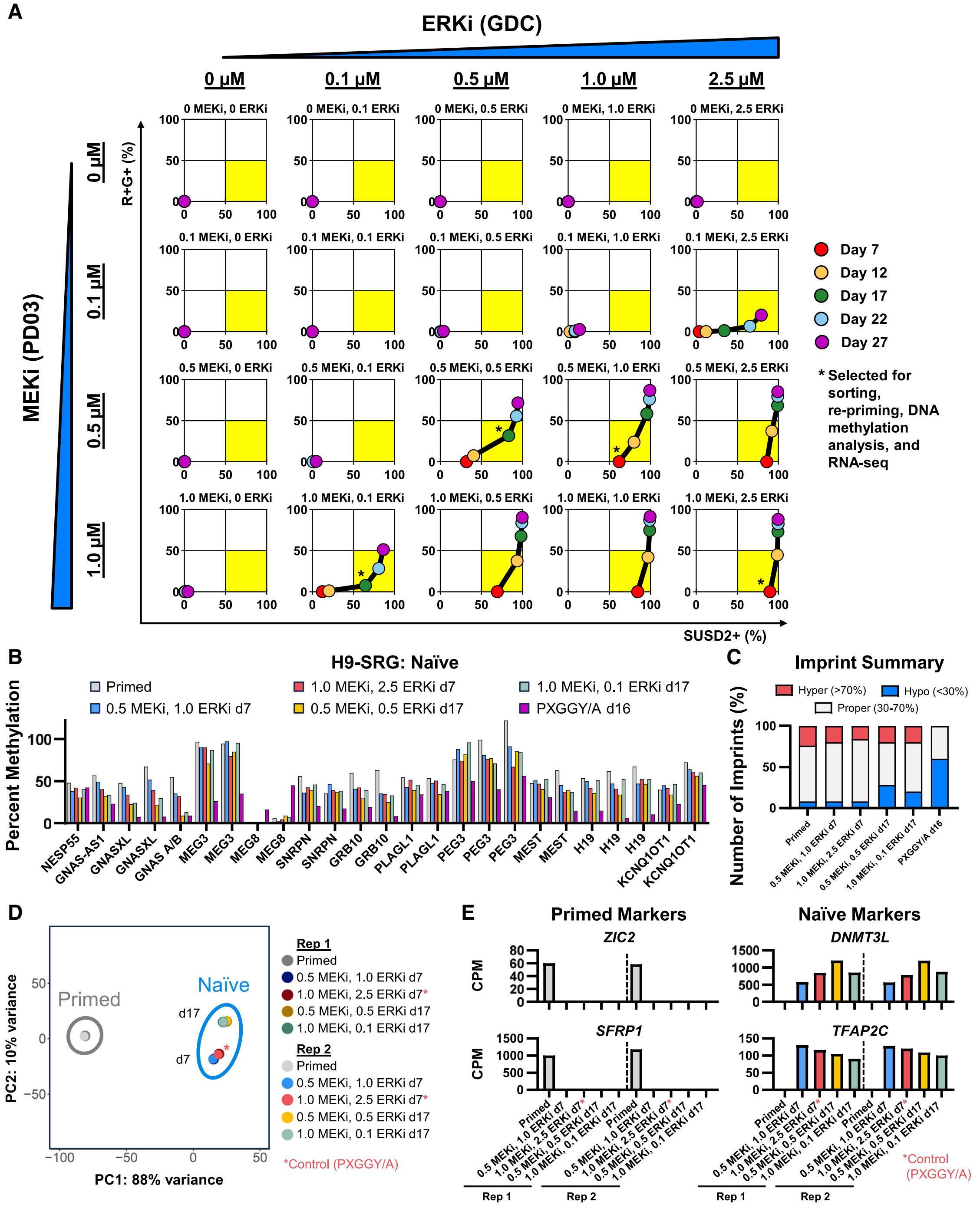

Figure 4. Modulating FGF signaling to enhance imprint fidelity in naive hPSCs

(A) Analysis of H9-SRG reporter activity and SUSD2 expression by flow cytometry during primed-to-naive resetting using titrated amounts of MEKi (PD03) and ERKi (GDC). The lower right quadrant of each plot (yellow highlight) represents cells expressing the naive marker SUSD2 and predominantly maintaining monoallelic expression of SNRPN.

(B) MS-MLPA analysis of DNA methylation at imprinted DMRs in MEKi/ERKi titrated naive samples. Naive samples are compared to the primed sample from Figure 2. MEKi/ERKi titrated naive samples were sorted for R+G-/SUSD2+ expression.

(legend continued on next page)

post-implantation and pre-implantation embryos, respectively (Guo et al., 2014; Zhu et al., 2018). Global 5mCG levels were elevated by 8% in R+G− compared to R+G+ cells at P1, but this difference decreased in naive P5 and re-primed samples. When we plotted global 5mCG levels relative to the proportion of R+G+ cells, we detected a strong correlation in primed and naive samples (R^2 = 0.9928) (Figure 2G). As expected, the correlation was lost in re-primed cells since the reporter did not revert to silencing EGFP once activated. One could therefore use the R+G+ percentage to infer relative global methylation levels in primed and naive states. However, additional time points will need to be examined to discern the exact global methylation level during the early resetting process. In addition, these data are consistent with the notion that imprint erasure under naive conditions is a consequence of global DNA demethylation.

Finally, we examined global gene expression in R+G− vs. R+G+ naive cells through RNA sequencing (RNA-seq) on FACS-purified populations. There were no significant differentially expressed genes (DEGs) between the two groups at P1 of naive resetting and only two DEGs at P5: *FOS* and *FOSB* (Figure S2A). The FOS family of proteins are components of the activating protein 1 (AP-1) complex and have numerous functions in cell proliferation, survival, differentiation, and cancerous transformation (Hess et al., 2004). We closely examined the expression of markers of primed and naive pluripotency as well as epigenetic regulators, their complexes, targets, and products (collectively known as “epifactors”) (Medvedeva et al., 2015) (Figure S2B). Primed and naive samples showed robust expression of pluripotent-state-specific marker genes. However, there were no significant differences in the expression levels of epifactors between R+G− and R+G+ naive samples.

Modulating naive culture conditions to enhance imprint stability

Due to the current requirement for FGF pathway inhibition during primed-to-naive resetting (Khan et al., 2021), we sought to use our biallelic SNRPN reporter to assess the effect of reduced FGF pathway inhibition on naive identity and imprint stability. We first observed that removal of the ERK1/2 inhibitor (ERKi) GDC-0994 (hereafter called GDC) from the PXGGY/A cocktail prevented activation of the maternally methylated *SNRPN-EGFP* allele during resetting but also impaired activation of the naive marker SUSD2 (Figure 3A). We characterized these “no ERKi” cells by RNA-seq and found that they clustered between primed and naive cells by principal-component analysis (Figure 3B). Neither primed-specific markers (*ZIC2* and *SFRP1*) nor naive-specific markers (*KLF17* and *DNMT3L*) were upregulated in the no ERKi cells, although genes associated with gastrulation, such as *CER1* and *FGF17*, were upregulated only in no ERKi cells (Figure 3C). DEG analysis between no ERKi and naive PXGGY/A cells revealed a striking upregulation of genes related to neural development in no ERKi cells (Figures 3D and 3E). Gene set enrichment analysis highlighted relative enrichments for “epithelial-mesenchymal transition” in no ERKi cells and for “oxidative phosphorylation” in naive cells (Figures 3F and 3G). Since naive cells show morphological changes and increased oxygen consumption as they reset from the primed state (Dong et al., 2019; Gu et al., 2016), these results support the notion that no ERKi cells represent a cellular state between primed and naive pluripotency. These data indicate that removal of ERK inhibition prevents LOI but also fails to induce a *bona fide* naive pluripotent identity.

We then asked whether the onset of biallelic SNRPN reporter activity could be delayed by reducing the concentrations of FGF pathway inhibitors. To this end, we performed a dual titration of the MEK1/2 inhibitor (MEKi) PD0325901 (hereafter called PD03) and GDC during primed-to-naive resetting using our H9-SRG imprint reporter line. We tested concentrations of 0–1 μm MEKi (PD03) and 0–2.5 μm ERKi (GDC) and tracked reporter activity as well as SUSD2 expression for up to 27 days of naive resetting (Figures 4A and S3A). Overall, reduced MEK/ERK inhibition resulted in reduced activation of the maternally methylated *SNRPN-EGFP* reporter allele but also slower SUSD2 induction. We were particularly interested in identifying conditions that support the induction of the naive marker SUSD2 without concomitant activation of the *SNRPN*-EGFP reporter allele. Based on these data, we identified several samples that were simultaneously R+G− and SUSD2+, the phenotype of interest (quadrant highlighted in Figure 4A). Greater MEK/ERK inhibition caused cells to enter this imprinted-protected naive state earlier (day 7–12), but these conditions invariably transitioned to the R+G+ state at later time points. In contrast, lower MEK/ERK inhibition led to a slower, but more sustained, R+G−/SUSD2+ state.

We then explored to what extent R+G−/SUSD2+ cells under these modified conditions in fact maintained imprint methylation and possessed naive identity. We FACS-purified

(C) Summary of hyper-, hypo-, and properly methylated imprints based on MS-MLPA analysis.
(D) Principal-component analysis of RNA-seq data from primed and naive-titrated samples using two replicates. MEKi/ERKi titrated naive samples were sorted for R+G-/SUSD2+ expression.
(E) Marker gene expression of H9-SRG primed and naive samples by RNA-seq using two replicates.

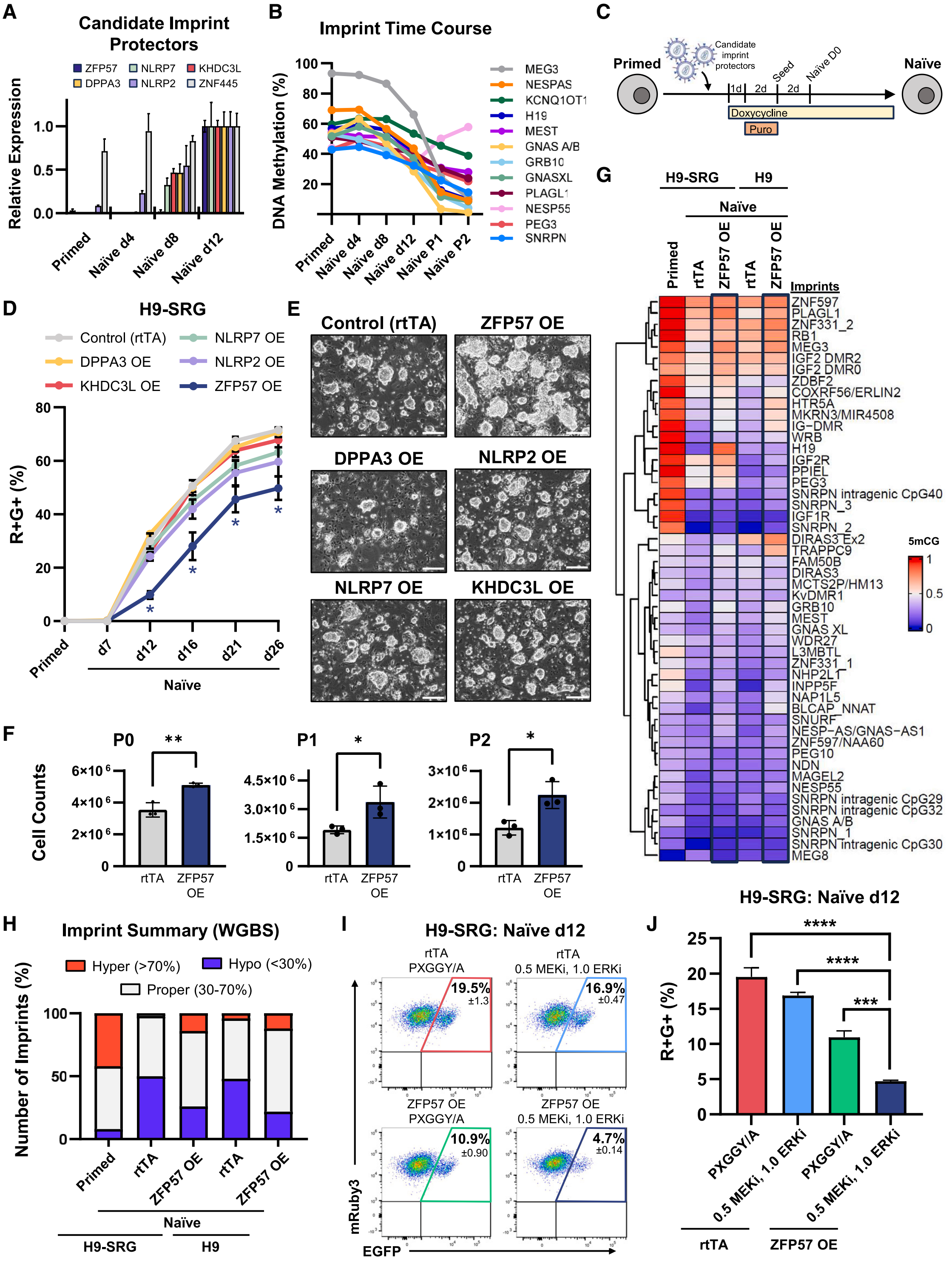

Figure 5. Overexpression of candidate imprint-protecting factors during primed-to-naive resetting

(A) Gene expression analysis of candidate factors in H9 hESCs by qPCR; $n = 2$ or 3 independent experiments.

(B) MS-MLPA analysis of DNA methylation at imprints during H9 primed-to-naive resetting. Imprints with multiple data points from the same locus were averaged for simplicity.

(legend continued on next page)

the samples "0.5 MEKi, 1.0 ERKi" and "1.0 MEKi, 2.5 ERKi" at day 7 and samples "0.5 MEKi, 0.5 ERKi" and "1.0 MEKi, 0.1 ERKi" at day 17 for R+G−/SUSD2+ markers. We then performed MS-MLPA and RNA-seq analysis to assess imprint methylation and naive identity. MS-MLPA analysis revealed that titrated naive samples maintained imprinted methylation better than the naive controls at similar time points (Figures 4B and 4C). Analysis of RNA-seq results indicated that all titrated samples reached the naive state as shown by principal-component analysis and marker gene expression (Figures 4D and 4E). Thus, these findings indicate that it is possible to acquire a *bona fide* naive identity in which imprints are retained under modified culture conditions, but this phenotype persists only transiently and in a subset of naive cells.

To test the effect of a short naive pulse followed by continued primed culture on imprint stability, we re-primed the sorted R+G−/SUSD2+ cells. WGBS analysis of the re-primed naive-titrated samples showed retention of methylation at imprints (Figure S3B). Additionally, for a subset of imprints, methylation shifted closer to 50% in cells that were re-primed after a brief naive pulse. Although subtle, there were improvements in the numbers of 30%–70% methylated loci and decreases in the numbers of hyper- and hypomethylated loci after re-priming. As an important caveat, however, these samples were not tested for allele-specific methylation. Therefore, further work will be required to determine whether transient naive treatment followed by re-priming may in fact modestly improve the fraction of imprints with the correct monoallelic methylation pattern.

Enhancing imprint stability by overexpressing candidate imprint-protecting factors

As an alternative approach, we investigated whether imprint stability could be enhanced by ectopic expression of candidate imprint-protecting factors. Studies in mice and humans have identified Dppa3/DPPA3, Zfp57/ZFP57, and Zfp445/ZNF445 as imprint-protecting factors during post-fertilization epigenetic reprogramming (Li et al., 2008; Mackay et al., 2008; Nakamura et al., 2007; Takahashi et al., 2019). Additionally, mutations in various components of the maternally deposited subcortical maternal complex, including *NLRP2*, *NLRP7*, and *KHDC3L*, are associated with molar pregnancy and widespread multi-locus imprinting disturbances (Monk et al., 2017). We asked whether these factors were present in our primed and naive hPSCs. qPCR analysis showed that all of these factors, except *ZNF445*, were lowly expressed in the primed state but were significantly upregulated in the naive state (Figure 5A). In contrast, *ZNF445* was robustly expressed throughout the primed-to-naive transition. Interestingly, however, we noticed that the expression of the other candidate imprint-protecting factors did not increase until day 8–12 of naive resetting. MS-MLPA analysis indicated that imprint methylation had already started to decrease during day 8–12, while some imprints exhibited demethylation at even earlier time points (Figure 5B). Based on these data, we hypothesized that candidate imprint-protecting factors (e.g., ZFP57, DPPA3, NLRP2, NLRP7, or KHDC3L) must be expressed from the beginning of primed-to-naive resetting in order to protect imprint methylation.

To examine this hypothesis, we used a doxycycline-inducible system to overexpress each factor individually in our H9-SRG imprint reporter line at the start of primed-to-naive resetting (Figure 5C). Successful overexpression of all transgenes was confirmed by qPCR (Figure S4A). While the level of transgenic ZFP57 appeared to be higher than that of the other overexpression factors, the endogenous levels of the other factors in naive hESCs were notably higher than that of ZFP57 (Figure S4B), thereby inflating the relative expression level of ZFP57

(C) Schematic for lentiviral transduction of candidate imprint-protecting factors in hPSCs. Doxycycline was added to activate transgene overexpression and puromycin resistance, and then cells were puromycin-selected for two days. Puromycin was removed, and cells were reset to the naive state under continued doxycycline treatment.

(D) Analysis of H9-SRG reporter activity by flow cytometry during primed-to-naive resetting of genetic overexpression samples. Error bars represent the standard error of the mean; $n = 3$ independent experiments. Significance comparisons reflect ZFP57 OE vs. control (rtTA). *Multiple unpaired t test q value < 0.05.

(E) Images of H9 naive cells overexpressing the indicated transgene. Scale bar, 200 μm.

(F) Cell counts of H9 rtTA and ZFP57 OE samples at the end of each passage during primed-to-naive resetting and maintenance. Samples were seeded at 250k cells/well and split at identical ratios after each passage. Error bars represent standard deviation; $n = 3$ independent experiments. *Unpaired t test p value < 0.05. **unpaired t test p value < 0.005.

(G) WGBS analysis of imprints in control and ZFP57 overexpression samples during naive resetting in PXGGY/A (H9-SRG, day 12) and 5i/L/A (H9, day 16). Naive samples were compared to the primed sample from Figure 2.

(H) Summary of hyper-, hypo-, and properly methylated imprints based on WGBS analysis.

(I) Flow cytometry data of H9-SRG cells with rtTA and/or ZFP57 transgenes at day 12 of primed-to-naive resetting in PXGGY/A or "0.5 MEKi, 1.0 ERKi" conditions. Plots are gated for SUSD2+ to show reporter activity of naive-converting cells.

(J) Analysis of the H9-SRG R+G+ population in Figure 5I. Error bars represent the standard deviation; $n = 3$ independent experiments. ***Unpaired t test p value < 0.0005. ****Unpaired t test p value < 0.0001.

transgene in the primed state. During resetting, ZFP57 overexpression led to delayed onset of biallelic SNRPN expression (Figure 5D), while overexpression of DPPA3, NLRP2, NLRP7, or KHDC3L did not have a significant effect. The vast majority of cells showed high SUSD2 surface marker expression in the naive state, suggesting that overexpression of candidate imprint-protecting factors did not compromise the acquisition of naive identity (Figure S4C). We then analyzed methylation levels at imprinted genes by MS-MLPA at days 12 and 16 of naive resetting. Consistent with delayed activation of the maternally imprinted SNRPN-EGFP allele, ZFP57 overexpression protected methylation levels at a subset of imprints (Figure S4D). However, methylation protection was not seen upon overexpression of the other candidate factors. We repeated this experiment using wild-type H9 hESCs (that do not contain the imprint reporter) using an alternative naive induction medium, 5i/L/A (Theunissen et al., 2014, 2016). Again, we observed a moderate protective effect of ZFP57 overexpression on imprint methylation (Figure S4D). These results demonstrate that the protective effect of ZFP57 is not limited to a specific culture system for inducing naive human pluripotency. Interestingly, the overexpression of ZFP57 also conferred a notable proliferation and/or survival advantage to naive cells derived in 5i/L/A (Figure 5E). Cell counts at three time points during naive resetting and maintenance showed a significant difference between rtTA and ZFP57 overexpression (OE) samples (Figure 5F). This effect may have been masked by the higher conversion efficiency in PXGGY/A (Khan et al., 2021).

To explore the imprint-protective effect of ZFP57 in more detail, we performed WGBS on H9-SRG control and ZFP57 overexpression samples at naive day 12 and day 16, as well as H9 control and ZFP57 overexpression at naive day 16. Nearly all imprints showed reduced methylation levels in naive control samples compared to the primed state (Figure 5G). Importantly, methylation levels were notably higher in ZFP57 overexpression samples at many imprints compared to the naive controls. Analysis of hyper-, hypo-, and properly methylated imprints indicated a substantial reduction in the number of hypomethylated imprints in naive ZFP57 overexpression compared to naive control samples (Figure 5H). Likewise, the number of properly methylated imprints increased with ZFP57 overexpression during primed-to-naive resetting. Substantial global DNA demethylation occurred in all naive samples compared to the primed state (Figure S4E). Global methylation levels were similar, although slightly elevated, in naive ZFP57 overexpression samples compared to naive controls. Importantly, however, ZFP57 upregulation did not affect the acquisition of naive pluripotency, as assessed by RNA-seq (Figure S4F). Principal-component analysis primarily separated between primed and naive states. While there was a small separation between the PXGGY/A and 5i/L/A samples, in accordance with our previous observations (Khan et al., 2021), rtTA and ZFP57 samples in the same conditions clustered together closely. We conclude that ZFP57 overexpression during primed-to-naive resetting provided imprint protection, potentially as a result of slower DNA demethylation, but did not interfere with naive identity.

We then asked whether combining the two imprint protection strategies (ZFP57 overexpression and MEKi/ERKi titration) would yield a greater effect than either strategy alone. Using our H9-SRG reporter line with and without ZFP57 overexpression, we tracked biallelic SNRPN activation during naive resetting in normal PXGGY/A medium and the titrated "0.5 MEKi, 1.0 ERKi" condition. Each strategy alone reduced biallelic SNRPN expression at day 12 of resetting, with an even greater decrease when both strategies were used together (Figures 5I and 5J). The naive marker SUSD2 was highly expressed in all samples, indicating that these imprint-protective strategies did not interfere with the acquisition of naive identity (Figure S4G).

Finally, based on the results obtained with ZFP57 overexpression, we considered whether other KZFP family genes might exhibit similar expression kinetics during primed-to-naive resetting and may therefore have a similar imprint-protective effect. To this end, we examined 25 KZFPs identified by the Monk group as binding factors to more than one imprinted region (Monteagudo-Sánchez et al., 2020). Using RNA-seq analysis of primed-to-naive intermediate cell populations reported by the Rugg-Gunn group (Collier et al., 2017), we identified three additional KZFPs (ZNF257, ZNF506, and ZNF534) that were significantly upregulated in naive compared to primed and intermediate cell populations (Figure S4H). Consistent with our data, ZFP57 also showed significant upregulation in the naive state, while ZNF445 showed consistent expression across primed, intermediate, and naive cell populations. The KZFPs ZNF257, ZNF506, and ZNF534 could be promising candidates for further investigation as candidate imprint-protecting factors to be expressed continuously during primed-to-naive resetting.

DISCUSSION

In the 10 years since naive hPSCs were first derived, they have become widely adopted in stem cell research, facilitating studies into basic mechanisms of early human development, the derivation of extraembryonic cell types, and the generation of stem-cell-based embryo models (reviewed in Dong et al., 2019; Zhou et al., 2023). However, a persistent issue hampering the application of naive hPSCs

is the erosion of parent-specific imprints under currently available culture regimes. In this study, we established a dual-colored fluorescent reporter cell line at the *SNRPN* locus (H9-SRG) to track the dynamics of imprint erasure during naive resetting at single-cell resolution. We showed that this reporter accurately reflects methylation at the *SNURF* ICR and correlates with 5mCG methylation globally. Using our H9-SRG imprint reporter as a cellular sensor for methylation levels, we evaluated two independent strategies to mitigate the LOI in naive hPSCs: titrating the concentrations of key kinase inhibitors or overexpressing candidate imprint-protecting factors. Our findings demonstrate that reducing the levels of MEK/ERK inhibition or overexpressing the KRAB zinc-finger protein ZFP57 can protect a subset of imprints during primed-to-naive resetting. Furthermore, combined MEK/ERK inhibitor titration and ZFP57 overexpression confer greater imprint protection than either strategy alone.

An important question when evaluating a live-cell reporter is to what extent the activity recorded from a single locus is representative of the genome-wide process of interest (in this case, imprinting). We opted to build an imprint reporter in the *SNRPN* locus because its ICR is known to undergo demethylation during primed-to-naive resetting, while the *SNRPN* transcript is robustly expressed across human pluripotent states (Pastor et al., 2016; Theunissen et al., 2016). Consistent with imprint erasure and a switch from mono- to biallelic SNRPN expression, the maternal *SNRPN-EGFP* allele became active in the majority of cells within 2–3 passages of naive resetting. As expected, these double-positive H9-SRG R+G+ cells displayed extensive DNA demethylation at the *SNRPN* locus and other imprinted loci. However, a subpopulation of naive cells retained monoallelic SNRPN expression (H9-SRG R+G−) and exhibited elevated DNA methylation at several ICRs at an early stage of primed-to-naive resetting (P1), but not at later passages (P5). The only ICR that consistently displayed elevated DNA methylation in H9-SRG R+G− naive cells was *SNURF*, which suggests that SNRPN reporter activity correlates most strongly with methylation of its nearest ICR. The H9-SRG reporter followed the genome-wide trend of ICR demethylation in the vast majority of cells (∼85% by P5 of naive resetting) and strongly correlated with global DNA methylation levels. Nevertheless, the H9-SRG reporter only shows partial correlation with other imprints, and the creation of an additional reporter, for example, one that targets a paternally imprinted locus, may allow for more sensitive detection of global LOI in the future.

We utilized the H9-SRG reporter to evaluate two independent strategies to preserve imprint integrity during primed-to-naive resetting. Based on our observation that omission of the ERK inhibitor GDC from the PXGGY/A cocktail causes primed hPSCs to enter an imprint-protected intermediate state, we assessed the impact of titrated levels of MEK and ERK inhibitors. Our results demonstrated that reducing MEK/ERK inhibition during naive resetting allows a subset of cells to enter a *bona fide* naive state with intact imprints. This population can be enriched using our H9-SRG reporter and sorting for R+G−/SUSD2+ cells. A small reduction in MEK/ERK inhibition supports an imprint-protected naive state by day 7 of naive treatment, but these cells have reduced imprints by day 12–17. However, a greater reduction of MEK/ERK inhibition supports a slower-to-attain but more sustained imprint-protected naive state. Both methods may be useful to the field depending on the experimental scenario. This approach is conceptually similar to that of Di Stefano and colleagues, who applied a reduced concentration of MEK inhibition to enhance genome stability during primed-to-naive resetting (Di Stefano et al., 2018).

As an alternative approach, we examined whether imprint stability could be enhanced by overexpressing candidate imprint-protecting factors during primed-to-naive resetting. Many biochemical factors appear to be important for imprint stability in humans (Monk et al., 2017; Monteagudo-Sánchez et al., 2020; Takahashi et al., 2019). Here, we demonstrated that, while several candidate imprint-protecting proteins may be important during early embryogenesis, of those tested, only overexpression of ZFP57 could protect a subset of imprints during primed-to-naive resetting. These results were reproduced using two independent naive induction cocktails, PXGGY/A (Khan et al., 2021) and 5i/L/A (Theunissen et al., 2014). In case of the latter, ZFP57 also conferred a growth/survival advantage during naive resetting. This method of using a transgene to help protect imprinting is a significant step toward more complete imprint protection. We postulate that a transgenic approach may allow for further elucidation of imprint-protective mechanisms and facilitate the development of non-transgenic methods to enhance the epigenetic stability of naive hPSCs.

Landmark studies in mouse ESCs demonstrated that germline transmission is required for establishment of monoallelic methylation and expression patterns of imprinted genes (Tucker et al., 1996). Consistent with these observations, we and others previously reported that imprints do not recover upon differentiation of naive hPSCs (Pastor et al., 2016; Theunissen et al., 2016). We FACS-purified H9-SRG cells at P1 and P5 of primed-to-naive resetting into R+G−/R+G+ populations and immediately returned them to primed culture conditions. While the sorted R+G+ cells maintained high EGFP percentages, R+G− cells maintained their EGFP− status throughout re-priming. These data confirm that imprint erasure is irreversible during primed-to-naive resetting and that imprinted *SNRPN*

expression can be preserved by returning EGFP− naive cells to primed culture conditions before LOI has occurred. Intriguingly, R+G− cells that were maintained for 5 passages in naive media and then returned to primed conditions also exhibited enhanced DNA methylation at several ICRs outside the *SNRPN* locus, which raises the possibility that some parent-specific memory may be retained independently of DNA methylation.

While most imprints are properly methylated in the primed state, our results indicate that there are numerous exceptions where imprints are hyper- or hypomethylated in primed hPSCs. Similar to the recent work by Buckberry et al., we asked whether a short pulse of naive treatment could improve the epigenetic state of primed hPSCs (Buckberry et al., 2023). Our re-priming of H9-SRG reporter cells following a short naive treatment with reduced MEK/ERK inhibition showed that methylation levels at imprints can be retained when not erased in the naive state. However, allele-specific methylation data will be needed to determine the full extent of imprint preservation and whether transient naive treatment followed by re-priming may in fact lead to a modest improvement in the fraction of properly imprinted imprints at the expense of hyper- and hypomethylated imprints.

Since ZFP57 is expressed in human blastocysts (Takahashi et al., 2019), one might ask whether blastocyst-derived naive hESCs retain proper imprinted methylation. Established HNES1 cells in t2iLGö(Y) medium were still found to have largely hypomethylated imprints (Guo et al., 2016, 2017). This suggests, in agreement with our data (Figure 5D), that naive culture conditions still erode imprints over time even in the presence of ZFP57. It will be of interest to investigate whether imprints are better preserved in blastocyst-derived naive hESCs at early passages and delineate the imprint-protective effects of ZFP57 and other factors in this context. Additional factors of interest include ZNF257, ZNF506, and ZNF534, which were identified as having imprint-binding capacity (Monteagudo-Sánchez et al., 2020) and showed similar expression dynamics as ZFP57 during primed-to-naive resetting (Collier et al., 2017). Overall, the findings in this study provide an important step toward improving the imprint fidelity of naive hPSCs and their applications for studies of human development and regeneration.

METHODS

Ethics statement

All experiments involving hESCs were approved by the Institutional Biological and Chemical Safety Committee and Embryonic Stem Cell Research Oversight Committee at Washington University School of Medicine.

hPSC culture

Primed hPSCs were cultured on Matrigel in mTeSR Plus media at 37°C, 20% O_2, and 5% CO_2. Naive hPSCs were cultured on mouse embryonic fibroblasts in their respective media at 37°C, 5% O_2, and 5% CO_2. Mycoplasma testing was performed routinely, and all samples were negative. Cultures were consistently monitored and were negative for bacteria and fungus. See supplemental methods for more details.

Gene editing to establish the SNRPN dual-colored reporter

H9 primed hESCs were nucleofected with sgRNA, Cas9 protein, and donor plasmids using a Lonza 4-D nucleofector system. Single-cell clones were obtained by FACS and expanded in culture. Inserts were validated by junction PCR. See supplemental methods for more details.

Flow cytometry/FACS

Flow cytometry was performed on a Sony SY3200 Synergy cytometer (FACS purification) or a Beckman Coulter CytoFLEX S cytometer (analysis). See supplemental methods for more details.

MS-MLPA assay

MS-MLPA assays were performed using reagents from MRC Holland multi-locus imprinting kits ME034-B1 or ME034-C1 according to manufacturer's instructions (Nygren et al., 2005). See supplemental methods for more details.

RNA isolation, cDNA synthesis, and qPCR

Total RNA was isolated using the RNeasy mini kit (QIAGEN, 74104) with DNase I treatment (Omega, E1091) per manufacturer's instructions. Total RNA was reverse-transcribed using high-capacity cDNA reverse transcription reagents (Applied Biosystems, 4368814). cDNA was diluted 1:20 in molecular-grade water. qPCR reactions were performed with PowerUp SYBR Green master mix (Applied Biosystems, A25743) on an Applied Biosystems StepOnePlus or QuantStudio 3 real-time PCR system. Oligos are listed in Table S1. Gene expression values were normalized to the housekeeping gene *RPLP0*. Error bars represent the standard deviation. Plots were visualized with GraphPad Prism 10.1.2.

Bulk RNA-seq and analysis

Samples were sequenced on an Illumina NovaSeq 6000 or NovaSeq X Plus using paired end reads of 150 bases. Basecalls and demultiplexing were performed with Illumina's bcl2fastq software and a custom python program with a maximum of one mismatch in the indexing read. See supplemental methods for more details.

WGBS analysis

Bisulfite conversion was performed on genomic DNA using the EZ DNA Methylation-Gold kit (Zymo Research, D5005). Library preparation was performed manually using reagents from the Accel-NGS Methyl-Seq DNA library kit (Swift BioSciences, 30024). Libraries were sequenced on an Illumina NovaSeq 6000 (Figure 2) or NovaSeq X Plus (Figures 5 and S3) flow cell using 300 cycles. See supplemental methods for bioinformatic processing.

RESOURCE AVAILABILITY

Lead contact

Requests for further information and resources should be directed to and will be fulfilled by the lead contact, Thorold W. Theunissen (t.theunissen@wustl.edu).

Materials availability

Materials are available upon reasonable request to the lead contact.

Data and code availability

Raw and processed data for WGBS and RNA-seq experiments generated in this study have been deposited in the NCBI's Gene Expression Omnibus and are available under GSE268536 and GSE268535, respectively.

ACKNOWLEDGMENTS

We thank Malkiel Cohen and Rudolf Jaenisch for sharing the inducible lentiviral overexpression vector. We thank the Genome Engineering & Stem Cell Center at The McDonnell Genome Institute of Washington University for assistance with constructing CRISPR-Cas9 knockins, the Cytogenetics and Molecular Pathology Laboratory in the Department of Pathology and Immunology for G-banded karyotyping, and the Genome Technology Access Center at The McDonnell Genome Institute for assistance with bulk RNA-seq and WGBS. For scientific input and manuscript feedback, we thank John R. Edwards, Kristen L. Kroll, Tim Schedl, Eric L. Greer, and members of the Theunissen Lab.

This work was supported by the NIH Director's New Innovator Award (DP2GM137418); the NIGMS Maximizing Investigator's Research Award (R35GM153439-01); and grants from the Shipley Foundation Program for Innovation in Stem Cell Science, the Edward Mallinckrodt, Jr. Foundation Grant, and Washington University Children's Discovery Institute to T.W.T. Additional support was provided by the NIDDK (R01DK046718) to H.J. as well as an NIH CMB Training Grant (T32GM007067) and a Douglas Covey Graduate Student Fellowship to L.A.F.

AUTHOR CONTRIBUTIONS

L.A.F., J.E.Z., B.M., and K.-m.P. performed and assisted with experiments under the supervision of T.W.T. M.R. performed and analyzed MS-MLPA experiments under the supervision of H.J. on samples provided by L.A.F. Bulk RNA-seq and WGBS were analyzed by L.A.F. and J.K.H. under the supervision of S.D., T.W., and T.W.T. L.A.F. and T.W.T. wrote the manuscript with input from others.

DECLARATION OF INTERESTS

T.W.T. is a member of the Early Career Editorial Board at Stem Cell Reports and a consultant for Stately Bio, Inc. L.A.F. and T.W.T. are co-inventors on a patent application related to tracking and mitigating imprint stability in naive hPSCs.

SUPPLEMENTAL INFORMATION

Supplemental information can be found online at https://doi.org/10.1016/j.stemcr.2025.102419.

Received: June 19, 2024
Revised: January 14, 2025
Accepted: January 15, 2025
Published: February 13, 2025

REFERENCES

An, C., Feng, G., Zhang, J., Cao, S., Wang, Y., Wang, N., Lu, F., Zhou, Q., and Wang, H. (2020). Overcoming Autocrine FGF Signaling-Induced Heterogeneity in Naive Human ESCs Enables Modeling of Random X Chromosome Inactivation. Cell Stem Cell *27*, 482–497.e4. https://doi.org/10.1016/j.stem.2020.06.002.

Bar, S., Schachter, M., Eldar-Geva, T., and Benvenisty, N. (2017). Large-Scale Analysis of Loss of Imprinting in Human Pluripotent Stem Cells. Cell Rep. *19*, 957–968. https://doi.org/10.1016/j.celrep.2017.04.020.

Barton, S.C., Surani, M.A., and Norris, M.L. (1984). Role of paternal and maternal genomes in mouse development. Nature *311*, 374–376. https://doi.org/10.1038/311374a0.

Buckberry, S., Liu, X., Poppe, D., Tan, J.P., Sun, G., Chen, J., Nguyen, T.V., de Mendoza, A., Pflueger, J., Frazer, T., et al. (2023). Transient naive reprogramming corrects hiPS cells functionally and epigenetically. Nature *620*, 863–872. https://doi.org/10.1038/s41586-023-06424-7.

Castel, G., Meistermann, D., Bretin, B., Firmin, J., Blin, J., Loubersac, S., Bruneau, A., Chevolleau, S., Kilens, S., Chariau, C., et al. (2020). Induction of Human Trophoblast Stem Cells from Somatic Cells and Pluripotent Stem Cells. Cell Rep. *33*, 108419. https://doi.org/10.1016/j.celrep.2020.108419.

Cinkornpumin, J.K., Kwon, S.Y., Guo, Y., Hossain, I., Sirois, J., Russett, C.S., Tseng, H.W., Okae, H., Arima, T., Duchaine, T.F., et al. (2020). Naive Human Embryonic Stem Cells Can Give Rise to Cells with a Trophoblast-like Transcriptome and Methylome. Stem Cell Rep. *15*, 198–213. https://doi.org/10.1016/j.stemcr.2020.06.003.

Collier, A.J., Panula, S.P., Schell, J.P., Chovanec, P., Plaza Reyes, A., Petropoulos, S., Corcoran, A.E., Walker, R., Douagi, I., Lanner, F., and Rugg-Gunn, P.J. (2017). Comprehensive Cell Surface Protein Profiling Identifies Specific Markers of Human Naive and Primed Pluripotent States. Cell Stem Cell *20*, 874–890.e7. https://doi.org/10.1016/j.stem.2017.02.014.

Court, F., Tayama, C., Romanelli, V., Martin-Trujillo, A., Iglesias-Platas, I., Okamura, K., Sugahara, N., Simón, C., Moore, H., Harness, J.V., et al. (2014). Genome-wide parent-of-origin DNA methylation analysis reveals the intricacies of human imprinting and suggests a germline methylation-independent mechanism of

establishment. Genome Res. *24*, 554–569. https://doi.org/10.1101/gr.164913.113.

Dattani, A., Corujo-Simon, E., Radley, A., Heydari, T., Taheriabkenar, Y., Carlisle, F., Lin, S., Liddle, C., Mill, J., Zandstra, P.W., et al. (2024). Naive pluripotent stem cell-based models capture FGF-dependent human hypoblast lineage specification. Cell Stem Cell *31*, 1058–1071.e5. https://doi.org/10.1016/j.stem.2024.05.003.

Di Stefano, B., Ueda, M., Sabri, S., Brumbaugh, J., Huebner, A.J., Sahakyan, A., Clement, K., Clowers, K.J., Erickson, A.R., Shioda, K., et al. (2018). Reduced MEK inhibition preserves genomic stability in naive human embryonic stem cells. Nat. Methods *15*, 732–740. https://doi.org/10.1038/s41592-018-0104-1.

Dong, C., Beltcheva, M., Gontarz, P., Zhang, B., Popli, P., Fischer, L.A., Khan, S.A., Park, K.-m., Yoon, E.-J., Xing, X., et al. (2020). Derivation of trophoblast stem cells from naïve human pluripotent stem cells. Elife *9*, e52504. https://doi.org/10.7554/eLife.52504.

Dong, C., Fischer, L.A., and Theunissen, T.W. (2019). Recent insights into the naïve state of human pluripotency and its applications. Exp. Cell Res. *385*, 111645. https://doi.org/10.1016/j.yexcr.2019.111645.

Dror, I., Chitiashvili, T., Tan, S.Y.X., Cano, C.T., Sahakyan, A., Markaki, Y., Chronis, C., Collier, A.J., Deng, W., Liang, G., et al. (2024). XIST directly regulates X-linked and autosomal genes in naive human pluripotent cells. Cell *187*, 110–129.e31. https://doi.org/10.1016/j.cell.2023.11.033.

Gu, W., Gaeta, X., Sahakyan, A., Chan, A.B., Hong, C.S., Kim, R., Braas, D., Plath, K., Lowry, W.E., and Christofk, H.R. (2016). Glycolytic Metabolism Plays a Functional Role in Regulating Human Pluripotent Stem Cell State. Cell Stem Cell *19*, 476–490. https://doi.org/10.1016/j.stem.2016.08.008.

Guo, G., Stirparo, G.G., Strawbridge, S.E., Spindlow, D., Yang, J., Clarke, J., Dattani, A., Yanagida, A., Li, M.A., Myers, S., et al. (2021). Human naive epiblast cells possess unrestricted lineage potential. Cell Stem Cell *28*, 1040–1056.e6. https://doi.org/10.1016/j.stem.2021.02.025.

Guo, G., von Meyenn, F., Rostovskaya, M., Clarke, J., Dietmann, S., Baker, D., Sahakyan, A., Myers, S., Bertone, P., Reik, W., et al. (2017). Epigenetic resetting of human pluripotency. Development *144*, 2748–2763. https://doi.org/10.1242/dev.146811.

Guo, G., von Meyenn, F., Santos, F., Chen, Y., Reik, W., Bertone, P., Smith, A., and Nichols, J. (2016). Naive Pluripotent Stem Cells Derived Directly from Isolated Cells of the Human Inner Cell Mass. Stem Cell Rep. *6*, 437–446. https://doi.org/10.1016/j.stemcr.2016.02.005.

Guo, H., Zhu, P., Yan, L., Li, R., Hu, B., Lian, Y., Yan, J., Ren, X., Lin, S., Li, J., et al. (2014). The DNA methylation landscape of human early embryos. Nature *511*, 606–610. https://doi.org/10.1038/nature13544.

Hess, J., Angel, P., and Schorpp-Kistner, M. (2004). AP-1 subunits: quarrel and harmony among siblings. J. Cell Sci. *117*, 5965–5973. https://doi.org/10.1242/jcs.01589.

Io, S., Kabata, M., Iemura, Y., Semi, K., Morone, N., Minagawa, A., Wang, B., Okamoto, I., Nakamura, T., Kojima, Y., et al. (2021). Capturing human trophoblast development with naive pluripotent stem cells in vitro. Cell Stem Cell *28*, 1023–1039. https://doi.org/10.1016/j.stem.2021.03.013.

Kalish, J.M., Jiang, C., and Bartolomei, M.S. (2014). Epigenetics and imprinting in human disease. Int. J. Dev. Biol. *58*, 291–298. https://doi.org/10.1387/ijdb.140077mb.

Keshet, G., and Benvenisty, N. (2021). Large-scale analysis of imprinting in naive human pluripotent stem cells reveals recurrent aberrations and a potential link to FGF signaling. Stem Cell Rep. *16*, 2520–2533. https://doi.org/10.1016/j.stemcr.2021.09.002.

Khan, S.A., Park, K.-m., Fischer, L.A., Dong, C., Lungjangwa, T., Jimenez, M., Casalena, D., Chew, B., Dietmann, S., Auld, D.S., et al. (2021). Probing the signaling requirements for naive human pluripotency by high-throughput chemical screening. Cell Rep. *35*, 109233. https://doi.org/10.1016/j.celrep.2021.109233.

Li, X., Ito, M., Zhou, F., Youngson, N., Zuo, X., Leder, P., and Ferguson-Smith, A.C. (2008). A maternal-zygotic effect gene, Zfp57, maintains both maternal and paternal imprints. Dev. Cell *15*, 547–557. https://doi.org/10.1016/j.devcel.2008.08.014.

Li, Z.K., Wang, L.Y., Wang, L.B., Feng, G.H., Yuan, X.W., Liu, C., Xu, K., Li, Y.H., Wan, H.F., Zhang, Y., et al. (2018). Generation of Bimaternal and Bipaternal Mice from Hypomethylated Haploid ESCs with Imprinting Region Deletions. Cell Stem Cell *23*, 665–676.e4. https://doi.org/10.1016/j.stem.2018.09.004.

Liao, Z., Zhang, J., Sun, S., Li, Y., Xu, Y., Li, C., Cao, J., Nie, Y., Niu, Z., Liu, J., et al. (2024). Reprogramming mechanism dissection and trophoblast replacement application in monkey somatic cell nuclear transfer. Nat. Commun. *15*, 5. https://doi.org/10.1038/s41467-023-43985-7.

Mackay, D.J.G., Callaway, J.L.A., Marks, S.M., White, H.E., Acerini, C.L., Boonen, S.E., Dayanikli, P., Firth, H.V., Goodship, J.A., Haemers, A.P., et al. (2008). Hypomethylation of multiple imprinted loci in individuals with transient neonatal diabetes is associated with mutations in ZFP57. Nat. Genet. *40*, 949–951. https://doi.org/10.1038/ng.187.

McGrath, J., and Solter, D. (1984). Completion of mouse embryogenesis requires both the maternal and paternal genomes. Cell *37*, 179–183. https://doi.org/10.1016/0092-8674(84)90313-1.

Medvedeva, Y.A., Lennartsson, A., Ehsani, R., Kulakovskiy, I.V., Vorontsov, I.E., Panahandeh, P., Khimulya, G., Kasukawa, T., and FANTOM Consortium, and Drabløs, F. (2015). EpiFactors: a comprehensive database of human epigenetic factors and complexes. Database *2015*, bav067. https://doi.org/10.1093/database/bav067.

Monk, D., Mackay, D.J.G., Eggermann, T., Maher, E.R., and Riccio, A. (2019). Genomic imprinting disorders: lessons on how genome, epigenome and environment interact. Nat. Rev. Genet. *20*, 235–248. https://doi.org/10.1038/s41576-018-0092-0.

Monk, D., Sanchez-Delgado, M., and Fisher, R. (2017). NLRPs, the subcortical maternal complex and genomic imprinting. Reproduction *154*, R161–R170. https://doi.org/10.1530/REP-17-0465.

Monteagudo-Sánchez, A., Hernandez Mora, J.R., Simon, C., Burton, A., Tenorio, J., Lapunzina, P., Clark, S., Esteller, M., Kelsey, G., López-Siguero, J.P., et al. (2020). The role of ZFP57 and additional KRAB-zinc finger proteins in the maintenance of

human imprinted methylation and multi-locus imprinting disturbances. Nucleic Acids Res. *48*, 11394–11407. https://doi.org/10.1093/nar/gkaa837.

Nakamura, T., Arai, Y., Umehara, H., Masuhara, M., Kimura, T., Taniguchi, H., Sekimoto, T., Ikawa, M., Yoneda, Y., Okabe, M., et al. (2007). PGC7/Stella protects against DNA demethylation in early embryogenesis. Nat. Cell Biol. *9*, 64–71. https://doi.org/10.1038/ncb1519.

Nichols, J., and Smith, A. (2009). Naive and Primed Pluripotent States. Cell Stem Cell *4*, 487–492. https://doi.org/10.1016/j.stem.2009.05.015.

Nygren, A.O.H., Ameziane, N., Duarte, H.M.B., Vijzelaar, R.N.C.P., Waisfisz, Q., Hess, C.J., Schouten, J.P., and Errami, A. (2005). Methylation-Specific MLPA (MS-MLPA): simultaneous detection of CpG methylation and copy number changes of up to 40 sequences. Nucleic Acids Res. *33*, e128. https://doi.org/10.1093/nar/gni127.

Okubo, T., Rivron, N., Kabata, M., Masaki, H., Kishimoto, K., Semi, K., Nakajima-Koyama, M., Kunitomi, H., Kaswandy, B., Sato, H., et al. (2024). Hypoblast from human pluripotent stem cells regulates epiblast development. Nature *626*, 357–366. https://doi.org/10.1038/s41586-023-06871-2.

Pastor, W.A., Chen, D., Liu, W., Kim, R., Sahakyan, A., Lukianchikov, A., Plath, K., Jacobsen, S.E., and Clark, A.T. (2016). Naive Human Pluripotent Cells Feature a Methylation Landscape Devoid of Blastocyst or Germline Memory. Cell Stem Cell *18*, 323–329. https://doi.org/10.1016/j.stem.2016.01.019.

Peters, J. (2014). The role of genomic imprinting in biology and disease: an expanding view. Nat. Rev. Genet. *15*, 517–530. https://doi.org/10.1038/nrg3766.

Rugg-Gunn, P.J., Ferguson-Smith, A.C., and Pedersen, R.A. (2007). Status of genomic imprinting in human embryonic stem cells as revealed by a large cohort of independently derived and maintained lines. Hum. Mol. Genet. *16 Spec No. 2*, R243–R251. https://doi.org/10.1093/hmg/ddm245.

Sahakyan, A., Kim, R., Chronis, C., Sabri, S., Bonora, G., Theunissen, T.W., Kuoy, E., Langerman, J., Clark, A.T., Jaenisch, R., and Plath, K. (2017). Human Naive Pluripotent Stem Cells Model X Chromosome Dampening and X Inactivation. Cell Stem Cell *20*, 87–101. https://doi.org/10.1016/j.stem.2016.10.006.

Stelzer, Y., Shivalila, C.S., Soldner, F., Markoulaki, S., and Jaenisch, R. (2015). Tracing Dynamic Changes of DNA Methylation at Single-Cell Resolution. Cell *163*, 218–229. https://doi.org/10.1016/j.cell.2015.08.046.

Surani, M.A., Barton, S.C., and Norris, M.L. (1984). Development of reconstituted mouse eggs suggests imprinting of the genome during gametogenesis. Nature *308*, 548–550. https://doi.org/10.1038/308548a0.

Takahashi, N., Coluccio, A., Thorball, C.W., Planet, E., Shi, H., Offner, S., Turelli, P., Imbeault, M., Ferguson-Smith, A.C., and Trono, D. (2019). ZNF445 is a primary regulator of genomic imprinting. Genes Dev. *33*, 49–54.

Takashima, Y., Guo, G., Loos, R., Nichols, J., Ficz, G., Krueger, F., Oxley, D., Santos, F., Clarke, J., Mansfield, W., et al. (2014). Resetting transcription factor control circuitry toward ground-state pluripotency in human. Cell *158*, 1254–1269. https://doi.org/10.1016/j.cell.2014.08.029.

Theunissen, T.W., Friedli, M., He, Y., Planet, E., O'Neil, R.C., Markoulaki, S., Pontis, J., Wang, H., Iouranova, A., Imbeault, M., et al. (2016). Molecular Criteria for Defining the Naive Human Pluripotent State. Cell Stem Cell *19*, 502–515. https://doi.org/10.1016/j.stem.2016.06.011.

Theunissen, T.W., Powell, B.E., Wang, H., Mitalipova, M., Faddah, D.A., Reddy, J., Fan, Z.P., Maetzel, D., Ganz, K., Shi, L., et al. (2014). Systematic identification of culture conditions for induction and maintenance of naive human pluripotency. Cell Stem Cell *15*, 471–487. https://doi.org/10.1016/j.stem.2014.07.002.

Tucci, V., Isles, A.R., Kelsey, G., and Ferguson-Smith, A.C.; Erice Imprinting Group (2019). Genomic Imprinting and Physiological Processes in Mammals. Cell *176*, 952–965. https://doi.org/10.1016/j.cell.2019.01.043.

Tucker, K.L., Beard, C., Dausmann, J., Jackson-Grusby, L., Laird, P.W., Lei, H., Li, E., and Jaenisch, R. (1996). Germ-line passage is required for establishment of methylation and expression patterns of imprinted but not of nonimprinted genes. Genes Dev. *10*, 1008–1020. https://doi.org/10.1101/gad.10.8.1008.

Wilkins, J.F., and Haig, D. (2003). What good is genomic imprinting: the function of parent-specific gene expression. Nat. Rev. Genet. *4*, 359–368. https://doi.org/10.1038/nrg1062.

Zhou, J., Hu, J., Wang, Y., and Gao, S. (2023). Induction and application of human naive pluripotency. Cell Rep. *42*, 112379. https://doi.org/10.1016/j.celrep.2023.112379.

Zhu, P., Guo, H., Ren, Y., Hou, Y., Dong, J., Li, R., Lian, Y., Fan, X., Hu, B., Gao, Y., et al. (2018). Single-cell DNA methylome sequencing of human preimplantation embryos. Nat. Genet. *50*, 12–19. https://doi.org/10.1038/s41588-017-0007-6.

Stem Cell Reports, Volume *20*

Supplemental Information

Tracking and mitigating imprint erasure during induction of naive human pluripotency at single-cell resolution

Laura A. Fischer, Brittany Meyer, Monica Reyes, Joseph E. Zemke, Jessica K. Harrison, Kyoung-mi Park, Ting Wang, Harald Jüppner, Sabine Dietmann, and Thorold W. Theunissen

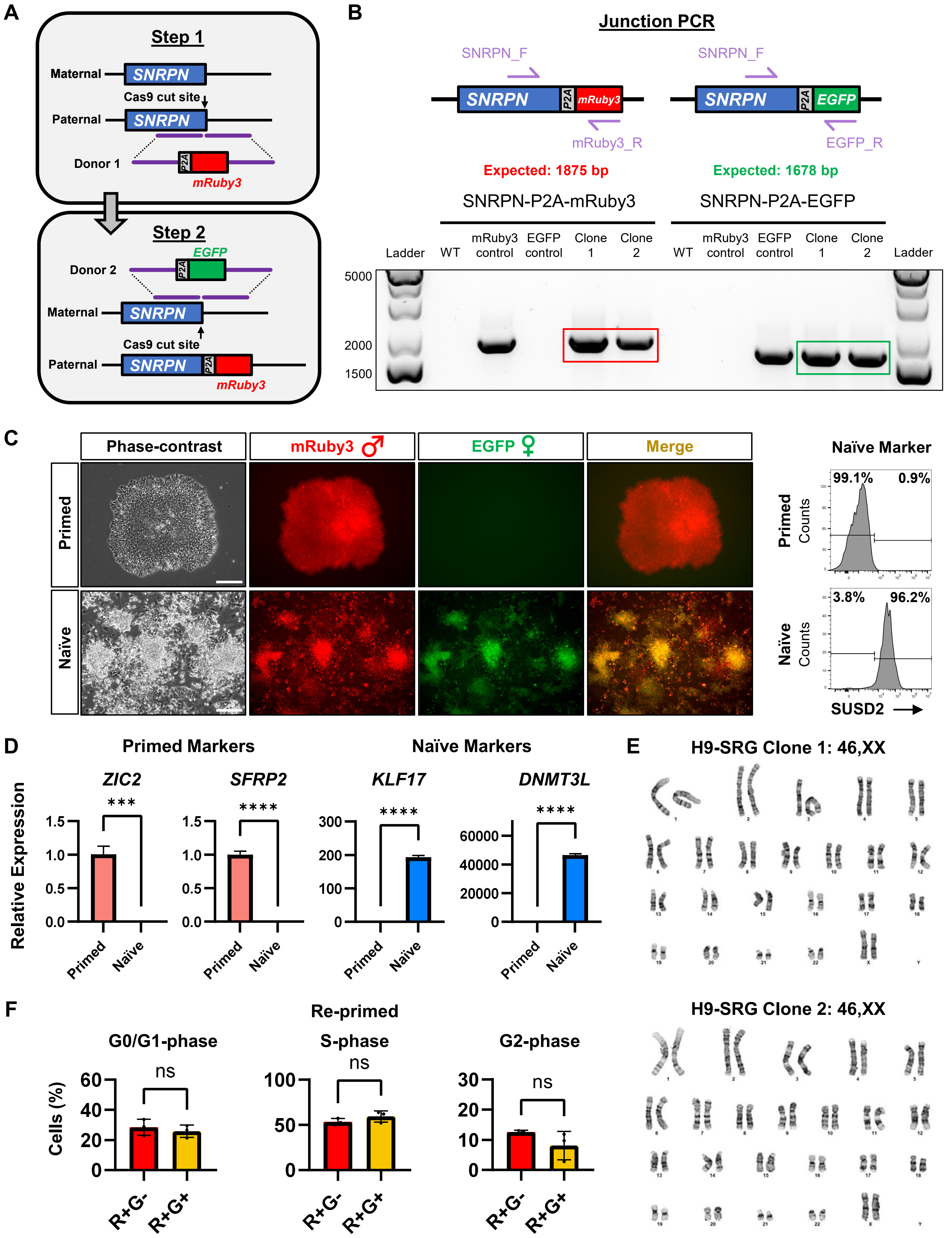
A
Step 1
Maternal
SNRPN
Cas9 cut site
Paternal
SNRPN
Donor 1
P2A
mRuby3
Step 2
EGFP
Donor 2
P2A
Maternal
SNRPN
Cas9 cut site
Paternal
SNRPN
P2A
mRuby3
B
Junction PCR
SNRPN_F
SNRPN
P2A
mRuby3
mRuby3_R
SNRPN_F
SNRPN
P2A
EGFP
EGFP_R
Expected: 1875 bp
Expected: 1678 bp
SNRPN-P2A-mRuby3
SNRPN-P2A-EGFP
Ladder
WT
mRuby3 control
EGFP control
Clone 1
Clone 2
WT
mRuby3 control
EGFP control
Clone 1
Clone 2
Ladder
5000
2000
1500
C
Phase-contrast
mRuby3 ♂
EGFP ♀
Merge
Primed
Naïve
Naïve Marker
Primed
Counts
99.1%
0.9%
Naïve
Counts
3.8%
96.2%
SUSD2
D
Primed Markers
Naïve Markers
Relative Expression
ZIC2
SFRP2
KLF17
DNMT3L
***
****
****
****
1.5
1.0
0.5
0.0
300
200
100
0
60000
40000
20000
0
Primed
Naïve
E
H9-SRG Clone 1: 46,XX
H9-SRG Clone 2: 46,XX
F
Re-primed
G0/G1-phase
S-phase
G2-phase
Cells (%)
ns
ns
ns
60
40
20
0
100
50
0
30
20
10
0
R+G-
R+G+

Figure S1

Figure S1: Constructing and characterizing the H9-SRG dual-colored reporter [Related to Figure 1]

- **A.** Creation of the H9-SRG reporter cell line. First, *P2A-mRuby3* was integrated by CRISPR/Cas9 editing into the active, paternal *SNRPN* allele. Single-cell clones were isolated by FACS. Second, *P2A-EGFP* was integrated by CRISPR/Cas9 editing into the silent, maternal *SNRPN* allele. Single-cell clones were isolated and later screened by junction PCR.
- **B.** Validation of *P2A-mRuby3* and *P2A-EGFP* integration at the *SNRPN* locus by junction PCR. "WT" = H9 primed. "mRuby3 control" has only mRuby3 integrated. "EGFP control" has only EGFP integrated. "Clone 1" and "Clone 2" each have an mRuby3 and an EGFP integration and were used throughout this study.
- **C.** Images and flow cytometry data for H9-SRG cells (Clone 2) in the primed and naïve states. Scale bar = 200 μm. Images are representative of four time points. See Figure 1B for Clone 1.
- **D.** Relative expression of primed and naïve marker genes in H9-SRG cells (Clone 1) by qPCR. All genes are displayed with Primed = 1.0. Error bars represent standard deviation; n = 3 independent experiments. ***unpaired t-test p-value = 0.0001. ****unpaired t-test p-value < 0.0001.
- **E.** Karyotype analysis of H9-SRG Clones 1 and 2.
- **F.** Cell cycle analysis of re-primed R+G- and R+G+ populations that had been sorted at naïve P1. Error bars represent standard deviation; n = 3 independent experiments. "ns" represents unpaired t-test p-value > 0.05.

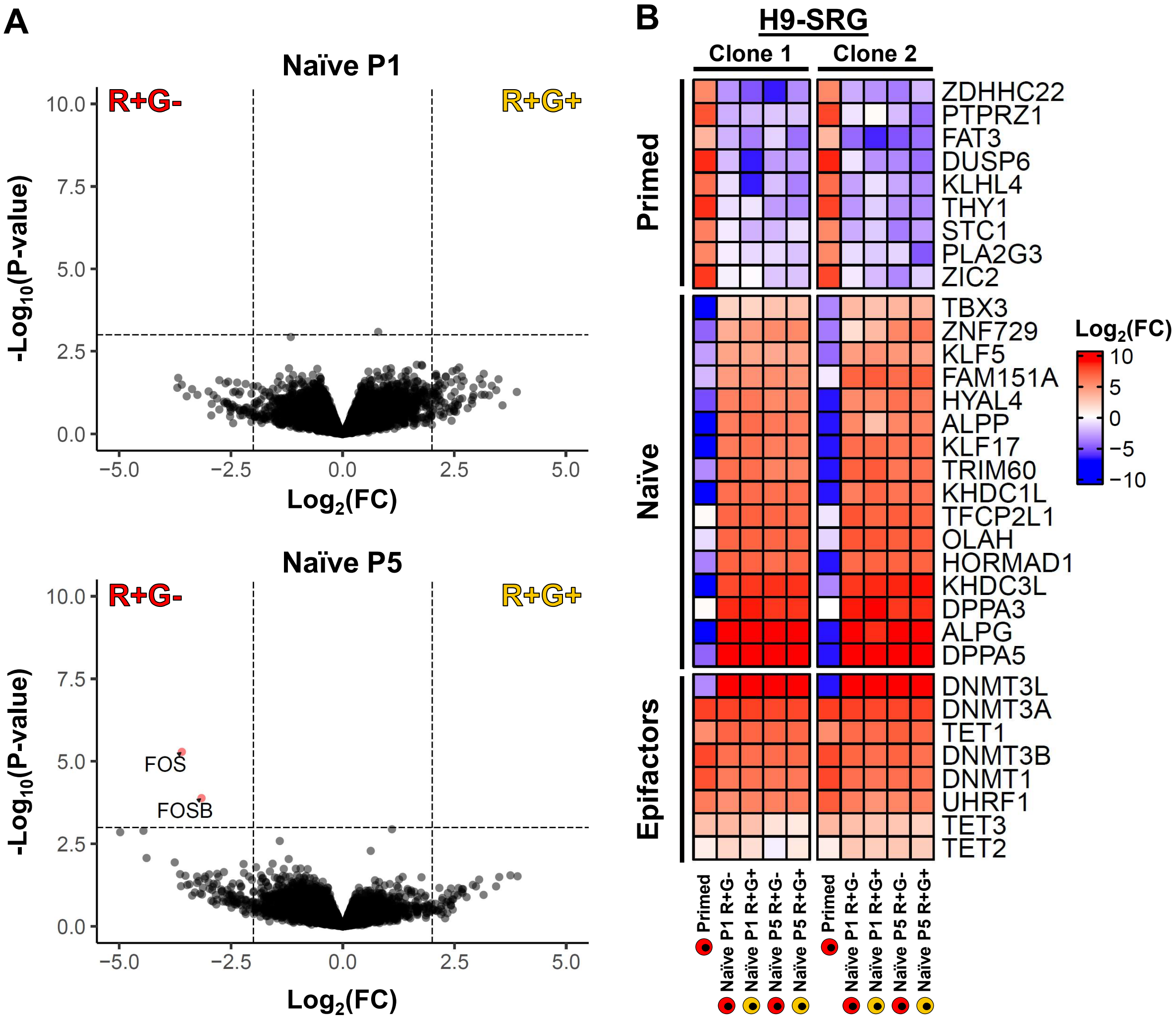
A
Naïve P1
R+G-
R+G+
-Log10(P-value)
Log2(FC)
Naïve P5
FOS
FOSB
B
H9-SRG
Clone 1
Clone 2
Primed
Naïve
Epifactors
ZDHHC22
PTPRZ1
FAT3
DUSP6
KLHL4
THY1
STC1
PLA2G3
ZIC2
TBX3
ZNF729
KLF5
FAM151A
HYAL4
ALPP
KLF17
TRIM60
KHDC1L
TFCP2L1
OLAH
HORMAD1
KHDC3L
DPPA3
ALPG
DPPA5
DNMT3L
DNMT3A
TET1
DNMT3B
DNMT1
UHRF1
TET3
TET2
Log2(FC)
Primed
Naïve P1 R+G-
Naïve P1 R+G+
Naïve P5 R+G-
Naïve P5 R+G+

Figure S2

Figure S2: RNA-seq analysis of H9-SRG cells [Related to Figure 2]

- **A.** Analysis of DEGs between sorted R+G- and R+G+ samples at naïve P1 (top) and P5 (bottom). n = 2 independent clones for each time point. Significance cutoffs were assigned as $Log_2(FC) \geq 2$ and $-Log_{10}(P\text{-value}) \geq 3$.
- **B.** Gene expression analysis of H9-SRG samples sorted by reporter activity using two independent clones.

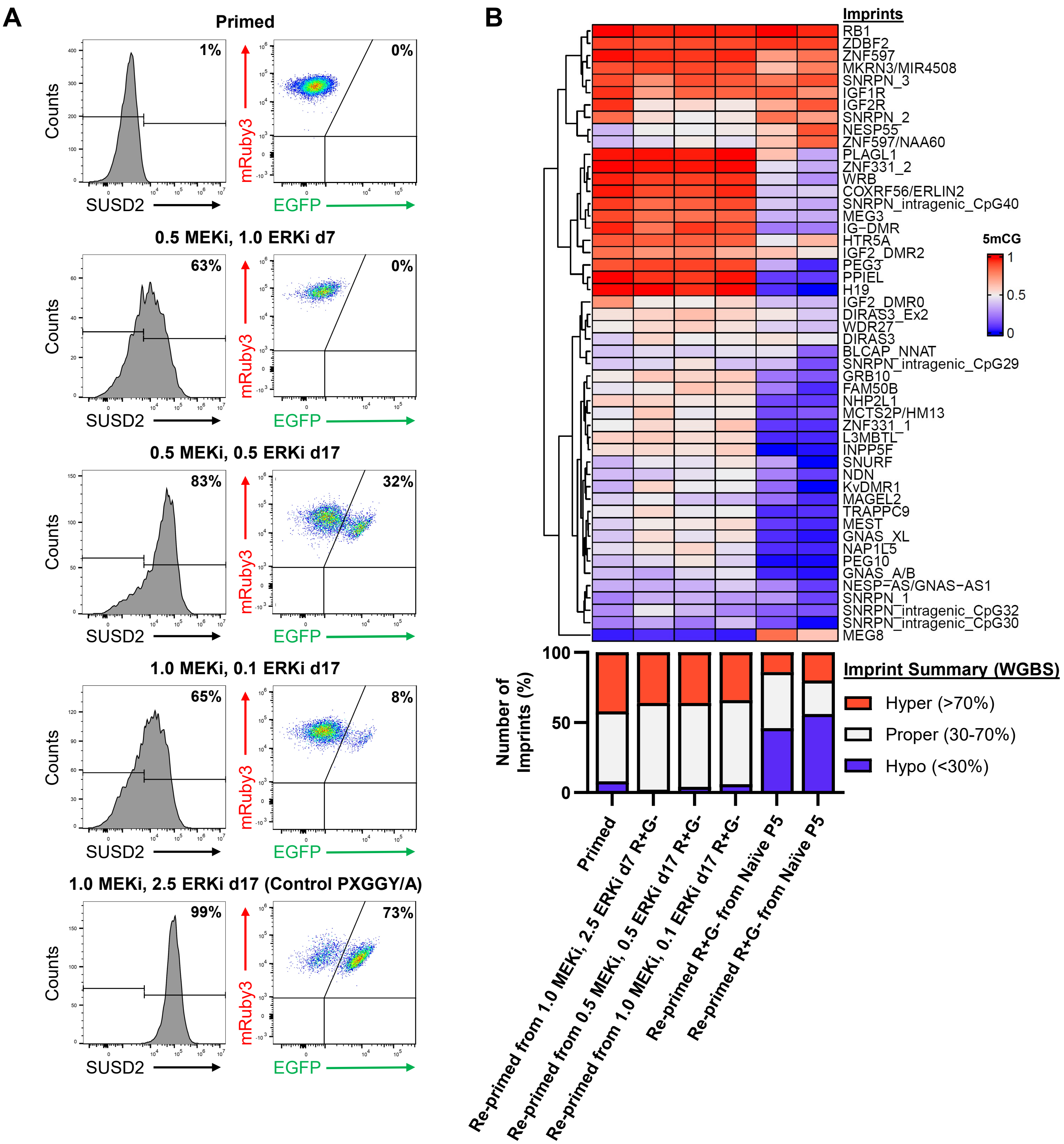
A
Primed
1%
0%
0.5 MEKi, 1.0 ERKi d7
63%
0%
0.5 MEKi, 0.5 ERKi d17
83%
32%
1.0 MEKi, 0.1 ERKi d17
65%
8%
1.0 MEKi, 2.5 ERKi d17 (Control PXGGY/A)
99%
73%
Counts
SUSD2
mRuby3
EGFP
B
Imprints
RB1
ZDBF2
ZNF597
MKRN3/MIR4508
SNRPN_3
IGF1R
IGF2R
SNRPN_2
NESP55
ZNF597/NAA60
PLAGL1
ZNF331_2
WRB
COXRF56/ERLIN2
SNRPN_intragenic_CpG40
MEG3
IG−DMR
HTR5A
IGF2_DMR2
PEG3
PPIEL
H19
IGF2_DMR0
DIRAS3_Ex2
WDR27
DIRAS3
BLCAP_NNAT
SNRPN_intragenic_CpG29
GRB10
FAM50B
NHP2L1
MCTS2P/HM13
ZNF331_1
L3MBTL
INPP5F
SNURF
NDN
KvDMR1
MAGEL2
TRAPPC9
MEST
GNAS_XL
NAP1L5
PEG10
GNAS_A/B
NESP−AS/GNAS−AS1
SNRPN_1
SNRPN_intragenic_CpG32
SNRPN_intragenic_CpG30
MEG8
5mCG
1
0.5
0
Number of Imprints (%)
100
50
0
Imprint Summary (WGBS)
Hyper (>70%)
Proper (30-70%)
Hypo (<30%)
Primed
Re-primed from 1.0 MEKi, 2.5 ERKi d7 R+G-
Re-primed from 0.5 MEKi, 0.5 ERKi d17 R+G-
Re-primed from 1.0 MEKi, 0.1 ERKi d17 R+G-
Re-primed R+G- from Naïve P5
Re-primed R+G- from Naïve P5

Figure S3

Figure S3: Methylation analysis of MEKi/ERKi titrated naïve cells followed by re-priming [Related to Figure 4]

A. Flow cytometry plots of selected inhibitor combinations and timings shown in Figure 4A. mRuby3/EGFP plots (right) were gated for SUSD2+ to show reporter activity of naïve-converting cells (excluding primed sample).

B. WGBS analysis and summary of imprints in H9-SRG re-primed samples following a brief titrated naïve pulse. Re-primed titrated samples (columns 2-4) were sorted for R+G-/SUSD2+ expression at the end of naïve treatment. When re-primed, these samples were >99.5% R^+G^-/SUSD2$^-$ and were therefore not sorted again. Re-primed titrated samples were compared to the primed (column 1) and re-primed R+G-/R+G+ samples (columns 5-6) from Figure 2.

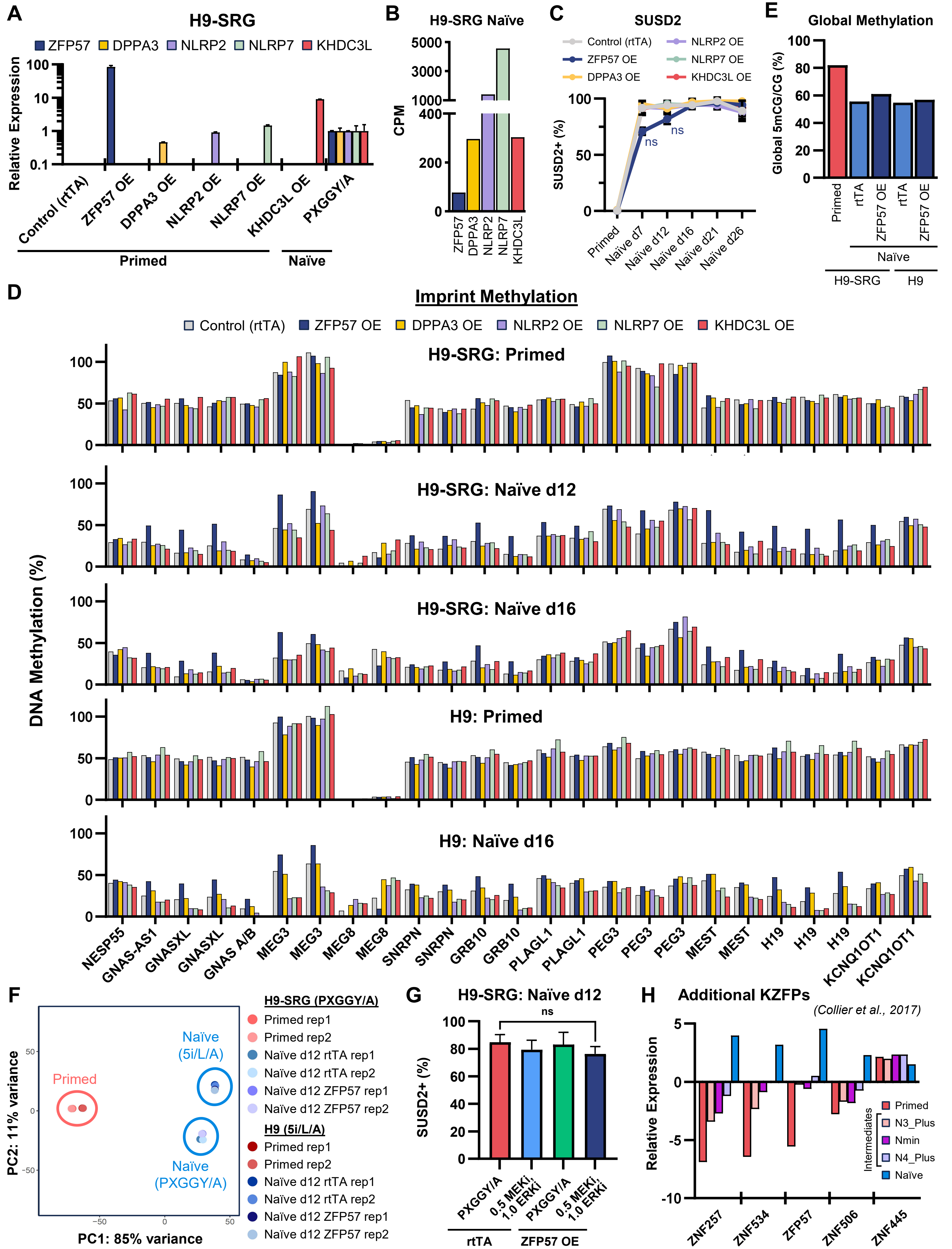
A
H9-SRG
ZFP57 DPPA3 NLRP2 NLRP7 KHDC3L
Relative Expression
Control (rtTA)
ZFP57 OE
DPPA3 OE
NLRP2 OE
NLRP7 OE
KHDC3L OE
PXGGY/A
Primed
Naïve
B
H9-SRG Naïve
CPM
C
SUSD2
Control (rtTA)
ZFP57 OE
DPPA3 OE
NLRP2 OE
NLRP7 OE
KHDC3L OE
SUSD2+ (%)
ns
E
Global Methylation
Global 5mCG/CG (%)
H9-SRG
H9
D
Imprint Methylation
DNA Methylation (%)
H9-SRG: Primed
H9-SRG: Naïve d12
H9-SRG: Naïve d16
H9: Primed
H9: Naïve d16
NESP55 GNAS-AS1 GNASXL GNASXL GNAS A/B MEG3 MEG3 MEG8 MEG8 SNRPN SNRPN GRB10 GRB10 PLAGL1 PLAGL1 PEG3 PEG3 PEG3 MEST MEST H19 H19 H19 KCNQ1OT1 KCNQ1OT1
F
H9-SRG (PXGGY/A)
Primed rep1
Primed rep2
Naïve d12 rtTA rep1
Naïve d12 rtTA rep2
Naïve d12 ZFP57 rep1
Naïve d12 ZFP57 rep2
H9 (5i/L/A)
Naïve (5i/L/A)
Naïve (PXGGY/A)
PC1: 85% variance
PC2: 11% variance
G
H9-SRG: Naïve d12
0.5 MEKi, 1.0 ERKi
rtTA
ZFP57 OE
H
Additional KZFPs
(Collier et al., 2017)
Intermediates
N3_Plus
Nmin
N4_Plus
ZNF257 ZNF534 ZFP57 ZNF506 ZNF445
Figure S4

Figure S4: Characterization of candidate imprint-protecting factor overexpression during primed-to-naïve resetting [Related to Figure 5]

- **A.** Gene expression analysis of candidate imprint-protecting factors in H9-SRG primed hPSCs. Samples were collected on the day of seeding for naïve resetting. Error bars represent standard deviation; n = 3.
- **B.** Expression of candidate imprint-protecting factors in H9-SRG naïve cells. Values were retrieved from RNA-seq data (Figure S2): Clone 1 Naïve P5 R+G+.
- **C.** Analysis of H9-SRG SUSD2 expression by flow cytometry during primed-to-naïve resetting of genetic overexpression samples. Error bars represent the standard error of the mean; n = 3 independent experiments. “ns” represents multiple unpaired t-test q-value > 0.1.
- **D.** MS-MLPA analysis of DNA methylation at imprints during primed-to-naïve resetting in each condition: H9-SRG cells at day 12 and 16 (PXGGY/A) and H9 cells at day 16 (5i/L/A).
- **E.** Global DNA methylation levels as measured by WGBS in control and ZFP57 overexpression samples during naïve resetting in PXGGY/A (H9-SRG, day 12) and 5i/L/A (H9, day 16).
- **F.** Principal component analysis of RNA-seq data from primed and naïve control and ZFP57 overexpression samples in PXGGY/A (H9-SRG, day 12) and 5i/L/A (H9, day 16).
- **G.** Analysis of the H9-SRG SUSD2+ population at day 12 of naïve resetting. Error bars represent the standard deviation; n = 3 independent experiments. “ns” represents unpaired t-test p-value > 0.1.
- **H.** Relative expression of KZFPs in primed, intermediate, and naïve cell populations (Collier *et al.*, 2017).

Table S1: Oligos used in this study

Name	Sequence (5' – 3')	Purpose
sgRNA-SNRPN	TCAACAGTATGCTAAGGTCTNGG	CRISPR/Cas9 targeting
SNRPN Left HA Forward (SNRPN_F)	AGGGCTTTTTGGATGTCAGTGT	Junction PCR/Sanger
SNRPN Right HA Reverse (SNRPN_R)	CATTCCCTCGCTGCCTACTA	Junction PCR
mRuby3_F	CACCAATTCAAATGCACAGG	Junction PCR/Sanger
mRuby3_R	ATTTGGCAACTGCCACTTCT	Junction PCR
EGFP_F	GGCAAGCTGACCCTGAAGTT	Junction PCR/Sanger
EGFP_R	TTCTGCTTGTCGGCCATGAT	Junction PCR
RPLP0_F	GCTTCCTGGAGGGTGTCC	qPCR
RPLP0_R	GGACTCGTTTGTACCCGTTG	qPCR
ZIC2_F	CCCTTCAAGGCCAAATACAA	qPCR
ZIC2_R	TGCATGTGCTTCTTCCTGTC	qPCR
SFRP2_F	ACGGCATCGAATACCAGAACA	qPCR
SFRP2_R	CTCGTCTAGGTCATCGAGGCA	qPCR
KLF17_F	CTGCCTGAGCGTGGTATGAG	qPCR
KLF17_R	TCATCCGGGAAGGAGTGAGA	qPCR
DNMT3L_F	TTCTGGATGTTCGTGGACAA	qPCR
DNMT3L_R	ACATCTGGGATGGTGACTGG	qPCR
ZFP57_F	ATGAAGAGAGATTGCTGGAGGG	qPCR
ZFP57_R	CCTCTGCATGCAAGGAAGAC	qPCR
DPPA3_F	GTTACTGGGCGGAGTTCGTA	qPCR
DPPA3_R	TGAAGTGGCTTGGTGTCTTG	qPCR
NLRP2_F	TCTGGACCTGGGTCAGAATC	qPCR
NLRP2_R	CCAGCAGCTTATTGAGTTCAT	qPCR
NLRP7_F	GGCCAGAATCATTTGTGGAA	qPCR
NLRP7_R	TTTTCTTTCACTTCCTCCAACA	qPCR
KHDC3L_F	CTAACGGCGAGGCTGAGAT	qPCR
KHDC3L_R	GCCTTTCCTGAGCCTTTCG	qPCR
ZNF445_F	CTACCGTCTGCACCGAGAA	qPCR
ZNF445_R	GCTTCTCCTGGGTGTGAATC	qPCR

Supplemental methods

Primed hPSC culture

H9 (WA09) hPSCs were obtained from the Genome Engineering & Stem Cell Center at the McDonnell Genome Institute of Washington University. Cell line identity was authenticated using STR profiling. GTW banding was performed after genome editing and showed a chromosomally normal karyotype. Primed hPSCs were cryopreserved in 10% DMSO solution and stored under liquid nitrogen in the vapor phase. Upon thawing, cells were warmed quickly, washed with appropriate medium, and plated for culture. Primed hPSCs were grown in mTeSR Plus medium on Matrigel-coated plates at 37°C, 20% O_2, and 5% CO_2. For general maintenance, cells were dissociated into small clumps using Dispase or ReLeSR and split at a ratio of 1:10-1:50. The medium was fully replaced every 1-2 days.

iMEF culture

Mouse embryonic fibroblasts (MEFs) were grown in fibroblast medium (DMEM/F12, 10% FBS, 1X GlutaMAX, and 1X Penicillin/Streptomycin) at 37°C, 20% O_2, and 5% CO_2. MEFs were growth-inactivated with 5 mg/mL mitomycin C for 3 hours. Inactivated MEFs (iMEFs) were single-cell dissociated with 0.25% Trypsin-EDTA and cryopreserved in 10% DMSO solution. For use as a feeder layer, iMEFs were thawed and plated at approximately 40,000 cells/cm^2.

Naïve media preparation

N2B27 basal medium was made using a 1:1 ratio of DMEM/F12:Neurobasal supplemented with 1X N2 supplement, 1X B27 supplement, 1X MEM non-essential amino acids solution, 1X GlutaMAX, 1% penicillin/streptomycin, 50 µg/mL BSA Fraction V, and 0.1 mM β-mercaptoethanol. PXGGY/A medium was made using N2B27 supplemented with 1 µM PD0325901, 2 µM XAV939, 2 µM Gö6983, 2.5 µM GDC-0994, 10 µM Y-27632, and 10 ng/mL Activin A (Khan et al., 2021). 5i/L/A medium was made using N2B27 supplemented with 1 µM PD0325901, 1 µM IM-12, 0.5

μM SB590885, 1 μM WH4-023, 10 μM Y-27632, 20 ng/mL recombinant human LIF, and 10 ng/mL Activin A (Fischer et al., 2022).

Primed-to-naïve hPSC resetting and naïve maintenance

Primed hPSCs were single-cell dissociated using TrypLE Express. hPSCs were seeded at a density of ~24,000 cells/cm^2 on iMEFs that were plated at least one day prior. Seeded cells were grown in mTeSR Plus supplemented with 10 μM Y-27632 for 2 days at 37°C, 5% O_2, and 5% CO_2. After 2 days, the medium was replaced with naïve medium, which designated day 0 of naïve resetting. Cultures were maintained at 37°C, 5% O_2, and 5% CO_2, and media were replaced every 1-2 days. Naïve hPSCs were split using TrypLE Express at a ratio of 1:1-1:4.

Re-priming naïve hPSCs

Naïve hPSCs were dissociated with TrypLE Express and seeded at ≥ 40,000 cells per well of a 6-well plate pre-coated with Matrigel. Cells were cultured in mTeSR Plus (supplemented with 10 μM Y-27632 for the first 24 hours) at 37°C, 20% O_2, and 5% CO_2. For the first passage, TrypLE Express was used to single-cell dissociate cells and mTeSR Plus was supplemented with 10 μM Y-27632 for 24 hours. For subsequent passages, ReLeSR was used to dissociate cells into small clumps and Y-27632 was omitted. Re-primed cells were used for analyses at >20 days of treatment.

Gene editing to establish the SNRPN dual-colored reporter

A single-guide RNA (sgRNA) targeting the *SNRPN* C-terminus and donor plasmids containing *P2A-mRuby3* and *P2A-EGFP* sequences were designed and validated by the Genome Engineering & Stem Cell Center at Washington University. Donor homology arms (left 796 bp; right 803 bp) were targeted to the regions flanking the *SNRPN* stop codon. H9 primed cells in a strong growth phase were single-cell dissociated with TrypLE Express. Cells were nucleofected

with 300 pmol sgRNA, 192 pmol Cas9 protein, and 1.5 µg of each donor plasmid using a Lonza 4-D nucleofector with P3 solution (Lonza, V4XP-3024). mTeSR Plus was supplemented with 10 µM Y-27632 for 24 hours post-nucleofection. Nine days after nucleofection, cells were single-cell sorted by FACS for mRuby3 or EGFP expression. No double-positive cells were detected. 1X RevitaCell (Gibco, A2644501) and 1X CloneR (STEMCELL Technologies, 05888) were used with mTeSR Plus to improve cell viability. Clones were expanded and integration was assessed by junction PCR. A clone containing one *P2A-mRuby3* allele and one wild-type allele (mRuby3/WT) was validated. This clone was nucleofected a second time using the sgRNA, Cas9 protein, and the *P2A-EGFP* donor plasmid. Cells were sorted at 5 cells/well to increase the likelihood of isolating the desired clone. Since properly-integrated EGFP would not be expressed in the primed hPSC state (as the maternal *SNRPN* allele is silenced), integration of EGFP was assessed by junction PCR. Two pools of cells showed the presence of *SNRPN-EGFP*. Both pools were single-cell sorted and screened again by junction PCR. Each pool provided a clone containing *SNRPN-mRuby3/SNRPN-EGFP*, which were deemed Clone 1 and Clone 2. Final sequences were validated by Sanger sequencing.

Live-cell imaging

Cells were imaged in culture media or FluoroBrite DMEM (Gibco, A1896701) using a Leica DMi8 inverted microscope. Scale bars were added using FIJI software (Schindelin et al., 2012).

Flow cytometry/FACS

Cells were single-cell dissociated using TrypLE Express and resuspended in their respective cell culture media. If desired, anti-human SUSD2-APC antibody (BioLegend, 327408) was used at a dilution of 1:100 for 45 minutes at 4°C in the dark. Cells were washed in Flow Buffer (1X HBSS + 5% FBS) to remove antibodies. Flow cytometry was performed in Flow Buffer using a Sony SY3200 Synergy cytometer (FACS-purification) or a Beckman Coulter CytoFLEX S cytometer

(analysis). Cells maintained in culture after FACS-purification were washed in 1X DPBS and culture media were supplemented with 10 µM Y-27632 (if not already in media) and 100 µg/mL Primocin for 24 hours. Data were processed with FlowJo v10.8.1 software.

Cell cycle analysis

Cells were single-cell dissociated using TrypLE Express, washed in 1X DPBS, and fixed in cold 70% ethanol for 30 min. Fixed cells were washed twice in 1X DPBS, then resuspended in 1 µg/mL DAPI (Thermo Scientific, 62248) in DPBS. Cells were incubated for 1 hour at room temperature and protected from light. Cells were not washed after DAPI staining. Flow cytometry was performed on a Beckman Coulter CytoFLEX S cytometer. Cell cycle analysis was performed using the Cell Cycle analysis tool in FlowJo v10.8.1 using a Watson (Pragmatic) fitting model.

Genomic DNA isolation

Genomic DNA (gDNA) was isolated from cells using the DNeasy Blood & Tissue Kit (Qiagen, 69504) per manufacturer's instructions.

MS-MLPA assay

MS-MLPA assays were performed on gDNA using reagents from MRC Holland Multilocus Imprinting Kits ME034-B1 or ME034-C1 according to manufacturer's instructions (Nygren et al., 2005). Briefly, 40 ng of gDNA were denatured and hybridized with the provided probemix for 16 hours. Samples were topped with 30 µL PCR-grade mineral oil to prevent evaporation. Each sample was then split in half. The first half underwent ligation, while the second half underwent ligation + digestion with HhaI enzyme. Products were amplified by PCR, then analyzed by capillary electrophoresis on an Applied Biosystems 3730xl Fragment Analyzer using the 6-FAM label. Peaks were called with GeneMapper v6.0 and inspected manually. The area under each peak was used to calculate DNA methylation levels.

Bulk RNA-sequencing and analysis

Total RNA integrity was determined using an Agilent Bioanalyzer or 4200 Tapestation. Library preparation was performed with 10 ng of total RNA with a Bioanalyzer RIN score greater than 8.0. ds-cDNA was prepared using the SMARTer Ultra Low RNA kit for Illumina Sequencing (Takara) per manufacturer's protocol. cDNA was fragmented using a Covaris E220 sonicator using peak incident power 18, duty factor 20%, cycles per burst 50 for 120 seconds. cDNA was blunt ended, had an A base added to the 3' ends, and then had Illumina sequencing adapters ligated to the ends. Ligated fragments were then amplified for 12-15 cycles using primers incorporating unique dual index tags. Fragments were sequenced on an Illumina NovaSeq-6000 (Figure 2) or an Illumina NovaSeq X Plus (Figures 4-5) using paired end reads extending 150 bases. Basecalls and demultiplexing were performed with Illumina's bcl2fastq software and a custom python demultiplexing program with a maximum of one mismatch in the indexing read. RNA-seq reads were then aligned to the Ensembl release 76 (Figure 2) or 101 (Figures 4-5) primary assembly with STAR version 2.5.1a or 2.7.9a (Dobin et al., 2013). Gene counts were derived from the number of uniquely aligned unambiguous reads by Subread:featureCount version 1.4.6-p5 or 2.0.3 (Liao et al., 2014). Isoform expression of known Ensembl transcripts were estimated with Salmon version 0.8.2 or 1.5.2 (Patro et al., 2017). Sequencing performance was assessed for the total number of aligned reads, total number of uniquely aligned reads, and features detected. The ribosomal fraction, known junction saturation, and read distribution over known gene models were quantified with RSeQC version 2.6.2 or 4.0 (Wang et al., 2012).

All gene counts were then imported into the R/Bioconductor package EdgeR (Robinson et al., 2010) and TMM normalization size factors were calculated to adjust for samples for differences in library size. Ribosomal genes and genes not expressed in the smallest group size minus one samples greater than one count-per-million were excluded from further analysis. The TMM size

factors and the matrix of counts were then imported into the R/Bioconductor package Limma (Ritchie et al., 2015). Weighted likelihoods based on the observed mean-variance relationship of every gene and sample were then calculated for all samples and the count matrix was transformed to moderated log 2 counts-per-million with Limma's voomWithQualityWeights (Liu et al., 2015). The performance of all genes was assessed with plots of the residual standard deviation of every gene to their average log-count with a robustly fitted trend line of the residuals.

Differential expression analysis was performed to analyze for differences between conditions. The R/Bioconductor package EnhancedVolcano version 1.12.0 (Blighe et al., 2021) was used to plot log 2 fold changes versus negative log 10 P-values for each term. Principal component analysis was performed using DESeq2 version 1.34.0 (Love et al., 2014). Using results filtered for only those genes with Benjamini-Hochberg false-discovery rate adjusted p-values less than or equal to 0.05, Gene Ontology (GO) terms, MSigDb, and KEGG pathways were detected using the R/Bioconductor package GAGE (Luo et al., 2009) to test for changes in expression of the reported log 2 fold changes reported by Limma in each term versus the background log 2 fold changes of all genes found outside the respective term. Bar plots to visualize GO terms were made using ggplot2 version 3.4.4 (Wickham, 2016). Heatmaps of log 2 fold changes were plotted using the R/Bioconductor package ComplexHeatmap version 2.10.0 (Gu et al., 2016). Gene set enrichment analysis was performed by ranking all genes using the R/Bioconductor package fgsea (Korotkevich et al., 2019). Enrichment for MSigDb Hallmark gene sets was performed with GSEA version 4.3.3 (Subramanian et al., 2005) using the pre-ranked gene list and 50,000 permutations.

WGBS analysis

Paired-end Fastq files were trimmed with TrimGalore version 0.6.6 (Krueger et al., 2020) and reads were aligned with Bismark version 0.23.0 (Krueger and Andrews, 2011). The Methpipe package version 4.1.1 was then used with the indicated tools to remove duplicate reads

(duplicate-remover), calculate methylation levels (methcounts), extract and merge symmetric CpGs (symmetric-cpgs), and calculate methylation statistics for regions of interest (roimethstat) (Song et al., 2013). Heatmaps of imprinted regions (Court et al., 2014) were plotted using the R/Bioconductor package ComplexHeatmap version 2.10.0 (Gu *et al.*, 2016).

Browser tracks

FastqQC version 0.11.9 was used to assess the quality of the raw reads. Subsequently, the paired-end reads were trimmed to remove adaptor sequences and low-quality reads with Cutadapt version 4.0 and reassessed using FastqQC. The human reference genome hg38 was first bisulfite converted using Bismark version 0.20.0 (Krueger and Andrews, 2011). The paired-end reads were aligned to the hg38 bisulfite-converted genome and deduplicated using 'deduplicate_bismark'. DNA methylation levels were calculated using 'bismark_methylation_extractor' and displayed in a methylC format on the WashU Epigenome Browser (Zhou et al., 2011).

KZFP analysis

The cutoff for additional KZFPs of interest was set at "Naïve" - "N3_Plus" > 3 (Collier et al., 2017).

Supplemental references

Blighe, K., Rana, S., and Lewis, M. (2021). EnhancedVolcano: Publication-ready volcano plots with enhanced colouring and labeling. 10.18129/B9.bioc.EnhancedVolcano.

Collier, A.J., Panula, S.P., Schell, J.P., Chovanec, P., Plaza Reyes, A., Petropoulos, S., Corcoran, A.E., Walker, R., Douagi, I., Lanner, F., and Rugg-Gunn, P.J. (2017). Comprehensive Cell Surface Protein Profiling Identifies Specific Markers of Human Naive and Primed Pluripotent States. Cell stem cell *20*, 874-890 e877. 10.1016/j.stem.2017.02.014.

Court, F., Tayama, C., Romanelli, V., Martin-Trujillo, A., Iglesias-Platas, I., Okamura, K., Sugahara, N., Simón, C., Moore, H., Harness, J.V., et al. (2014). Genome-wide parent-of-origin DNA methylation analysis reveals the intricacies of human imprinting and suggests a germline methylation-independent mechanism of establishment. Genome Research *24*, 554-569. 10.1101/gr.164913.113.

Dobin, A., Davis, C.A., Schlesinger, F., Drenkow, J., Zaleski, C., Jha, S., Batut, P., Chaisson, M., and Gingeras, T.R. (2013). STAR: ultrafast universal RNA-seq aligner. Bioinformatics *29*, 15-21. 10.1093/bioinformatics/bts635.

Fischer, L.A., Khan, S.A., and Theunissen, T.W. (2022). Induction of Human Naïve Pluripotency Using 5i/L/A Medium. In Human Naïve Pluripotent Stem Cells, P. Rugg-Gunn, ed. (Springer US), pp. 13-28. 10.1007/978-1-0716-1908-7_2.

Gu, Z., Eils, R., and Schlesner, M. (2016). Complex heatmaps reveal patterns and correlations in multidimensional genomic data. Bioinformatics *32*, 2847-2849. 10.1093/bioinformatics/btw313.

Khan, S.A., Park, K.-m., Fischer, L.A., Dong, C., Lungjangwa, T., Jimenez, M., Casalena, D., Chew, B., Dietmann, S., Auld, D.S., et al. (2021). Probing the signaling requirements for naive human pluripotency by high-throughput chemical screening. Cell Reports *35*, 109233. 10.1016/j.celrep.2021.109233.

Korotkevich, G., Sukhov, V., and Sergushichev, A. (2019). Fast gene set enrichment analysis. bioRxiv. 10.1101/060012.

Krueger, F., and Andrews, S.R. (2011). Bismark: a flexible aligner and methylation caller for Bisulfite-Seq applications. Bioinformatics *27*, 1571-1572. 10.1093/bioinformatics/btr167.

Krueger, F., James, F., Ewels, P., Afyounian, E., Weinstein, M., Schuster-Boeckler, B., and Hulselmans, G. (2020). TrimGalore v0.6.6.

Liao, Y., Smyth, G.K., and Shi, W. (2014). featureCounts: an efficient general purpose program for assigning sequence reads to genomic features. Bioinformatics *30*, 923-930. 10.1093/bioinformatics/btt656.

Liu, R., Holik, A.Z., Su, S., Jansz, N., Chen, K., Leong, H.S., Blewitt, M.E., Asselin-Labat, M.-L., Smyth, G.K., and Ritchie, M.E. (2015). Why weight? Modelling sample and observational level variability improves power in RNA-seq analyses. Nucleic Acids Research *43*, e97-e97. 10.1093/nar/gkv412.

Love, M.I., Huber, W., and Anders, S. (2014). Moderated estimation of fold change and dispersion for RNA-seq data with DESeq2. Genome Biology *15*, 550. 10.1186/s13059-014-0550-8.

Luo, W., Friedman, M.S., Shedden, K., Hankenson, K.D., and Woolf, P.J. (2009). GAGE: generally applicable gene set enrichment for pathway analysis. BMC Bioinformatics *10*, 161. 10.1186/1471-2105-10-161.

Nygren, A.O.H., Ameziane, N., Duarte, H.M.B., Vijzelaar, R.N.C.P., Waisfisz, Q., Hess, C.J., Schouten, J.P., and Errami, A. (2005). Methylation-Specific MLPA (MS-MLPA): simultaneous detection of CpG methylation and copy number changes of up to 40 sequences. Nucleic Acids Research *33*, e128-e128. 10.1093/nar/gni127.

Patro, R., Duggal, G., Love, M.I., Irizarry, R.A., and Kingsford, C. (2017). Salmon provides fast and bias-aware quantification of transcript expression. Nature Methods *14*, 417-419. 10.1038/nmeth.4197.

Ritchie, M.E., Phipson, B., Wu, D., Hu, Y., Law, C.W., Shi, W., and Smyth, G.K. (2015). limma powers differential expression analyses for RNA-sequencing and microarray studies. Nucleic Acids Research *43*, e47-e47. 10.1093/nar/gkv007.

Robinson, M.D., McCarthy, D.J., and Smyth, G.K. (2010). edgeR: a Bioconductor package for differential expression analysis of digital gene expression data. Bioinformatics *26*, 139-140. 10.1093/bioinformatics/btp616.

Schindelin, J., Arganda-Carreras, I., Frise, E., Kaynig, V., Longair, M., Pietzsch, T., Preibisch, S., Rueden, C., Saalfeld, S., Schmid, B., et al. (2012). Fiji: an open-source platform for biological-image analysis. Nature Methods *9*, 676-682. 10.1038/nmeth.2019.

Song, Q., Decato, B., Hong, E.E., Zhou, M., Fang, F., Qu, J., Garvin, T., Kessler, M., Zhou, J., and Smith, A.D. (2013). A Reference Methylome Database and Analysis Pipeline to Facilitate Integrative and Comparative Epigenomics. PLOS ONE *8*, e81148. 10.1371/journal.pone.0081148.

Subramanian, A., Tamayo, P., Mootha, V.K., Mukherjee, S., Ebert, B.L., Gillette, M.A., Paulovich, A., Pomeroy, S.L., Golub, T.R., Lander, E.S., and Mesirov, J.P. (2005). Gene set enrichment analysis: A knowledge-based approach for interpreting genome-wide expression profiles. Proceedings of the National Academy of Sciences *102*, 15545-15550. 10.1073/pnas.0506580102.

Wang, L., Wang, S., and Li, W. (2012). RSeQC: quality control of RNA-seq experiments. Bioinformatics *28*, 2184-2185. 10.1093/bioinformatics/bts356.

Wickham, H. (2016). ggplot2: Elegant Graphics for Data Analysis (Springer-Verlag New York).

Zhou, X., Maricque, B., Xie, M., Li, D., Sundaram, V., Martin, E.A., Koebbe, B.C., Nielsen, C., Hirst, M., Farnham, P., et al. (2011). The Human Epigenome Browser at Washington University. Nature Methods *8*, 989-990. 10.1038/nmeth.1772.
